# Carborane‐Decorated Siloles with Highly Efficient Solid‐State Emissions – What Drives the Photophysical Properties?

**DOI:** 10.1002/chem.202404462

**Published:** 2025-01-28

**Authors:** Balázs Szathmári, Dóra Hessz, Dániel Zámbó, Clemens Bruhn, Rudolf Pietschnig, Antal Udvardy, Pál Szabó, Tamás Holczbauer, Marcell J. Balogh, Zsolt Kelemen

**Affiliations:** ^1^ Department of Inorganic and Analytical Chemistry Budapest University of Technology and Economics Műegyetem rkp. 3 H-1111 Budapest Hungary; ^2^ Department of Physical Chemistry and Materials Science and MTA-BME Lendület Quantum Chemistry Research Group Budapest University of Technology and Economics Műegyetem rkp. 3 H-1111 Budapest Hungary; ^3^ Institute of Technical Physics and Materials Science HUN-REN Centre for Energy Research Konkoly-Thege Miklós út 29–33 H-1121 Budapest Hungary; ^4^ Institute of Chemistry and CINSat University of Kassel Heinrich-Plett-Straße 40 34132 Kassel Germany; ^5^ Department of Physical Chemistry University of Debrecen Egyetem tér 1 H-4032 Debrecen Hungary; ^6^ Centre for Structural Science HUN-REN Research Centre for Natural Sciences Magyar tudósok körútja 2 H-1117 Budapest Hungary; ^7^ Chemical Crystallography Research Laboratory and Stereochemistry Research Group Institute for Organic Chemistry HUN-REN Research Centre for Natural Sciences Magyar Tudósok körútja 2 A H-1117 Budapest Hungary

**Keywords:** Aggregation-induced emission (AIE), Solid-state fluorescence, Carboranes, Siloles, TD-DFT calculations

## Abstract

New hybrids were synthesised by linking carboranes and siloles, both of which are known as aggregation‐induced emission active units. Although most of the newly synthesised systems do not display notable quantum yield either in solution or in the aggregated state, they emit strongly in the solid‐state, and a quantum yield of up to 100 % can be achieved. The tailorable quantum yield can be attributed to the packing of the molecules in the crystal lattice ruled by the carborane and phenyl moieties according to the SC‐XRD data. Our experimental results, complemented by density functional theory calculations, show that the silole moiety primarily influences the photophysical properties. At the same time, the carborane serves as a steric building block without direct responsibility for the aggregation‐induced emission property. The patterns of substituents can alter the absorption and emission properties.

## Graphical abstract

Two aggregation‐induced emission‐active units were combined to create new hybrids. The photophysical behaviour is dominated by the silole unit, while the solid‐state emission can be tailored via the supramolecular structure influenced by the carborane moiety.

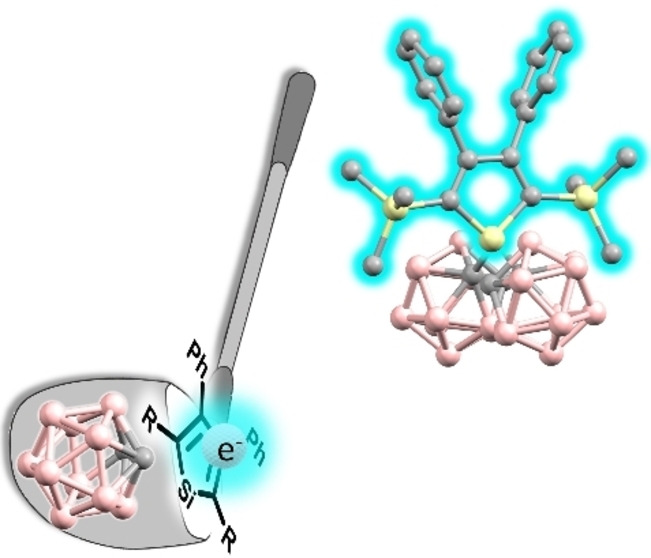

## Introduction

Aggregation‐induced emission (AIE) is a unique photophysical phenomenon where compounds are found to be weakly or not fluorescent in diluted solution but highly fluorescent when aggregated.[^1^, ^2^] It can be considered an abnormal behaviour since increasing the concentration of most luminogens (in solution or in solid‐state) drastically decreases the photoluminescence efficiency. Luminogens with AIE properties have received intense current research interest due to their promising possibilities in material science and biological technology.^[3]^

2,3,4,5‐tetraphenylsiloles are the archetype of AIE‐active systems,^[4]^ followed by several silole‐based systems with diverse functionalities.^[5]^ Besides siloles, several new systems have appeared during the last two decades.[^2^, ^6^, ^7^, ^8^, ^9^] In most AIE active systems, the intramolecular motions, especially the rotations of the substituents, serve as a very efficient non‐radiative decay channel for its excited states in solutions. In aggregated or solid‐state, these motions are significantly suppressed, which blocks the non‐radiative relaxation channels and promotes the radiative decay of the excited state. Therefore, the photoluminescence properties can prevail. For example, in the case of the above‐mentioned propeller‐like silole derivatives, the rotation of the substituents is mainly responsible for the AIE properties. It should be noted that other mechanisms were explored as well, such as J‐aggregate formation, twisted intramolecular charge transfer, and excited‐state intramolecular proton transfer.^[2]^

Carboranes with fluorescent properties^[10]^ emerged as an important “element‐block” of light‐emitting materials.^[11]^ These icosahedral clusters of formula C_2_B_10_H_12_ (*ortho*, *meta*, and *para* isomers can be distinguished according to the position of carbon atoms) can enhance the rigidity of the conjugated molecules and prevent the π–π interactions between them. As it was demonstrated by Teixidor and Núñez, the photoluminescence properties strongly depend on the isomer of the carborane unit[^12^, ^13^, ^14^, ^15^, ^16^] which acts as a strong electron‐withdrawing group. This effect is the strongest in the case of the *o*‐carborane, where the photoinduced intramolecular charge transfer (ICT) process can occur from the π‐conjugated fragments to the antibonding orbital mainly placed between the two cluster carbon atoms. Due to the population of the antibonding orbital, the C−C bond significantly elongates or even breaks,[^17^, ^18^, ^19^] which is primarily responsible for the AIE activity of these systems, which was experimentally verified by Chujo.^[20]^ This contrasts with silole derivatives, where the rotation of the substituents is responsible for the AIE activity.

**Scheme 1:**
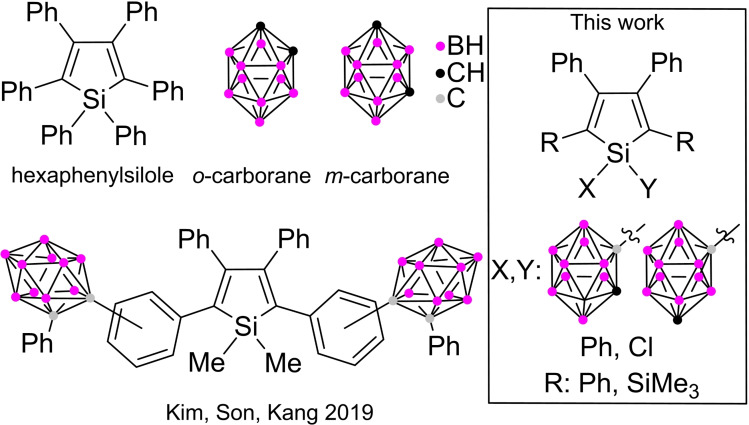
Left: parent siloles and carboranes systems, right: investigated systems in this work.

Despite the extensive research on AIE‐active species, there have been very few examples of incorporating more AIE‐active moieties within a single molecular system.[^21^, ^22^, ^23^, ^24^, ^25^, ^26^, ^27^, ^28^] Kim, Son and Kang reported phenyl‐siloles with solid‐state photochromism, where the *o*‐carborane moieties were introduced to the phenyl substituents at 2,5‐positions. The unique properties of the systems were attributed to the interaction between the silole and *o*‐carborane units through the interconnecting phenyl ring.^[29]^ Due to the linker, the degree of the rotation freedom is higher. Since the direct communication between 2D and 3D moieties may have some limitations,[^30^, ^31^, ^32^, ^33^] we planned to investigate systems where the carborane units are directly connected to the silole rings (Scheme 1). To further broaden the scope and limitation, in addition to the well‐studied phenyl‐substituted siloles, we examined systems with trimethylsilyl groups introduced at the 2 and 5 positions, which are known to quench effectively the fluorescence in both solution and aggregated state.^[34]^ It is worth considering whether the introduction of the carborane unit can recover the fluorescence of these systems.

## Results and Discussion

### Synthesis

**Scheme 2:**
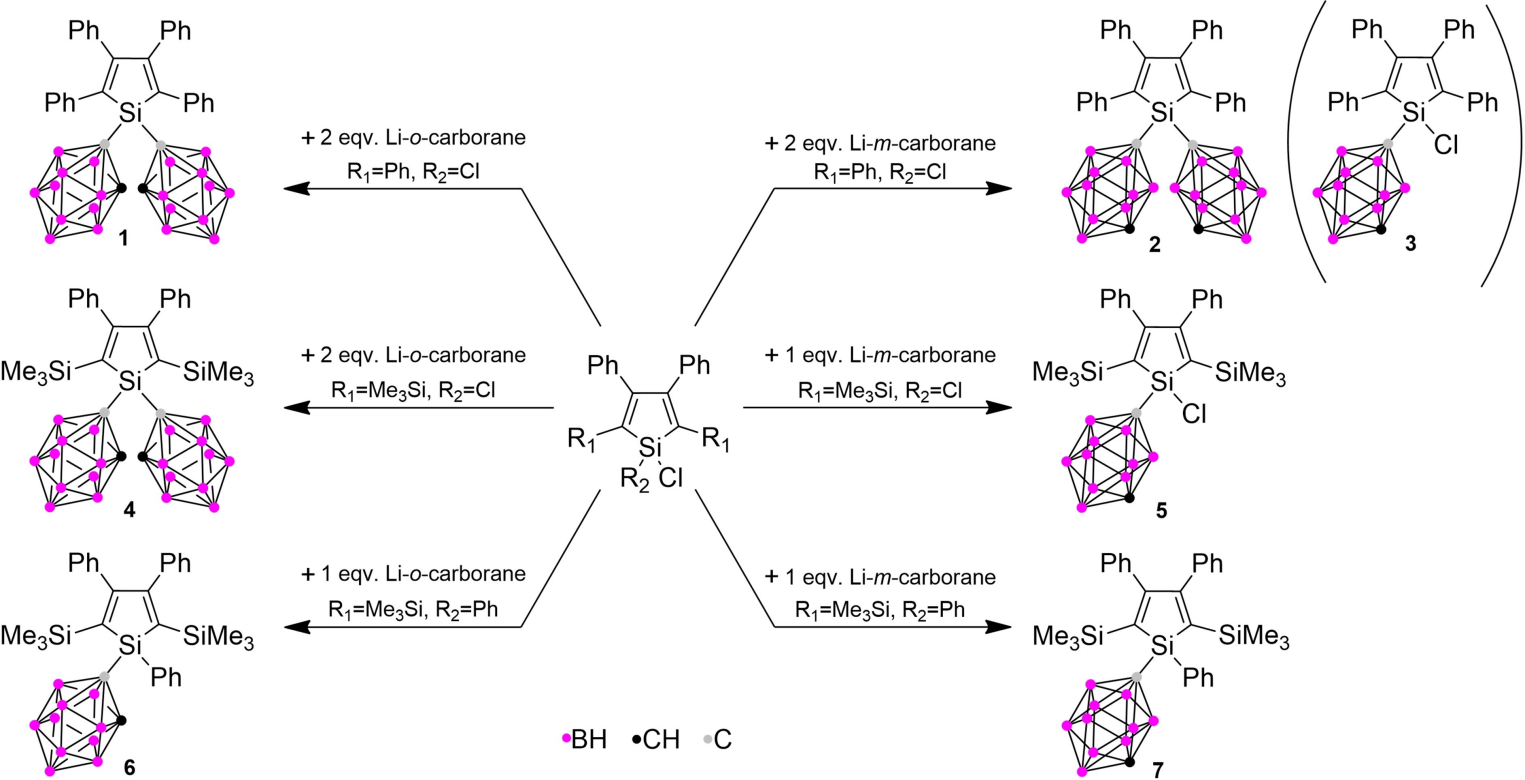
Synthetic routes to compounds **1**–**7**.

In our approach to synthesise the desired silole‐carborane conjugates, the corresponding chloro‐silole derivative was planned to react with lithiated carboranes (Scheme 2). The starting chloro‐siloles and dichloro‐siloles with SiMe_3_ functionalisation were prepared according to Tamao's produce,^[35]^ while the 1,1‐dichloro‐2,3,4,5‐tetraphenylsilole was prepared from 1,4‐dilithio‐1,2,3,4‐tetraphenyl‐butadiene and SiCl_4_.^[36]^ The coupling of the silole and carborane moiety was monitored by ^1^H NMR following the appreciable changes in C−H chemical shifts of the carborane unit. First, two equivalents of lithiated *o‐*carborane were reacted with 1,1‐dichloro‐2,3,4,5‐tetraphenylsilole in THF at room temperature. Investigation of the obtained greenish crystalline material by ^1^H NMR established the formation of the desired product, but the reaction mixture also contains several other byproducts. The compound continuously decomposed during the purification (extraction, column chromatography, crystallisation); therefore, its isolation and proper characterisation remained unsuccessful. Despite these facts, a small crop of single crystals from **1** suitable for SC‐XRD analysis was obtained, which verified the chemical composition of **1**. Using the same procedure but starting from lithiated *m‐*carborane, we were able to isolate the double‐substituted compound (**2**). It is worth to note, that we have obtained a small amount of side product (**3**) during the workup, which can be assigned to the corresponding monosubstituted counterpart with one of the chlorines remaining at the silole unit.

In order to explore the role of the substituents at different positions, we investigated the systems where the phenyl groups were replaced by trimethylsilyl groups. They do not only act as a bulky substituent but effectively quench the fluorescence of the silole unit in any state.^[34]^ The question arises whether introducing a carborane moiety can recover the fluorescence properties of the parent silole unit. Starting from the 1,1‐dichloro‐2,5‐bis(trimethylsilyl)‐3,4‐diphenylsilole and using lithiated *o*‐carborane, the corresponding product (**4**) formed. On the other hand, only monosubstituted counterpart (**5**) can be isolated starting from lithiated *m*‐carborane, which is in agreement with the lower reactivity observed in the case of tetraphenyl derivatives (**3**). All of our attempts to get the corresponding double substituted compound – such as using more equivalent of *m*‐carborane or elevated temperature – failed in our hand. These results suggest decreased reactivity of the silicon centre after introducing one carborane unit, which was further bolstered by the fact that **5** is air‐stable despite the presence of a Si−Cl bond. To investigate the effect of the carborane moiety compared to a phenyl group,^[37]^ 1‐carboranyl‐1‐phenylsilole derivatives were synthesised (**6**, **7**) as well using 1‐chloro‐2,5‐bis(trimethylsilyl)‐1,3,4‐triphenylsilole as a starting material. All new compounds (with the exception of **1** and **3**) were characterised by multinuclear (^1^H, ^11^B, ^11^B{^1^H}, ^13^C{^1^H}, ^29^Si{^1^H}) NMR, IR spectroscopy and HR‐MS as well (more details in the SI). The IR spectra of all compounds verified the presence of the carborane units, showing typical υ(B−H) bands between 2561 and 2626 cm^−1^. In the ^1^H NMR, the carborane C−H peak shows a downfield shift in the case of *o*‐carborane but not in the case of *m*‐carborane derivatives (compared to the starting carborane), which can be attributed to the relative positions of the carbon atoms in the cluster. The ^29^Si NMR chemical shifts of the ring‐silicon in the case of these compounds are in the range from −1.1 to −19.5 ppm, which is typical of the mono‐ and di‐alkyl, aryl‐substituted siloles.[^38^, ^39^, ^40^] Finally, the chemical structure of the obtained systems was also underlined by SC‐XRD data.

### Single Crystal X‐Ray Diffraction Study

**Figure 1:**
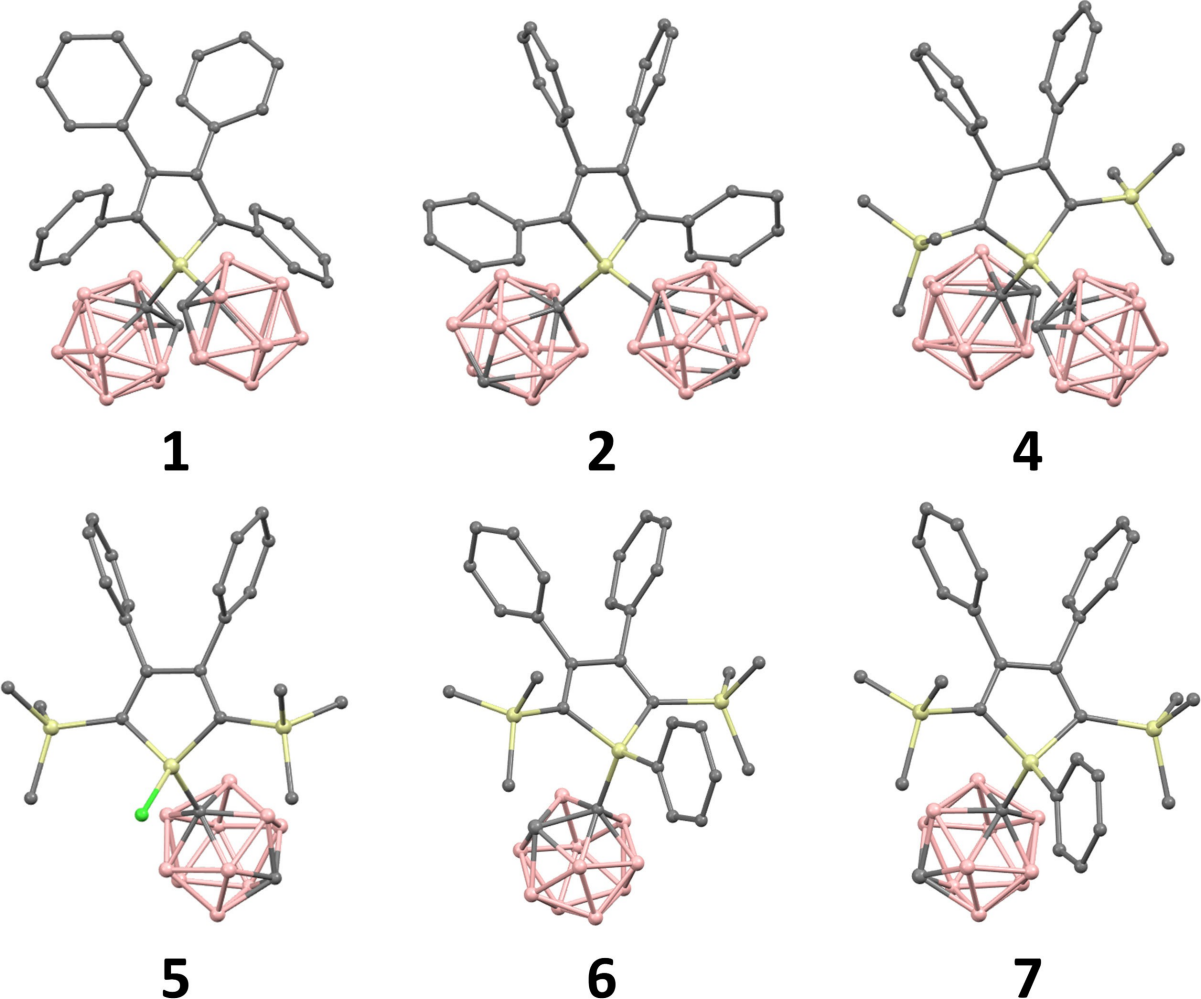
Ball and stick representation of the molecular structure of **1**, **2**, **4**, **5**, **6**, and **7**. Hydrogens and solvent molecules are omitted for clarity.

Single crystals suitable for X‐ray structural determination of compounds **1**, **2**, **4**, **5**, **6**, and **7** were obtained by slow evaporation from acetone, dichloromethane, THF, or solvent mixtures, respectively (more details in the SI). The molecular structures for all these compounds show a typical icosahedral geometry. The X‐ray structures of **1** and **2** contain one solvent molecule, which was omitted for better clarity in Figure 1. The Si−C_c_ bonds are in the range between 1.903 and 1.925 Å, which are much longer than the typical Si−C(sp^3^) bond (1.84 Å),^[41]^ however, it is similar to related carborane species featuring Si−C_c_ bonds (1.926±0.022 Å, according to a Cambridge Structural Database search conducted on November 20, 2024). The C_c/Ph_−Si−C_c_ angle is around 115° in the case of all investigated systems (in the case of **5**, the Cl−Si−C_c_ angle is 107°), demonstrating that both carborane moieties and the phenyl ring have similar space filling.^[34]^ In the case of the *o*‐carboranyl substituents containing **1**, **4**, and **6**, the C−C bond lengths are slightly elongated (1.665–1.687 Å) compared with the parent *o*‐carborane (∼1.63 Å),^[42]^ which can be attributed to the electron donor character of the silole unit. Due to the presence of bulky carboranyl and silyl substituents, a certain degree of steric strain between these two sets of ligands can be observed in case 4. The congestion decreases if one of the carboranyl substituents is replaced by phenyl rings. In this scenario, the silyl substituents are not in the plane of the silole ring, and the C−Si_*ring*_−C−Si_*silyl*_ dihedral angles are around 16°.

### Photophysical Properties and DFT Calculations

**Figure 2:**
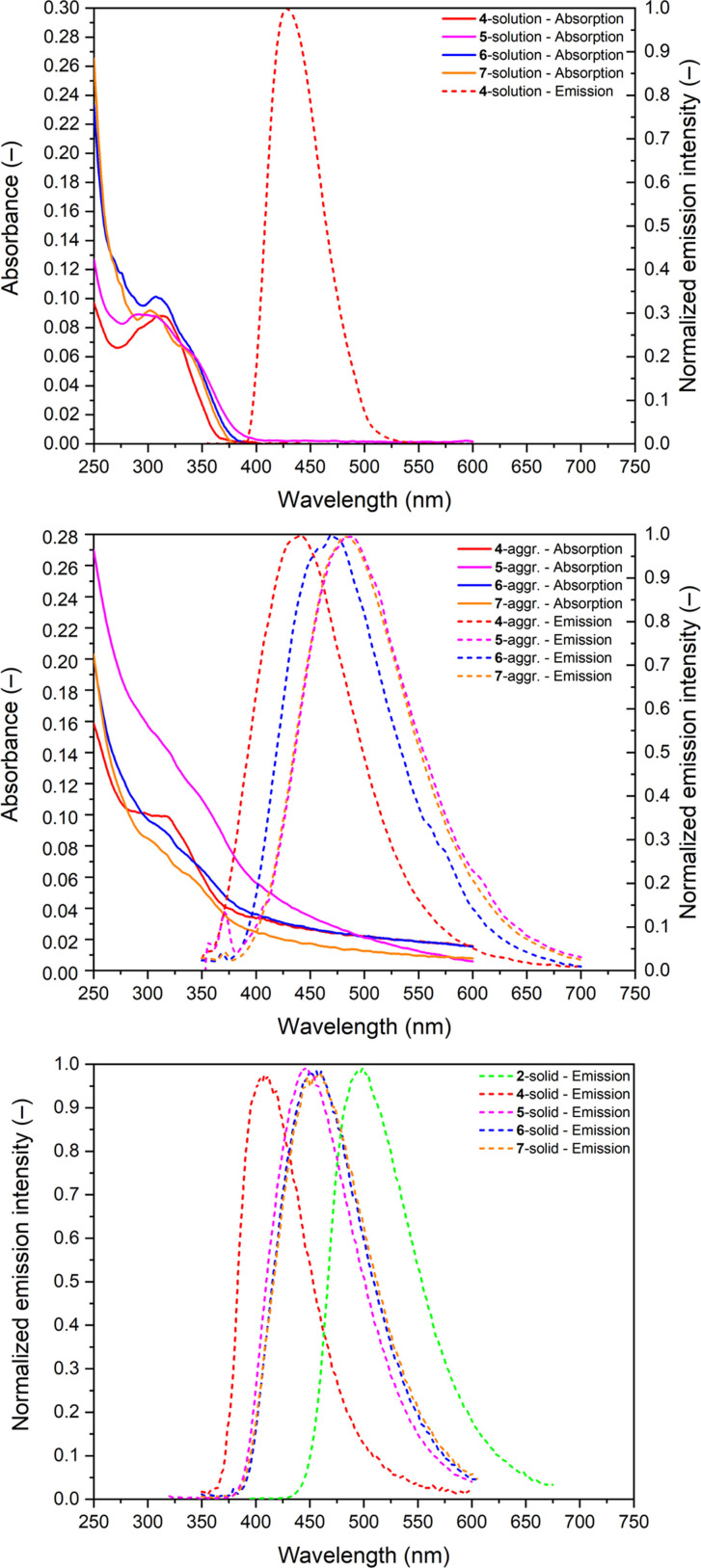
Absorption and emission spectra of **2**, **4**, **5**, **6** and **7** in ∼10^−5^ M THF solution (top), in THF/water v/v=1/99∼10^−5^ M aggregated state (middle) and in solid‐state (bottom).

The photophysical properties of **2**, **4**, **5**, **6**, and **7** were determined by UV‐Vis absorption and fluorescence spectroscopy in solvents with different polarity (acetonitrile, THF, hexane) in a mixture of THF/water (v/v=1/99) to form aggregates, as well as in the solid‐state (Table 1 and Figure 2). Unfortunately, in the case of compound **2**, continuous decomposition was observed in the solution. The impurities remain invisible in ^1^H NMR spectra, but the proper photophysical characterisation of **2** in solution is impossible. Investigating the emission spectra (Figure S1 in the SI), one may think about dual emission at first glance, which was previously described in related compounds,[^43^, ^44^, ^45^] but thin‐layer chromatography and the excitation spectra verified the presence of impurities possessing strong blue fluorescence (Figure S2–3 in the SI).

**Table 1:** Absorption/excitation wavelengths, emission wavelengths, and quantum yield values of compounds **2**, **4**, **5**, **6**, and **7** in THF solution, aggregated state (THF/water v/v=1/99), and solid‐state.

Compound	THF solution			Aggregated state			Solid‐state		
	λ_abs_ (nm)	λ_em_ (nm)	ϕ_F_^[a]^ (%)	λ_exc_ (nm)	λ_em_ (nm)	ϕ_F_^[a]^ (%)	λ_exc_ (nm)	λ_em_ (nm)	ϕ_F_^[b]^ (%)
**2**	–	–	–	–	–	–	377	500	53
**4**	311	428	<1	315	442	4	304	412	9
**5**	291	–	–	302	483	<1	302	449	55
**6**	308	–	–	300	470	2	309	456	86
**7**	302	–	–	302	484	2	302	459	100

[a] Relative quantum yield, reference is quinine‐sulfate (0.1 M H_2_SO_4_, *Φ*_F_=0.54); [b] Absolute quantum yield.

Compounds **4**, **5**, **6**, and **7** have very similar absorption spectra with a wide band around 300 nm, which can be assigned to the corresponding π‐π* transition of the silole moiety. It is worth highlighting that the UV‐Vis spectra of the investigated compound do not alter by changing solvents (Figure S4 in the SI). Therefore, it does not depend on the polarity of the medium, suggesting a local excitation mode. Investigating the photoluminescence emission properties of **4**, **5**, **6**, and **7**, it can be established that **4** shows very weak but observable fluorescence (quantum yield is below 1 %), **5**, **6**, and **7** do not exhibit any detectable photoluminescence in diluted solution, indicating the emission is very effectively quenched. Similar to the absorption photoluminescence (PL) emission spectra, no solvent dependence was observed (Figure S4 in the SI).

As a next step, we have prepared samples to investigate aggregated states; THF solutions of **4**–**7** were diluted one hundred times by water, obtained turbid mixture. Investigating the photophysical properties of these samples, it can be established that the absorption spectra do not change significantly; the common broadening can be observed. Investigating the emission spectra, it can be stated that the fluorescence of **4**–**7** can be recovered. Despite the quantum yield increasing, indicating aggregation‐induced emission properties, it remained negligible (<5 %) in the aggregate state for compounds **4**, **6**, and **7**. This observation may be attributed to the presence of SiMe_3_ substituents at the silole units in cases **4**, **5**, **6**, and **7**, which effectively quench the fluorescence even in the aggregate state.^[34]^

**Figure 3:**
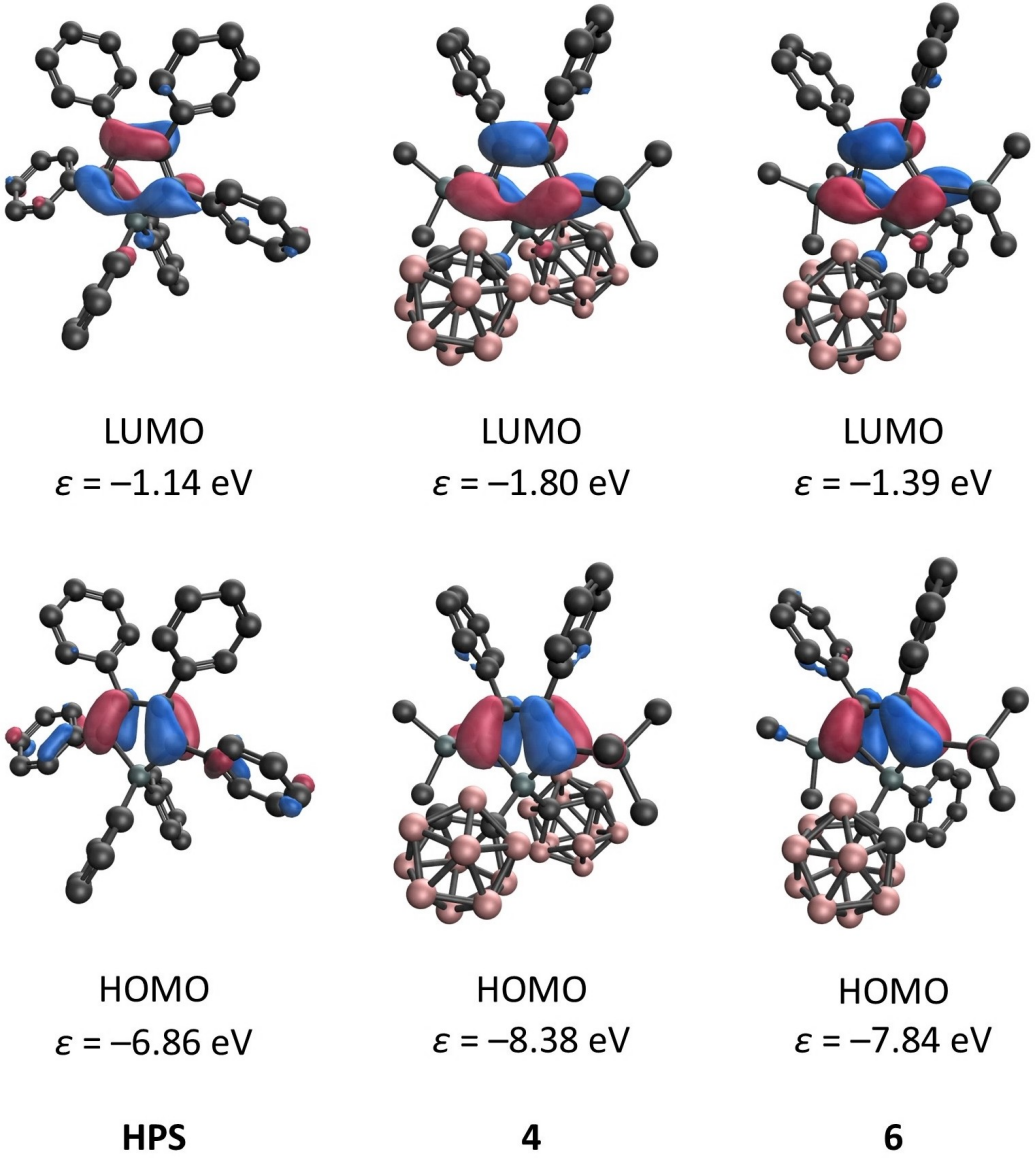
Selected Kohn‐Sham molecular orbitals of 1,1,2,3,4,5‐hexaphenylsilole (**HPS**), **4**, and **6**.

In order to better understand the photophysical properties, DFT calculations were performed at the M06‐2X/6‐31G* level of theory, which is a method that has been suggested for carborane‐based systems recently.^[46]^ Despite the relatively small basis set, this level of theory provides results similar to those obtained with the def2‐TZVP basis set (Table S1 in the SI). Investigating the Kohn‐Sham molecular orbitals, it can be recognised that the shape and the order of the highest occupied molecular orbitals (HOMO−1 and HOMO) and the lowest molecular orbital do not depend on the substitution pattern in the case of all investigated systems (Figure 3 and Figure S5 in the SI). However, the energy levels of the orbitals are shifted upon changing the substituents, especially in 2 and 5 positions on going from the phenyl to the trimethylsilyl group.^[47]^ While the HOMO energy level of **2** is −7.24 eV, it decreases to −8.38 eV in the case of **4**, whereas the respective LUMO energies are −1.50 eV and −1.80 eV. The increased HOMO‐LUMO gap is in complete agreement with the blue‐shifted absorption observed for **4**. It is worth to mention that the energy level of HOMO and HOMO−1 are quite close in the case of **4** with the energy difference being only 0.08 eV. Replacing the phenyl with *o*‐carboranyl substituents at the silicon has a minor impact on the energy levels of the orbitals. It is important to highlight that the carborane moieties do not contribute to these orbitals, which indicates that they act electronically as an innocent unit. Based on the localisation of the frontier orbitals and the TD‐DFT results, the silole unit is responsible for the observed AIE properties, as there is no transition towards the carborane units, which may cause effective quenching.[^10^, ^48^, ^49^] TD‐DFT calculations revealed that the observed absorption maxima are predominantly due to the first excited state (Figure S6), which involves the HOMO‐LUMO and HOMO−1‐LUMO transitions and their combinations (as shown in Table S2–9 in the SI). Indeed, these transitions possess a low charge transfer character, which was in complete agreement with the observed solvent‐independent absorption and emission spectra. TD‐DFT optimisation on S1 states was performed. During the optimisation, no significant structural change can be noticed, which fully agrees with the local excitation mode. Since the carborane moieties do not have contributions to the orbitals in the frontier orbital region, they do not have active electronic contributions to the photophysical properties. In the optimised excited states, we have not observed cage opening in support of any carborane AIE activity (Figure S7–12). Therefore, it can be established that the silole unit is solely responsible for the aggregation‐induced emission property of these compounds. As a next step, we investigated the emission properties of powders of **2**, **4**, **5**, **6**, and **7**. While compound **2** (which seems to be stable in the solid‐state) exhibits a 53 % quantum yield, the replacement of the phenyl substituents at 2,5 positions (compound **4**) leads to a relatively low quantum yield (9 %) even in the solid‐state. Even compound **5**, where one chlorine remained at the silicon atom, strongly emits (*ϕ*=55 %). Very high quantum yields (above 85 %) were measured for compounds **6** and **7**; practically 100 % quantum yield can be achieved in the case of **7**. At first sight, the relatively low quantum yield of compound **4** may be attributed to the presence of SiMe₃ groups. However, this would contrast with the strong emission observed in compounds **6** and **7**, suggesting that despite the reported^[34]^ quenching ability of the trimethylsilyl group. At this point, it is important to highlight that the fluorescence was not quenched by −SiMe_3_ or −GeMe_3_ in the case of the related phosphole derivatives.[^50^, ^51^] Nevertheless, in order to try to understand the difference between the quantum yields of **4** and **6**–**7** in solid‐state, we have further investigated the supramolecular structures for these molecules in their solid structures. It immediately emerges that H⋅⋅⋅H contacts dominate the supramolecular structure; based on the analysis of the Hirshfeld surfaces (see Table S10 in the SI, for this analysis the experimental XRD data were used), the contribution of H⋅⋅⋅H contacts around 90 % in the case of **6** and **7** and 95 % in case of **4**. The rest of the interactions belong to H⋅⋅⋅C(π) coνtacts. In cases **6** and **7**, the phenyl substituent at the silicon atom is positioned between the two phenyl groups located at the 3 and 4 positions of the adjacent molecule, allowing C−H⋅⋅vπ interaction, where the distance of the C−H⋅⋅⋅π is around 2.9 Å. It may hinder the motion of phenyl rings at the 3 and 4 positions of the silole rings, thereby preventing the highly effective quenching mode of siloles. Meanwhile, in the case of **4**, the rings at the 3 and 4 positions can interact with one of the B−H vertices of the adjacent molecule. Due to the hydride character of the H in the BH vertices, the B−H⋅⋅⋅C(π) interaction should be much weaker than the corresponding C−H⋅⋅⋅C(π) interaction (Figure S13 in the SI). Therefore, the motions of the phenyl rings are less restricted, providing a more efficient quenching pathway even in the solid‐state. These results showed that the silyl groups play a less important role during the quenching of the fluorescence of this compound.^[52]^

Finally, we have analysed the fluorescence decay in both aggregated state and solid‐state (Table 2). In the case of the aggregated state of compounds **4**–**7**, a biexponential fit of the fluorescence lifetimes reveals one fast component varying between 2.8 and 8.4 ns and one slower component varying between 18.2 and 35.5 ns. Meanwhile, a monoexponential fit (except **4**) of the fluorescence lifetime can be observed in the solid‐state. This aligns with the general understanding that reduced molecular mobility in the solid‐state leads to more stable fluorescence characteristics and less variation in decay rates. The lifetimes of excited states in the solid‐state increase in the order **4**<**2**<**5**<**6**<**7**, reflecting a similar trend in the quantum yields in the solid‐state of these compounds. This is understandable, as assuming minimal non‐radiative processes in solid‐state, a longer fluorescence lifetime generally leads to a higher quantum yield.

**Table 2:** Time constants for **2**, **4**, **5**, **6**, and **7** in aggregated (THF/water v/v=1/99) and solid‐state. Excitation wavelength: 441 nm (**2**), 280 nm (**4**–**7**).

Compound	Aggregated state		Solid‐state	
	*λ_em_* (nm)	Time constants (ns) and their relative contributions	*λ_em_* (nm)	The time constant (ns) and their relative contribution
**2**	–	–	500	10.0 (100 %)
**4**	440	2.8 (62 %) 24.3 (38 %)	412	1.0 (36 %) 8.5 (64 %)
**5**	483	4.5 (62 %) 18.2 (38 %)	449	15.5 (100 %)
**6**	471	8.4 (26 %) 35.5 (74 %)	456	31.1 (100 %)
**7**	484	6.6 (35 %) 29.1 (65 %)	459	35.1 (100 %)

## Conclusions

A series of air‐stable carborane‐silole conjugates were synthesised and structurally characterised. Their chemical structures were confirmed by X‐ray diffraction as well. Investigating their photophysical properties, revealed that both their absorption and emission spectra show no solvent dependence, indicating a local excitation mode. It was supported by the TD‐DFT calculations, which demonstrated that the absorption bands mainly involve π‐π* transitions of the silole moiety, and the carborane units do not contribute to excitations. The photophysical properties do not change significantly when the *o*‐carboranyl substituents are replaced with the *meta*‐carborane counterparts. The new compounds show no or weak fluorescence in solution, which can be attributed to the silole units in view of experimental and TD‐DFT results. On the other hand, their fluorescence can be recovered; they display strong emission in solid‐state. Systems **6** and **7**, which contain only one carborane unit have the highest quantum yields reaching up to 100 %. The high emission efficiency of these two compounds can be attributed to the unique packing in their solid‐state structures, which restricts the motion of the phenyl groups compared to other investigated derivatives. All of these properties make the new hybrids excellent candidates as luminescent materials.

## Supporting Information Summary

Supporting Information (SI) available from the Wiley Online Library: Description of synthetic procedures, NMR and IR spectra, crystallographic data, DFT and TD‐DFT calculations and xyz coordinates of the optimised structures. SI contains further references.[^53^, ^54^, ^55^, ^56^, ^57^, ^58^, ^59^, ^60^, ^61^] Deposition Numbers 2395439 (for **1**), 2395432 (for **2**), 2401434 (for **4**), 2402095 (for **5**), 2395433 (for **6**), and 2395434 (for **7**) contain the supplementary crystallographic data for this paper. These data are provided free of charge by the joint Cambridge Crystallographic Data Centre and Fachinformationszentrum Karlsruhe Access Structures service.

## Conflict of Interests

The authors declare no conflict of interest.

1

## Supporting information

As a service to our authors and readers, this journal provides supporting information supplied by the authors. Such materials are peer reviewed and may be re‐organized for online delivery, but are not copy‐edited or typeset. Technical support issues arising from supporting information (other than missing files) should be addressed to the authors.

Supporting Information

## Data Availability

The data that support the findings of this study are available in the supplementary material of this article.
