# Supplementary material for: Carborane‐Decorated Siloles with Highly Efficient Solid‐State Emissions – What Drives the Photophysical Properties?
Source: Chemistry. 2025 Jan 28;31(16):e202404462. doi: 10.1002/chem.202404462 (PMC11914931; doi:10.1002/chem.202404462)
Supplement: Supplementary file 1 — Supporting Information [file CHEM-31-e202404462-s001.pdf]

Chemistry–A European Journal

Supporting Information

Carborane-Decorated Siloles with Highly Efficient Solid-State Emissions – What Drives the Photophysical Properties?

Balázs Szathmári, Dóra Hessz, Dániel Zámbó, Clemens Bruhn, Rudolf Pietschnig, Antal Udvardy, Pál Szabó, Tamás Holczbauer, Marcell J. Balogh, and Zsolt Kelemen*

Supporting Information

Carborane-Decorated Siloles with Highly Efficient Solid-State Emissions – What Drives the Photophysical Properties?

Balázs Szathmári,[a] Dóra Hessz,[b] Dániel Zámbó,[c] Clemens Bruhn,[d] Rudolf Pietschnig,[d] Antal Udvardy,[e] Pál Szabó,[f] Tamás Holczbauer,[g] Marcell J. Balogh[a] and Zsolt Kelemen*[a]

[a] Department of Inorganic and Analytical Chemistry, Budapest University of Technology and Economics, Műegyetem rkp. 3, H-1111 Budapest, Hungary
[b] Department of Physical Chemistry and Materials Science and MTA-BME Lendület Quantum Chemistry Research Group, Budapest University of Technology and Economics, Műegyetem rkp. 3, H-1111 Budapest, Hungary
[c] Institute of Technical Physics and Materials Science, HUN-REN Centre for Energy Research, Konkoly-Thege Miklós út 29-33, H-1121 Budapest, Hungary
[d] Institute of Chemistry and CINSat, University of Kassel, Heinrich-Plett-Straße 40, 34132 Kassel, Germany
[e] Department of Physical Chemistry, University of Debrecen, Egyetem tér 1, H-4032 Debrecen, Hungary
[f] Centre for Structural Science, Research Centre for Natural Sciences, Magyar tudósok körútja 2, H-1117 Budapest, Hungary
[g] Chemical Crystallography Research Laboratory and Stereochemistry Research Group, Institute for Organic Chemistry, HUN-REN Research Centre for Natural Sciences, Magyar Tudósok körútja 2A, H-1117 Budapest, Hungary

Table of contents

Investigation of photophysical properties of **2** 2
Investigation of solvent effect on the UV-Vis and emission spectra of **4** 4
Computation details (TD-DFT results) 4
Supramolecular details 13
Fluorescence decay 14
Synthetic procedures 18
NMR spectra 23
IR spectra 36
Simulated UV-Vis spectra 39
Crystal structure and refinement details 43
Calculated structures 72
References 145

Investigation of photophysical properties of 2

Despite compound **2** exhibiting slow decomposition in solution, its photophysical behaviour was investigated. After one day, the ratio of the two peaks changed significantly in the emission spectra. (Figure S2). To detect any degradation during column chromatography, we rotated the plate by 90° and reran it after the first TLC run. As shown in Figure S3, the compound undergoes degradation during the process. We attempted to isolate the impurity; however, its amount does not allow proper characterisation. Measuring the emission spectra of the isolated impurity verified our original assumption: Based on these findings, it can be concluded that the two emission peaks observed in **2** are not due to dual emission.

The stability of compound **2** in solid-state was studied by IR spectroscopy. We recorded the IR spectra of compound 2 and repeated the measurements after 1 day, 1 week, and 1 month, obtaining identical spectra each time. Therefore, based on the IR measurements, no decomposition occurred in the solid-state.

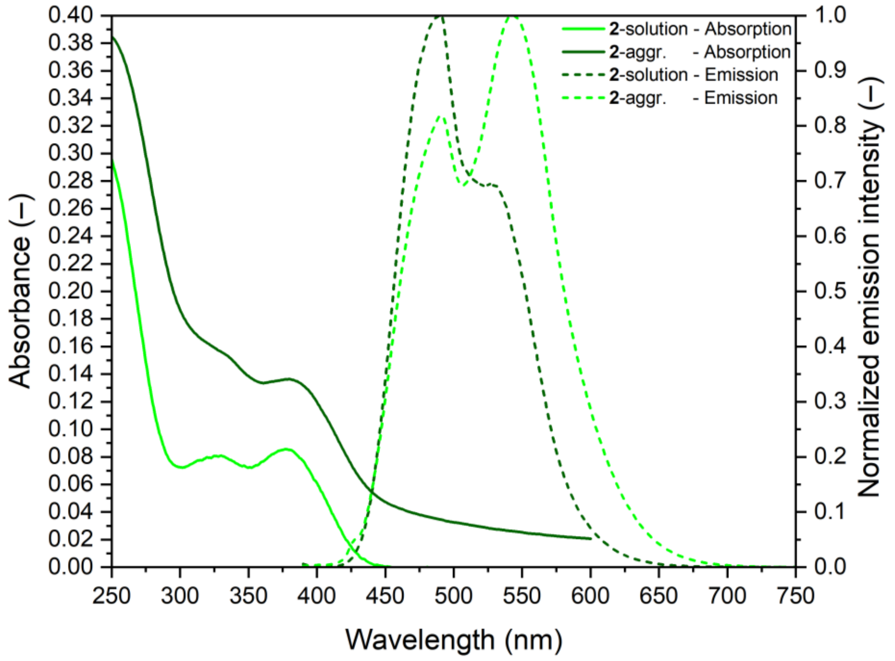

Figure S1 Absorption and emission spectra of **2** in THF solution (~10^{-5} M) and aggregated state

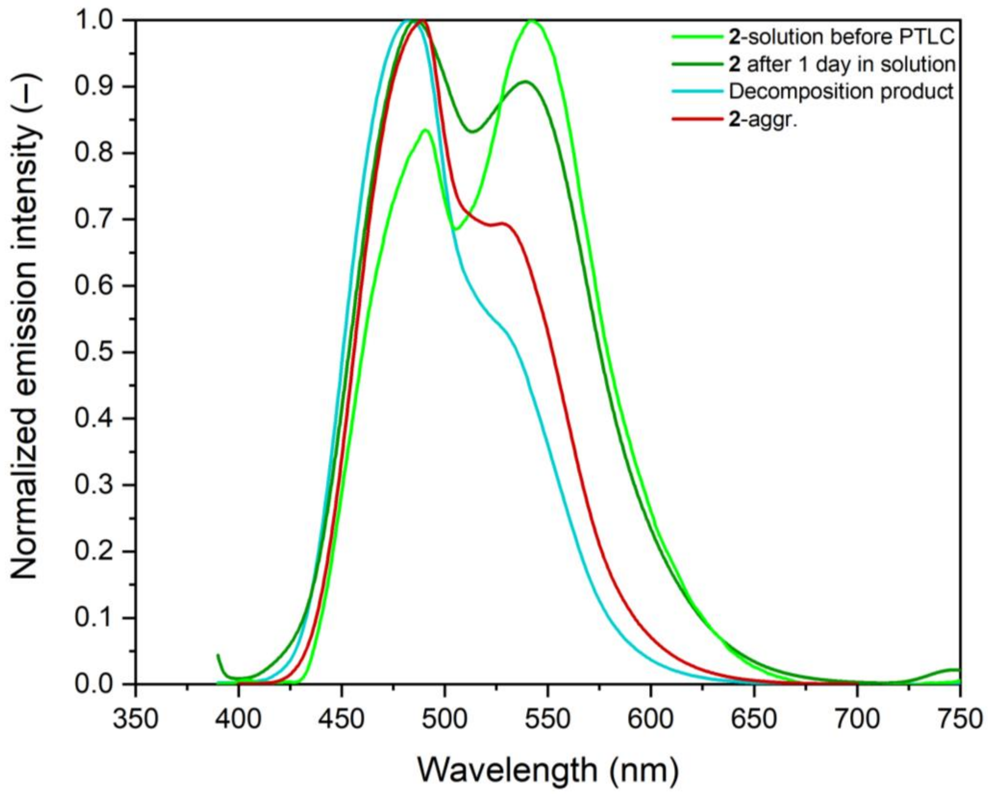

Figure S2 Emission spectra of **2** and its decomposition product

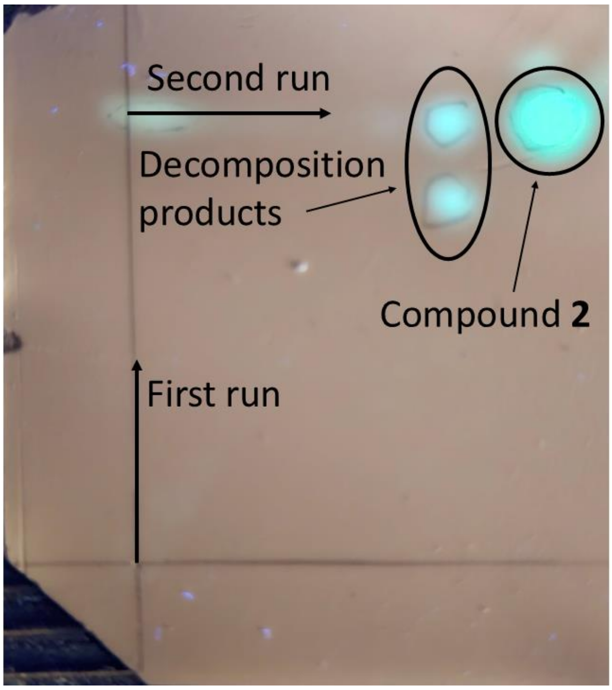

Figure S3 TLC run of **2** for investigating the decomposition

Investigation of solvent effect on the UV-Vis and emission spectra of 4

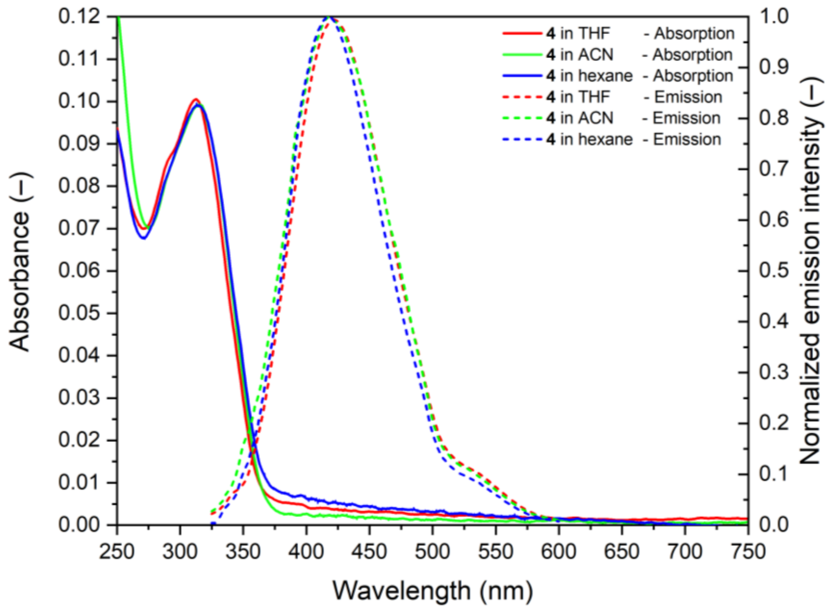

Figure S4 Absorption (solid line) and emission (short-dashed line) spectra of **4** in different solvents (λ_{exc} = 315 nm); THF: tetrahydrofuran, ACN: acetonitrile

Computation details (TD-DFT results)

The Gaussian 16 program package[53] was used for all calculations, while IQmol 2.15.3[54] and GaussSum 3.0[55] were used to visualise the computed structures and orbitals. Geometry optimisations were carried out at the M06-2X/def2-TZVP or M06-2X/6-31g* level of theory. Harmonic vibrational frequency calculations were applied to the fully optimised systems to establish their nature, as characterised by only positive eigenvalues of the Hessian for minima Gibbs free energies were obtained at atmospheric pressure and 298.15 K utilising the calculated harmonic frequencies.

Table S1 Calculated absorption wavelength of the first excited state for **4** in different levels of theory used for optimisation and TD calculation (**bold**: the best match for the measured value)

Level of theory used for optimisation	Level of theory used for TD calculation	First excited state (HOMO→LUMO)		
		Calculated λ_{abs} (nm)	Intensity (f)	%
M06-2X/def2-TZVP	M06-2X/6-31g*	300	0.0551	96
**M06-2X/def2-TZVP**	**M06-2X/def2-TZVP**	**304**	**0.0588**	**96**
M06-2X/def2-TZVP	B3LYP/6-31g*	331	0.0474	98
M06-2X/def2-TZVP	B3LYP/def2-TZVP	332	0.0464	98
M06-2X/def2-TZVP	CAM-B3LYP/6-31g*	300	0.0613	95
M06-2X/def2-TZVP	CAM-B3LYP/def2-TZVP	303	0.0645	95
M06-2X/def2-TZVP	ωB97X-D/6-31g*	300	0.0629	94
M06-2X/def2-TZVP	ωB97X-D/def2-TZVP	303	0.0656	94

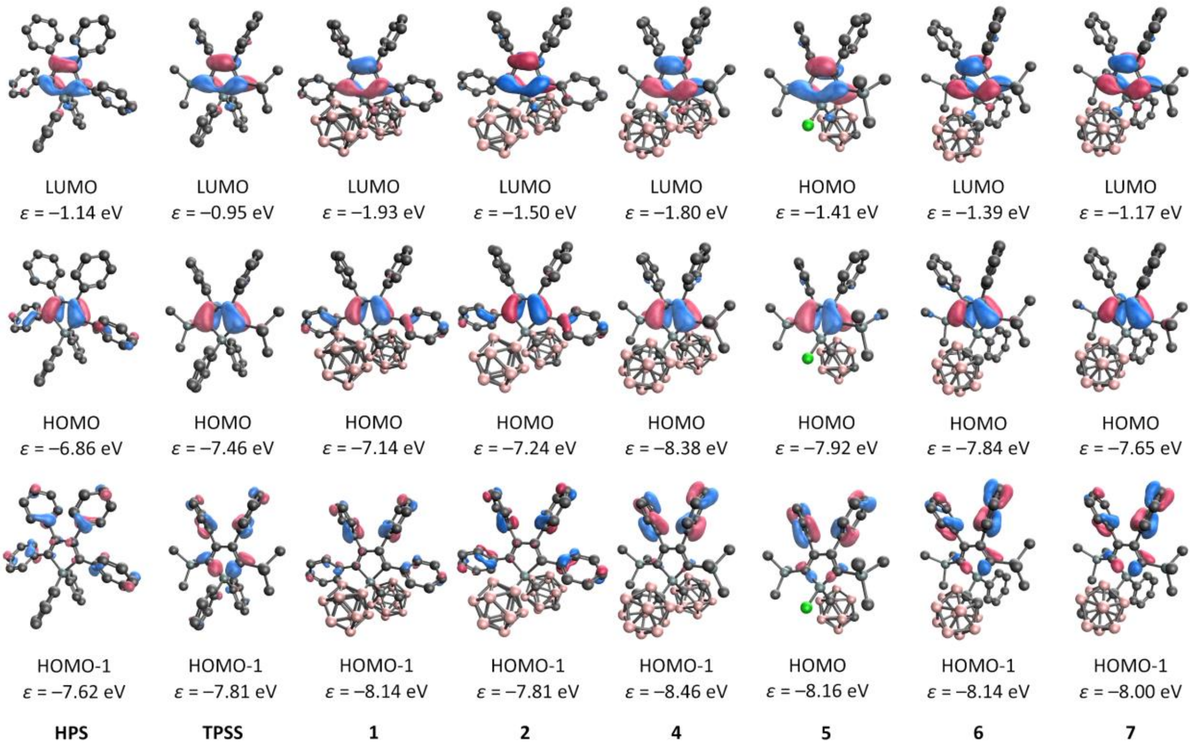

Figure S5 Selected Kohn-Sham molecular orbitals of 1,1,2,3,4,5-Hexaphenylsilole (**HPS**), 1,1,3,4-tetraphenyl-2,5-bis(trimethylsilyl)-silole (**TPSS**), **1**, **2**, **4**, **5**, **6**, and **7**

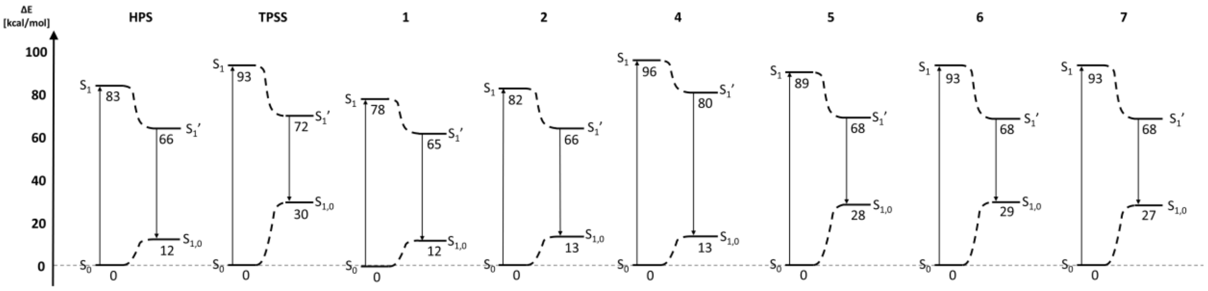

Figure S6 Energy levels of S_1, $S_{1'}$, and $S_{0,1}$ of 1,1,2,3,4,5-Hexaphenylsilole (**HPS**), 1,1,3,4-tetraphenyl-2,5-bis(trimethylsilyl)-silole (**TPSS**), **1**, **2**, **4**, **5**, **6**, and **7**, using the ground state (S_0) for zero-level (M06-2X/6-31g*//M06-2X/6-31g*)

Table S2 TD-DFT results at M06-2X/def2-TZVP of 1,1,2,3,4,5-Hexaphenylsilole (**HPS**) (only contains transitions with contribution above 5% were shown)

Excited state	Calculated λ_{abs} (nm)	Oscillator strength (f)	Transition	Contribution (%)
**1**	355	0.1591	(HOMO→LUMO)	95
**2**	288	0.0262	(HOMO–1→LUMO)	43
			(HOMO–2→LUMO)	42
			(HOMO–15→LUMO)	6
**3**	275	0.2946	(HOMO–1→LUMO)	49
			(HOMO–2→LUMO)	40
**4**	254	0.0974	(HOMO–3→LUMO)	36
			(HOMO→LUMO+2)	21
			(HOMO→LUMO+6)	8
			(HOMO–5→LUMO)	8
**5**	253	0.0098	(HOMO→LUMO+1)	34
			(HOMO–4→LUMO)	21
			(HOMO→LUMO+4)	8
			(HOMO→LUMO+3)	6
			(HOMO→LUMO+8)	5
			(HOMO–1→LUMO+6)	5
**6**	252	0.0683	(HOMO–5→LUMO)	62
			(HOMO–12→LUMO)	6
			(HOMO→LUMO+6)	5

Table S3 TD-DFT results at M06-2X/def2-TZVP of 1,1,3,4-tetraphenyl-2,5-bis(trimethylsilyl)-silole (**TPSS**) (only contains transitions with contribution above 5% were shown)

Excited state	Calculated λ_{abs} (nm)	Oscillator strength (f)	Transition	Contribution (%)
**1**	306	0.0686	(HOMO→LUMO)	95
**2**	301	0.0059	(HOMO–1→LUMO)	71
			(HOMO–10→LUMO)	11
			(HOMO–2→LUMO)	7
**3**	264	0.0253	(HOMO–9→LUMO)	38
			(HOMO–8→LUMO)	26
			(HOMO–6→LUMO)	15
			(HOMO–3→LUMO)	9

Table S4 TD-DFT results at M06-2X/def2-TZVP of **1** (only contains transitions with contribution above 5% were shown)

Excited state	Calculated λ_{abs} (nm)	Oscillator strength (*f*)	Transition	Contribution (%)
**1**	364	0.1187	(HOMO→LUMO)	96
**2**	297	0.2164	(HOMO–1→LUMO)	94
**3**	276	0.0249	(HOMO–2→LUMO)	59
			(HOMO–4→LUMO)	18
			(HOMO–7→LUMO)	8
			(HOMO–21→LUMO)	6
**4**	261	0.0619	(HOMO–3→LUMO)	71
			(HOMO→LUMO+2)	5
			(HOMO–5→LUMO)	5
**5**	258	0.0003	(HOMO–4→LUMO)	51
			(HOMO–2→LUMO)	21
			(HOMO→LUMO+3)	7
**6**	253	0.0924	(HOMO–5→LUMO)	60
			(HOMO–6→LUMO)	16
			(HOMO–2→LUMO+1)	5

Table S5 TD-DFT results at M06-2X/def2-TZVP of **2** (only contains transitions with contribution above 5% were shown)

Excited state	Calculated λ_{abs} (nm)	Oscillator strength (*f*)	Transition	Contribution (%)
**1**	354	0.1067	(HOMO→LUMO)	96
**2**	292	0.1782	(HOMO–1→LUMO)	92
**3**	271	0.0657	(HOMO–2→LUMO)	72
			(HOMO–6→LUMO)	8
**4**	255	0.0641	(HOMO–3→LUMO)	71
			(HOMO–6→LUMO)	8

Table S6 TD-DFT results at M06-2X/def2-TZVP of **4** (only contains transitions with contribution above 5% were shown)

Excited state	Calculated λ_{abs} (nm)	Oscillator strength (*f*)	Transition	Contribution (%)
**1**	303	0.0588	(HOMO→LUMO)	96
**2**	289	0.0395	(HOMO–1→LUMO)	73
			(HOMO–5→LUMO)	16
**3**	272	0.0186	(HOMO–4→LUMO)	35
			(HOMO–2→LUMO)	28
			(HOMO–6→LUMO)	23
**4**	252	0.0533	(HOMO–5→LUMO)	53
			(HOMO–1→LUMO)	24
			(HOMO–18→LUMO)	8
			(HOMO–3→LUMO)	5

Table S7 TD-DFT results at M06-2X/def2-TZVP of **5** (only contains transitions with contribution above 5% were shown)

Excited state	Calculated λ_{abs} (nm)	Oscillator strength (*f*)	Transition	Contribution (%)
**1**	321	0.0445	(HOMO→LUMO)	82
			(HOMO–1→LUMO)	7
**2**	289	0.0478	(HOMO–1→LUMO)	61
			(HOMO–5→LUMO)	15
			(HOMO→LUMO)	14
**3**	268	0.0159	(HOMO–4→LUMO)	34
			(HOMO–6→LUMO)	33
			(HOMO–2→LUMO)	20

Table S8 TD-DFT results at M06-2X/def2-TZVP of **6** (only contains transitions with contribution above 5% were shown)

Excited state	Calculated λ_{abs} (nm)	Oscillator strength (*f*)	Transition	Contribution (%)
**1**	315	0.0506	(HOMO→LUMO)	87
**2**	292	0.0480	(HOMO–1→LUMO)	66
			(HOMO→LUMO)	8
			(HOMO–8→LUMO)	8
			(HOMO–3→LUMO)	6
**3**	268	0.0067	(HOMO–7→LUMO)	42
			(HOMO–2→LUMO)	21
			(HOMO–5→LUMO)	9
			(HOMO–6→LUMO)	8

Table S9 TD-DFT results at M06-2X/def2-TZVP of **7** (only contains transitions with contribution above 5% were shown)

Excited state	Calculated λ_{abs} (nm)	Oscillator strength (*f*)	Transition	Contribution (%)
**1**	316	0.0482	(HOMO→LUMO)	83
			(HOMO–1→LUMO)	7
**2**	290	0.0463	(HOMO–1→LUMO)	63
			(HOMO→LUMO)	12
			(HOMO–8→LUMO)	6
			(HOMO–3→LUMO)	5
**3**	267	0.0090	(HOMO–7→LUMO)	38
			(HOMO–2→LUMO)	17
			(HOMO–5→LUMO)	13
			(HOMO–4→LUMO)	9
			(HOMO–8→LUMO)	9

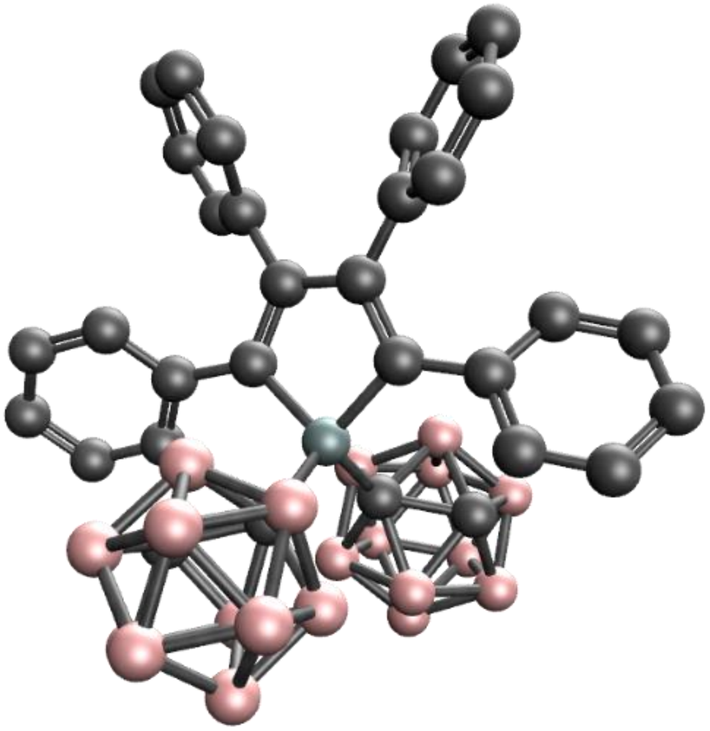

Figure S7 Optimized first excited state of **1** (M06-2X/6-31g*). Hydrogen atoms are omitted for clarity.

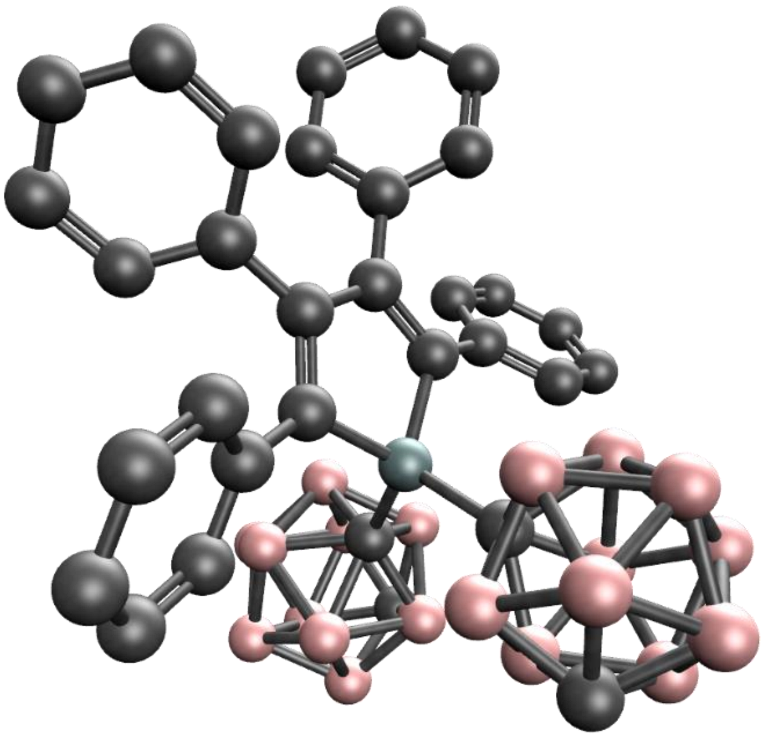

Figure S8 Optimized first excited state of **2** (M06-2X/6-31g*). Hydrogen atoms are omitted for clarity.

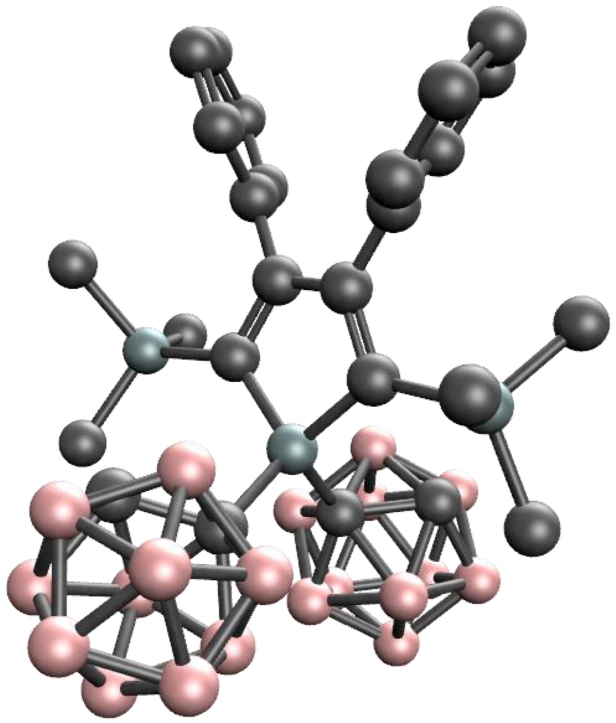

Figure S9 Optimized first excited state of **4** (M06-2X/6-31g*). Hydrogen atoms are omitted for clarity

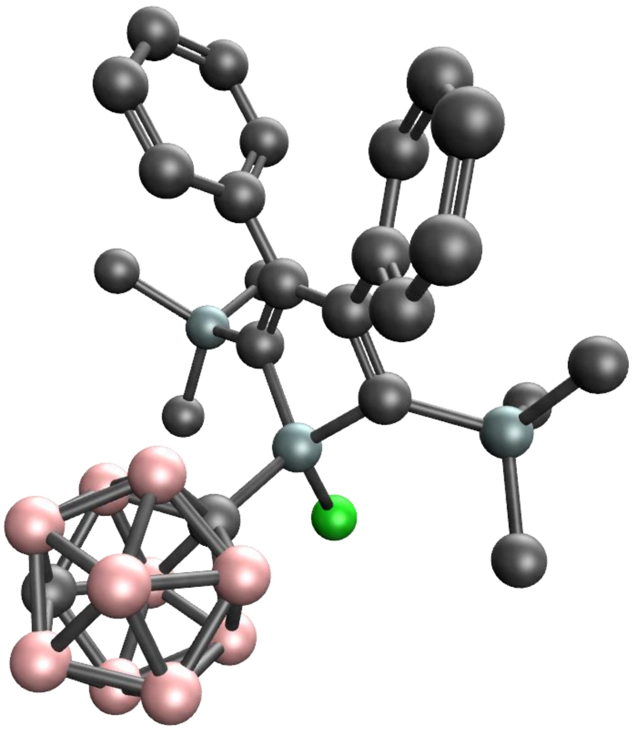

Figure S10 Optimized first excited state of **5** (M06-2X/6-31g*). Hydrogen atoms are omitted for clarity.

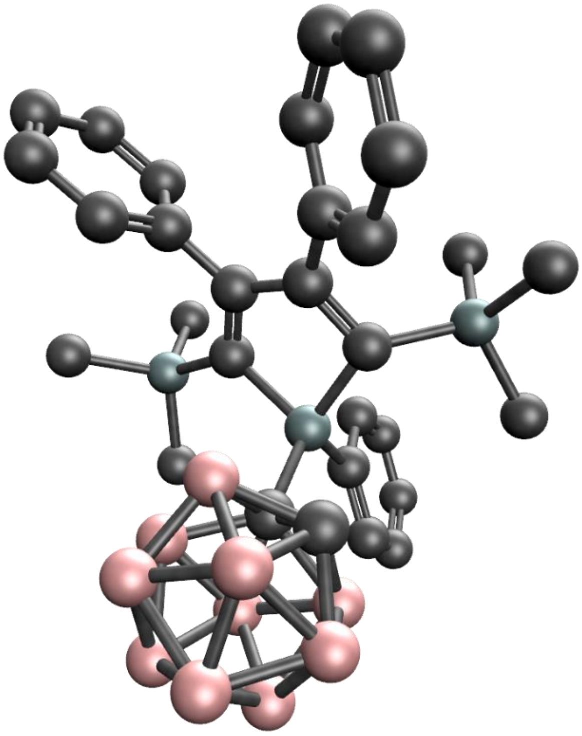

Figure S11 Optimized first excited state of **6** (M06-2X/6-31g*). Hydrogen atoms are omitted for clarity.

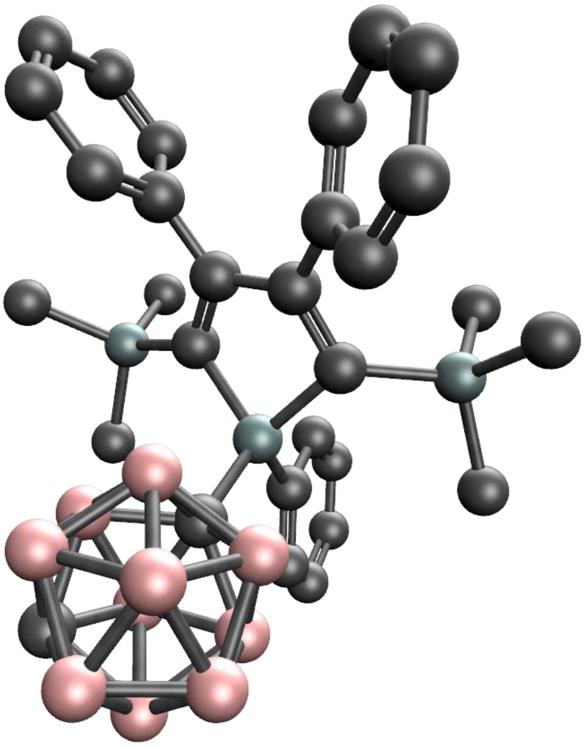

Figure S12 Optimized first excited state of **7** (M06-2X/6-31g*). Hydrogen atoms are omitted for clarity.

Supramolecular details

Table S10 Relative contributions of various intermolecular contacts to the Hirshfeld surface of **4**, **5**, **6**, and **7** (CrystalExplorer 21.5[56] was used for calculations)

Compounds\Interactions	C···H (%)	B···H (%)	H···H (%)	Cl···H (%)	Cl···C (%)	Si···H (%)	QY (%)
**4**	5.4	0	94.6	–	–	0	9
**5**	7.7	0	85.8	6.3	0.1	<0.1	55
**6**	11.0	0	89.0	–	–	0	86
**7**	10.4	0	89.7	–	–	0	100

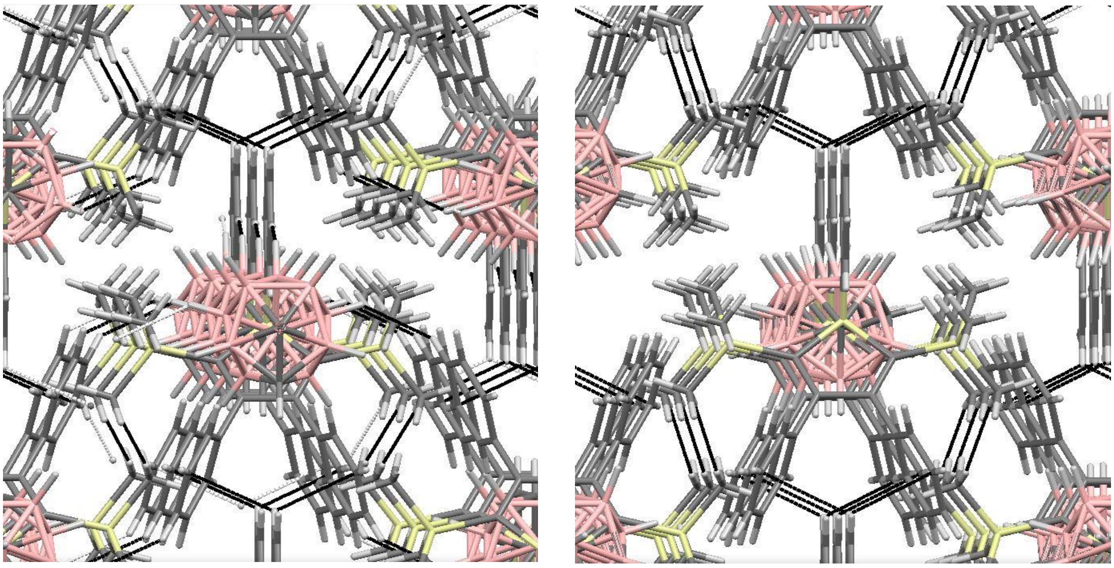

Figure S13 Supramolecular structure of **6** (left) and **7** (right), black lines representing short contacts (C-H···C between the phenyl rings or C-H···H-C between TMS groups)

Fluorescence decay

The data in Table 2 were obtained by applying an exponential function to the recorded decay curves (after subtracting the instrument response function) using the implemented fitting program (the fit optimized so that the χ^2 value approached 1.000 as closely as possible). Based on this approach, we identified two time constants in the aggregated state and one time constant in the solid state for each compound measured, with χ^2 values close to 1 in all cases. Assuming structural similarities between the aggregated and solid-states, it is conceivable that the time constants might also be similar. Accordingly, we performed an alternative fit for the aggregated state data, where we fixed the time constants derived from the crystal state. This approach resulted in a slight decrease in fit accuracy (a small increase in χ^2), but the fit quality remained satisfactory for **4**, **5**, and **7**. For **6**, however, the χ^2 value increased significantly, leading to a substantial deviation in the fit. These results indicate that the structural properties of the aggregate state of **4**, **5** and **7** are close to the crystalline phase, whereas this is not the case for **6**.

Table S11 Time constants for **2**, **4**, **5**, and **7** in aggregated (THF/water v/v = 1/99) and solid-state obtained by an alternative fitting Excitation wavelength: 441 nm (**2**), 280 nm (**4**-**7**)

**Compound**	**Aggregated state**		**Solid-state**	
	**λ_{em} (nm)**	**Time constants (ns) and their relative contributions**	**λ_{em} (nm)**	**The time constant (ns) and their relative contribution**
**2**	–	–	500	10.0 (100%)
**4**	440	1.0 (27%)<br>8.5 (40%)<br>33.8 (33%)	412	1.0 (36%)<br>8.5 (64%)
**5**	483	2.7 (55%)<br>15.5 (45%)	449	15.5 (100%)
**7**	484	10.2 (51%)<br>35.1 (49%)	459	35.1 (100%)

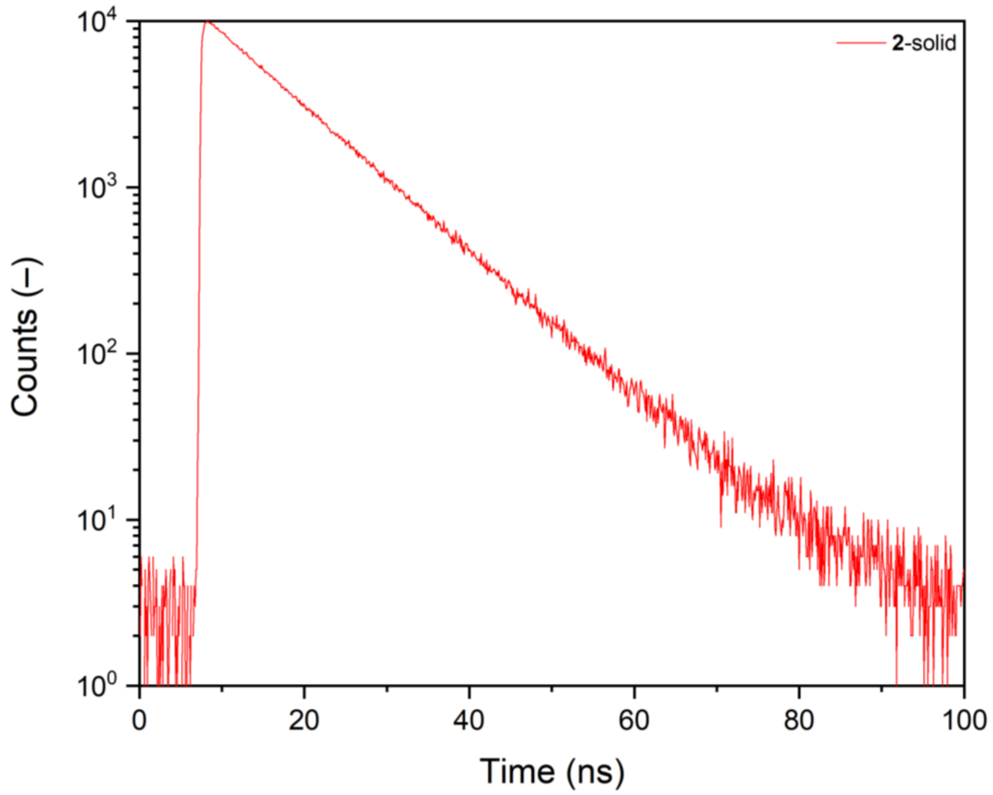

Figure S14 Fluorescence lifetime decay curve of **2** in solid-state

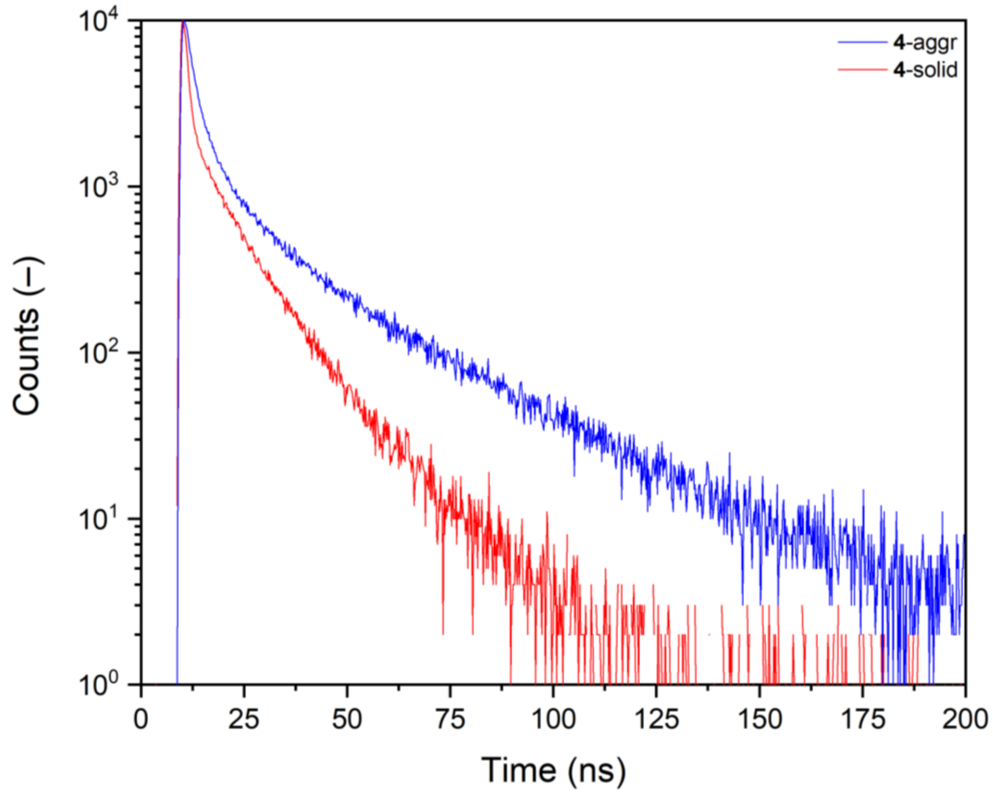

Figure S15 Fluorescence lifetime decay curves of **4** in aggregated state and solid-state

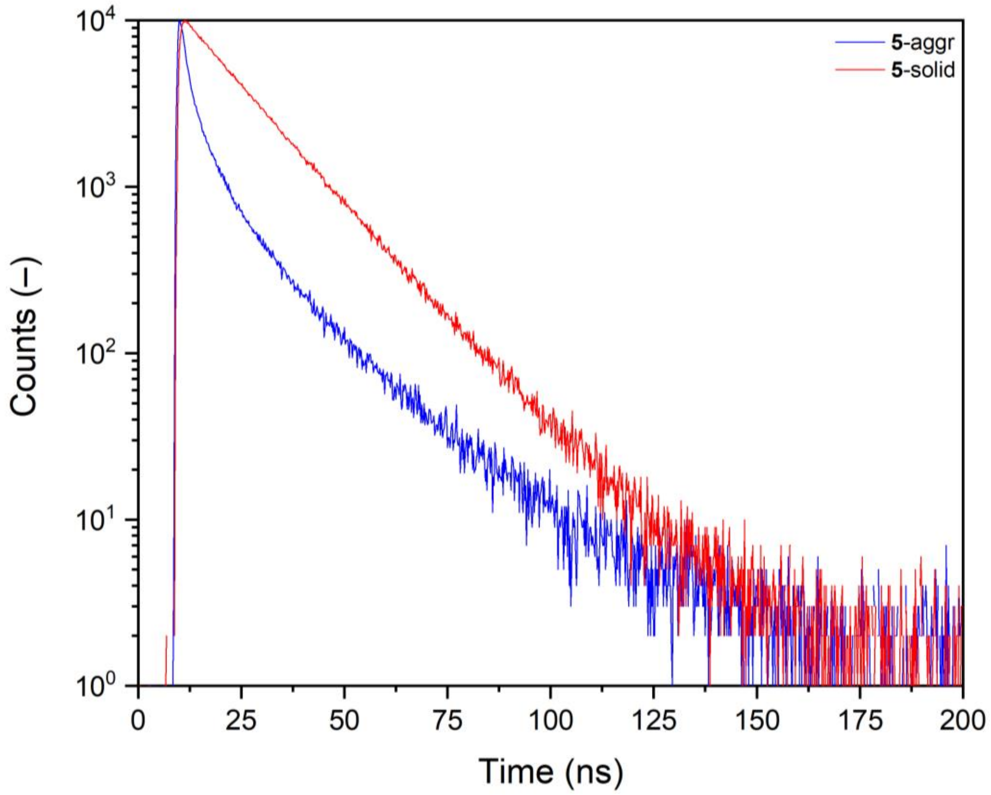

Figure S16 Fluorescence lifetime decay curves of **5** in aggregated state and solid-state

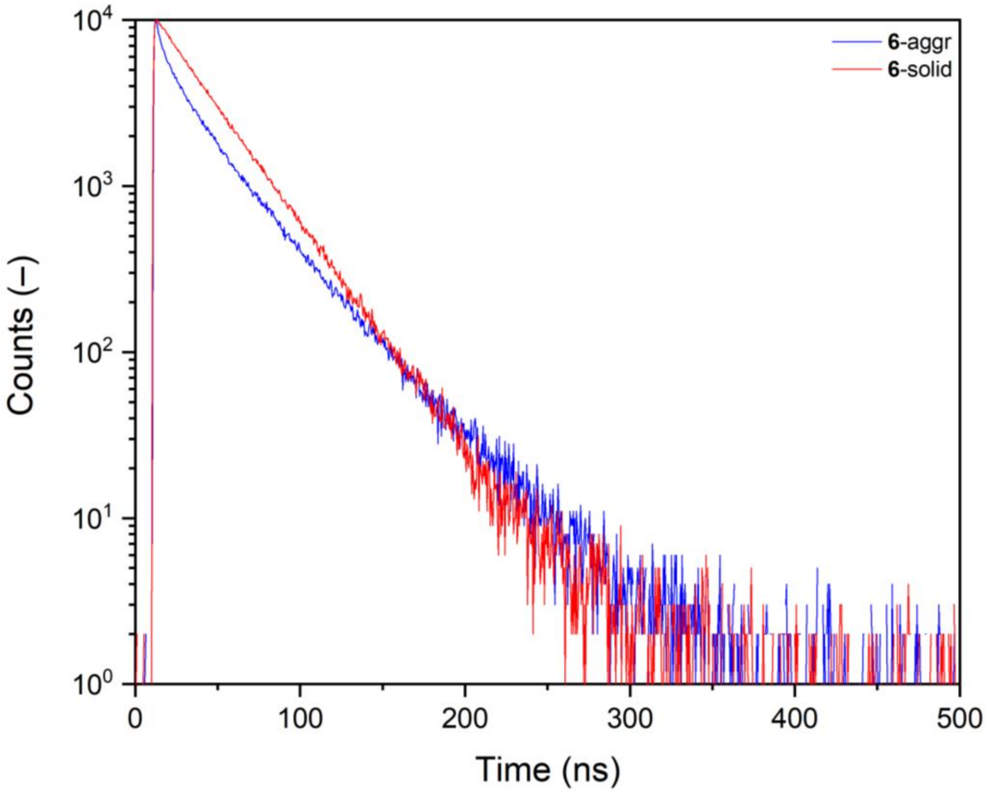

Figure S17 Fluorescence lifetime decay curves of **6** in aggregated state and solid-state

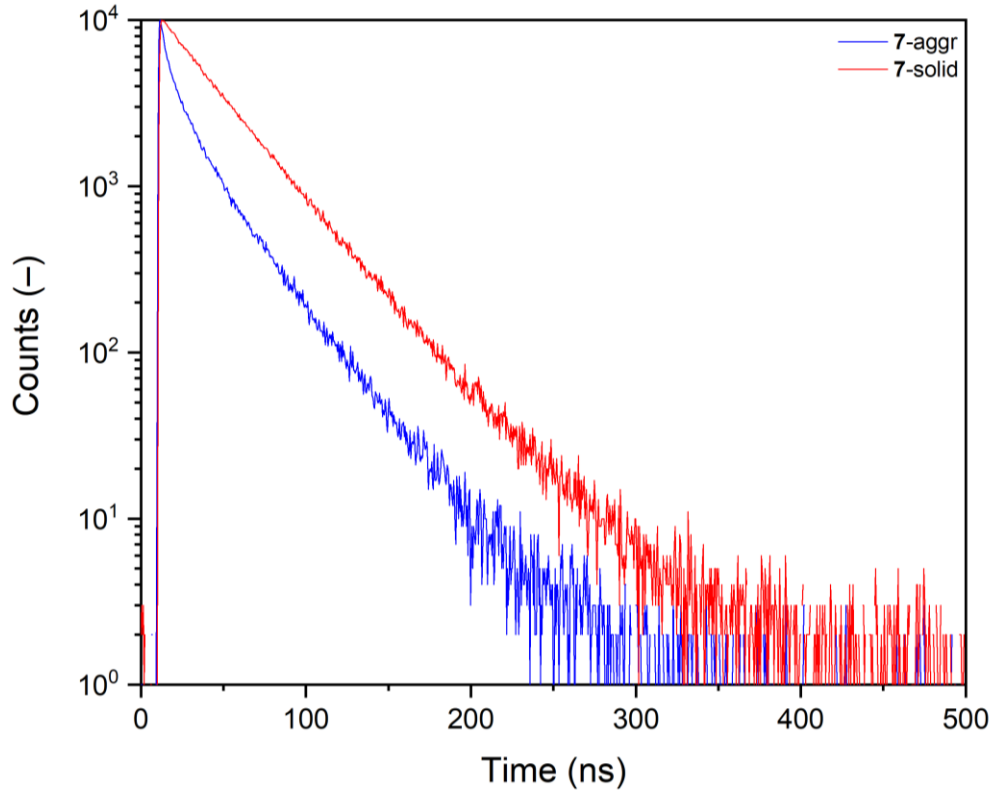

Figure S18 Fluorescence lifetime decay curves of **7** in aggregated state and solid-state

Synthetic procedures

All manipulations (unless otherwise stated) were performed under a dry nitrogen atmosphere with the exclusion of air and moisture using standard Schlenk techniques. The solvent for the reactions (THF) was dried using standard procedures (over sodium/benzophenone) and freshly distilled before use. HPLC-grade solvents were used for the photophysical measurements (THF, water, hexane, acetonitrile); for crystallisation, freshly distilled technical-grade solvents were used (DCM, EtOAc, hexane, acetone). Carboranes were purchased from Zhengzhou Yuanli Biological Technology Co. Ltd and used as received. The starting chloro-siloles and dichloro-siloles were prepared according to Tamao's produce,[35] while in the case of 1,1-dichloro-2,3,4,5-tetraphenylsilole, we have reacted 1,4-dilithio 1,2,3,4-tetraphenyl-butadiene and $SiCl_4$ on –80°C (modified from the recipe found in the literature).[36]

The ^{1}H, ^{13}C, and ^{11}B, ^{29}Si NMR spectra were recorded on Bruker DRX 500 and Jeol JNM-ECZL500 spectrometers, using the deuterated solvent ($CDCl_3$) as an internal lock. ^{11}B NMR and $^{11}B\{^{1}H\}$ NMR chemical shifts were referenced to the external $BF_3{\cdot}OEt_2$, $^{29}Si\{^{1}H\}$ NMR chemical shifts were referenced to the external TMS (tetramethoxysilane). In the case of ^{11}B, $^{11}B\{^{1}H\}$ and $^{29}Si\{^{1}H\}$ spectra, the baseline was corrected by the built-in *apbk* method of the Bruker TopSpin 4.4.0. program.

IR measurements were performed on a Perkin Elmer Spectrum Two FT-IR spectrometer using the attenuated total reflection (ATR) technique on powdered samples. UV-Vis measurements were performed on a UNICAM UV4-100 UV/Vis spectrophotometer. Fluorescence measurements for the solution state were performed on JASCO-8350 spectrofluorometer. The fluorescence spectra of aggregated state compounds and the fluorescence decay curves were measured with an FS5 Edinburgh Instruments combined steady state and fluorescence lifetime spectrometer. Two pulsed light sources – an EPLED-UV 280 (emitting at 281 nm, pulse width ~ 900 ps) and an EPL-450 (emitting at 441 nm, pulse width ~ 90 ps) were used when the decay curves were measured. In the case of the diode (EPLED-UV 280), a 320 nm longpass glass filter was placed in the emission light path, while for the laser (EPL-450), a 495 nm longpass glass filter was used. Absolute quantum yield measurements were carried out with an Edinburgh FS5 (Edinburgh Instruments, UK) spectrofluorometer by using an integrating sphere (SC-30, direct illumination, PTFE sample holder with quartz coverslip) for solid samples. Reference spectra for quantum yield calculations were taken by measuring the empty solid sample holder. The determination of the quantum yield was performed using the Fluoracle

software. According to our experience, these measurements could have a 5% error, which is in agreement with the literature data. High-resolution mass spectrometric measurements were performed using a Sciex5600+Q-TOF mass spectrometer in positive electrospray ionization (ESI) mode or in some cases atmospheric-pressure chemical ionization (APCI) mode.

Synthesis of **1**

In inert atmosphere, 500 mg (3.47 mmol) of *o*-carborane was dissolved in 50 mL THF, and 2.5 mL (3.47 mmol) BuLi in hexane solution was added dropwise for 1h at 0-5°C. The mixture was stirred for 1h at 0-5°C and then let warm to room temperature, and a white precipitate appeared in the mixture. Then 528 mg (1.16 mmol) 1,1-dichloro-2,3,4,5-tetraphenylsilole dissolved in 50 mL THF was added to the mixture dropwise for 2h, using intensive stirring. The mixture was stirred overnight. The THF was removed by evaporation to get the raw product, and DCM was used to extract the product from the remaining solid. The LiCl was filtrated, so a green raw product was obtained. The pure product was obtained by column chromatography (EtOAc-hexane 1:15) and by recrystallisation from DCM at room temperature as green crystals. Yield: few crystals.

Synthesis of **2** (and **3**)

In inert atmosphere, 500 mg (3.47 mmol) *m*-carborane was dissolved in 50 mL THF, and 2.5 mL (3.47 mmol) BuLi in hexane solution was added dropwise for 1h at room temperature and stirred for 1h. Then 528 mg (1.16 mmol) 1,1-dichloro-2,3,4,5-tetraphenylsilole dissolved in 50 mL THF was added to the mixture dropwise for 2h, using intensive stirring. The mixture was stirred overnight. The THF was removed by evaporation to get the raw product, and DCM was used to extract the product from the remaining solid. The LiCl was filtrated, so a green raw product was obtained. The pure product was obtained by column chromatography (EtOAc-hexane 1:15) and by recrystallisation from acetone at room temperature as green crystals.

2: Yield: 178 mg (23%); Melting point: 262-263 °C. ^{1}H NMR (500.13 MHz, $CDCl_3$): δ = 2.85 (s, 2H, $CH_{carborane}$), 6.70–6.72 (m, 4H, PhH), 6.94–7.02 (m, 6H, PhH), 7.09–7.10 (m, 6H, PhH), 7.20–7.21 (m, 4H, PhH). ^{13}C{^{1}H} NMR (125.77 MHz, $CDCl_3$): δ = 57.3 (Si-$C_{carborane}$), 59.7 ($CH_{carborane}$), 126.7, 127.1, 127.55, 127.57, 130.4, 132.1, 136.8, 137.9, 138.1, 159.5. ^{11}B{^{1}H} (160.46 MHz, $CDCl_3$): δ = –15.0, –11.7, –10.4, –9.3, –2.8. ^{11}B (160.46 MHz, $CDCl_3$): δ = –14.3, –10.9, –9.6, –

8.7, –2.7. $^{29}Si\{^1H\}$ NMR (99.37 MHz, $CDCl_3$): δ = –1.1 (Si_{ring}). HRMS (ESI): *m/z* $[M+H]^+$ calcd. for $C_{32}H_{43}B_{20}Si^+$ 671.5155, found 671.5124.

3: ^1H NMR (500.13 MHz, $CDCl_3$): δ = 2.80 (s, 1H, $CH_{carborane}$), 6.68–6.69 (d, 4H, PhH), 6.93–6.96 (t, 4H, PhH), 6.99–7.07 (m, 8H, PhH), 7.12–7.14 (m, 4H, PhH).

Synthesis of **4**

In inert atmosphere, 500 mg (3.47 mmol) of *o*-carborane was dissolved in 50 mL THF, and 2.5 mL (3.47 mmol) BuLi in hexane solution was added dropwise for 1h at 0-5°C. The mixture was stirred for 1h at 0-5°C and then let warm to room temperature, and a white precipitate appeared in the mixture. Then 519 mg (1.16 mmol) 1,1-dichloro-2,5-bis(trimethylsilyl)-3,4-diphenylsilole dissolved in 50 mL THF was added to the mixture dropwise for 2h, using intensive stirring. The mixture was stirred overnight. The THF was removed by evaporation to get the raw product, and DCM was used to extract the product from the remaining solid. The LiCl was filtrated, so a pale-yellow raw product was obtained. The pure product was obtained by column chromatography (EtOAc-hexane 1:9) and by recrystallisation from THF at room temperature as white crystals. Yield: 247 mg (32%).

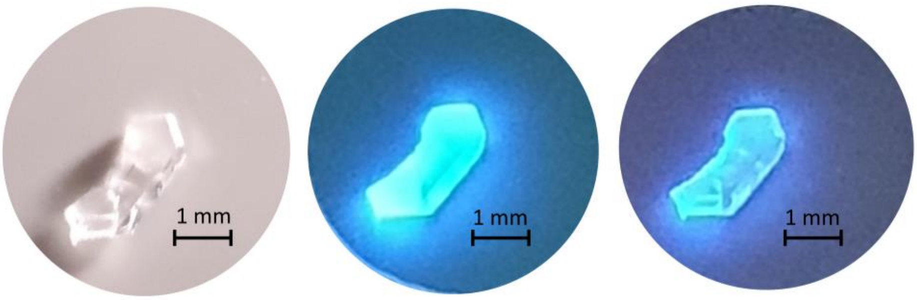

Figure S19. Crystalline material in visible light (right), 254 nm UV light (middle) and 366 nm UV light (left)

Melting point: decomposes around 205-210 °C. ^1H NMR (500.13 MHz, $CDCl_3$): δ = –0.02 (s, 18H, $SiMe_3$), 3.66 (s, 2H, $CH_{carborane}$), 6.74–6.76 (m, 4H, PhH), 7.04–7.05 (m, 6H, PhH). $^{13}C\{^1H\}$ NMR (125.77 MHz, $CDCl_3$): δ = 2.8 ($SiMe_3$), 61.3 ($Si\text{-}C_{carborane}$), 63.0 ($CH_{carborane}$), 127.2, 127.5, 129.0, 136.6, 139.8, 177.5. $^{11}B\{^1H\}$ (160.46 MHz, $CDCl_3$): δ = –12.9, –12.4, –10.1, –7.0, –1.1, 2.2. ^{11}B (160.46 MHz, $CDCl_3$): δ = –13.7, –12.5, –11.4, –10.5, –9.4, –7.4, –6.5, –1.7, –0.7, 1.6, 2.5. $^{29}Si\{^1H\}$ NMR (99.37 MHz, $CDCl_3$): δ = –8.2 ($SiMe_3$), 19.5 (Si_{ring}).

Synthesis of **5**

In inert atmosphere, 500 mg (3.47 mmol) *m*-carborane was dissolved in 50 mL THF, and 2.5 mL (3.47 mmol) BuLi in hexane solution was added dropwise for 1h at room temperature and stirred for 1h. Then 519 mg (1.16 mmol) 1,1-dichloro-2,5-bis(trimethylsilyl)-3,4-diphenylsilole dissolved in 50 mL THF was added to the mixture dropwise for 2h, using intensive stirring. The mixture was stirred overnight. The THF was removed by evaporation to get the raw product, and DCM was used to extract the product from the remaining solid. The LiCl was filtrated, so a yellow raw product was obtained. The pure product was obtained by column chromatography (EtOAc-hexane 1:9) and by recrystallisation from THF at room temperature as white crystals. Yield: 178 mg (28%).

Melting point: 169-170 °C. ^{1}H NMR (500.13 MHz, $CDCl_3$): δ –0.03 (s, 18H, $SiMe_3$), 3.10 (s, 1H, $CH_{carborane}$), 6.82–6.86 (m, 4H, PhH), 7.02–7.08 (m, 6H, PhH). $^{13}C\{^{1}H\}$ NMR (125.77 MHz, $CDCl_3$): δ = 1.1 ($SiMe_3$), 57.9 ($CH_{carborane}$), 62.4 (Si-$C_{carborane}$), 127.1, 127.3, 128.8, 138.0, 140.4, 173.0. $^{11}B\{^{1}H\}$ (160.46 MHz, $CDCl_3$): δ = –14.7, –11.7, –10.4, –11.7, –10.4, –8.9, –3.8. ^{11}B (160.46 MHz, $CDCl_3$): δ = –15.3, –14.2, –12.2, –11.2, –9.4, –8.5, –4.2, –3.3. $^{29}Si\{^{1}H\}$ NMR (99.37 MHz, $CDCl_3$): δ = –8.3 ($SiMe_3$), 19.2 (Si_{ring}). HRMS (APCI): *m/z* $[M]^+$ calcd. for $C_{24}H_{39}B_{10}ClSi_3^+$ 555.3078, found 555.3022.

Synthesis of **6**

In inert atmosphere, 250 mg (1.73 mmol) *o*-carborane was dissolved in 50 mL THF, and 2.5 mL (3.47 mmol) BuLi in hexane solution was added dropwise for 1h at 0-5°C. The mixture was stirred for 1h at 0-5°C and then let warm to room temperature, and a white precipitate appeared in the mixture. Then 774 mg (1.58 mmol) 1-chloro-2,5-bis(trimethylsilyl)-1,3,4-triphenylsilole dissolved in 50 mL THF was added to the mixture dropwise for 2h, using intensive stirring. The mixture was stirred overnight. The THF was removed by evaporation to get the raw product, and DCM was used to extract the product from the remaining solid. The LiCl was filtrated, so a yellow raw product was obtained. The pure product was obtained by column chromatography (EtOAc-hexane 1:7) and by recrystallisation from THF at room temperature as white crystals. Yield: 434 mg (46%).

Melting point: decomposes around 215-220 °C. ^{1}H NMR (500.13 MHz, $CDCl_3$): δ = –0.27 (s, 18H, $SiMe_3$), 3.64 (s, 1H, $CH_{carborane}$), 6.90–6.92 (m, 4H, PhH), 7.05–7.11 (m, 6H, PhH), 7.44–

7.52 (m, 3H, PhH), 7.81–7.82 (m, 2H, PhH). $^{13}C\{^{1}H\}$ NMR (125.77 MHz, $CDCl_3$): δ = 1.3 ($SiMe_3$), 63.8 (Si-$C_{carborane}$), 63.4 ($CH_{carborane}$), 127.1, 127.5, 128.6, 128.8, 129.0, 131.2, 134.7, 141.1, 141.6, 175.5. $^{11}B\{^{1}H\}$ (160.46 MHz, $CDCl_3$): δ = –12.4, –10.0, –7.2, –1.2, 1. ^{11}B (160.46 MHz, $CDCl_3$): δ = –12.8, –10.5, –9.6, –7.8, –6.9, –1.8, –0.9, 0.5, 1.3. $^{29}Si\{^{1}H\}$ NMR (99.37 MHz, $CDCl_3$): δ = –8.7 ($SiMe_3$), 10.9 (Si_{ring}). HRMS (ESI): *m/z* $[M+H]^+$ calcd. for $C_{30}H_{45}B_{10}Si_3^+$ 598.3818, found 598.3807.

Synthesis of **7**

In inert atmosphere, 250 mg (1.73 mmol) *m*-carborane was dissolved in 50 mL THF, and 2.5 mL (3.47 mmol) BuLi in hexane solution was added dropwise for 1h at room temperature and stirred for 1h. Then 774 mg (1.58 mmol) 1-chloro-2,5-bis(trimethylsilyl)-1,3,4-triphenylsilole dissolved in 50 mL THF was added to the mixture dropwise for 2h, using intensive stirring. The mixture was stirred overnight. The THF was removed by evaporation to get the raw product, and DCM was used to extract the product from the remaining solid. The LiCl was filtrated, so a yellow raw product was obtained. The pure product was obtained by column chromatography (EtOAc-hexane 1:7) and by recrystallisation from THF at room temperature as white crystals. Yield: 415 mg (44%).

Melting point: decomposes around 210-220 °C. ^{1}H NMR (500.13 MHz, $CDCl_3$): δ = –0.28 (s, 18H, $SiMe_3$), 3.13 (s, 1H, $CH_{carborane}$), 6.93 (brs, 4H, PhH), 7.02–7.09 (m, 6H, PhH), 7.39–7.45 (m, 3H, PhH), 7.75–7.76 (m, 2H, PhH). $^{13}C\{^{1}H\}$ NMR (125.77 MHz, $CDCl_3$): δ = 1.4 ($SiMe_3$), 57.8 ($CH_{carborane}$), 61.0 (Si-$C_{carborane}$), 126.7, 127.3, 128.3, 129.0, 130.4, 130.5, 134.7, 141.6, 143.4, 173.7. $^{11}B\{^{1}H\}$ (160.46 MHz, $CDCl_3$): δ = –14.4, –11.9, –10.1, –9.0, –3.3. ^{11}B (160.46 MHz, $CDCl_3$): δ = –14.8, –13.8, –12.4, –11.3, –9.5, –8.5, –3.6, –2.9. $^{29}Si\{^{1}H\}$ NMR (99.37 MHz, $CDCl_3$): δ = –8.9 ($SiMe_3$), 10.3 (Si_{ring}). HRMS (APCI): *m/z* $[M]^+$ calcd. for $C_{30}H_{44}B_{10}Si_3^+$ 597.3740, found 597.3742.

NMR spectra

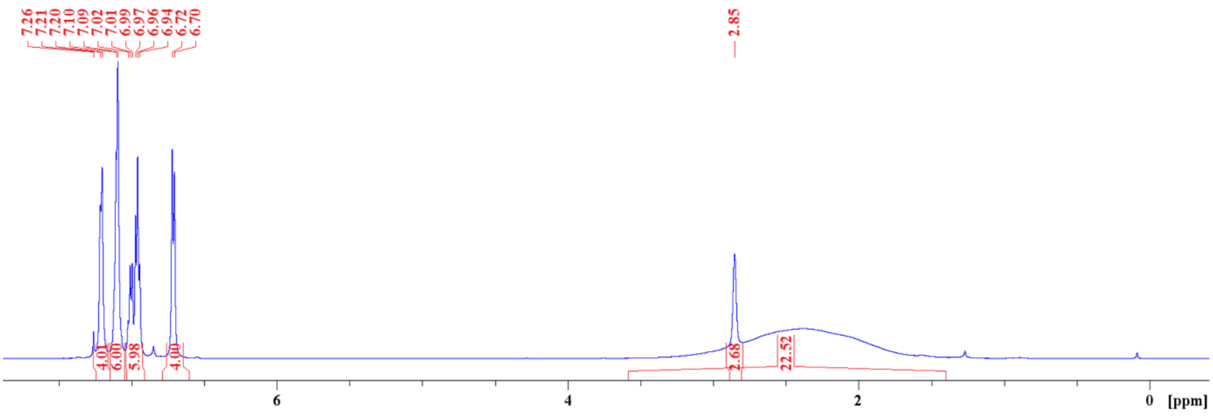

Figure S20 ^{1}H NMR spectrum of **2** in $CDCl_3$

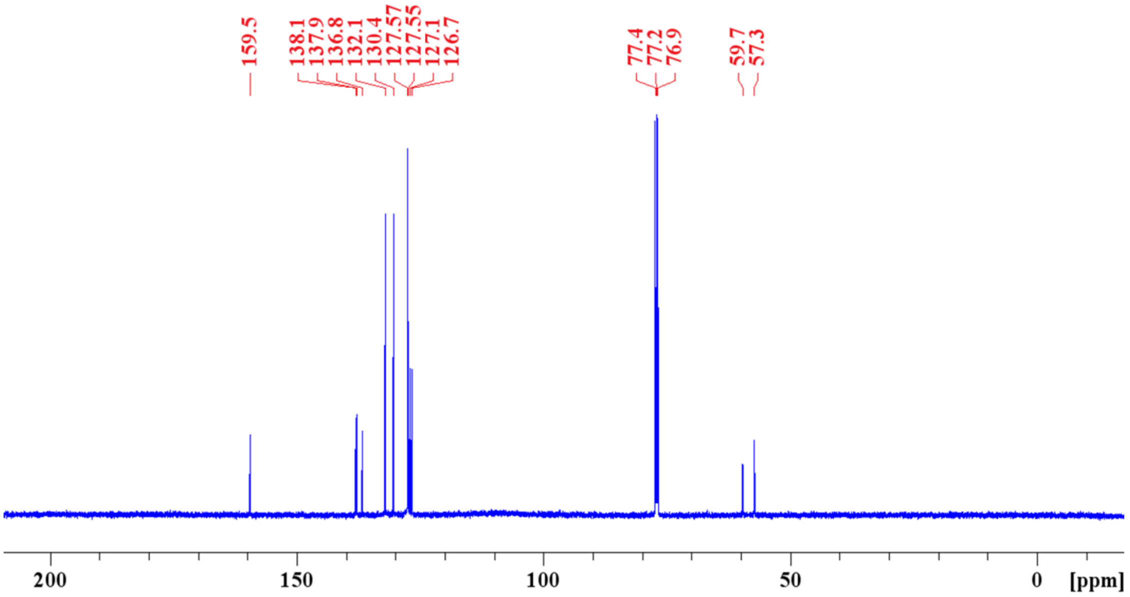

Figure S21 ^{13}C NMR spectrum of **2** in $CDCl_3$

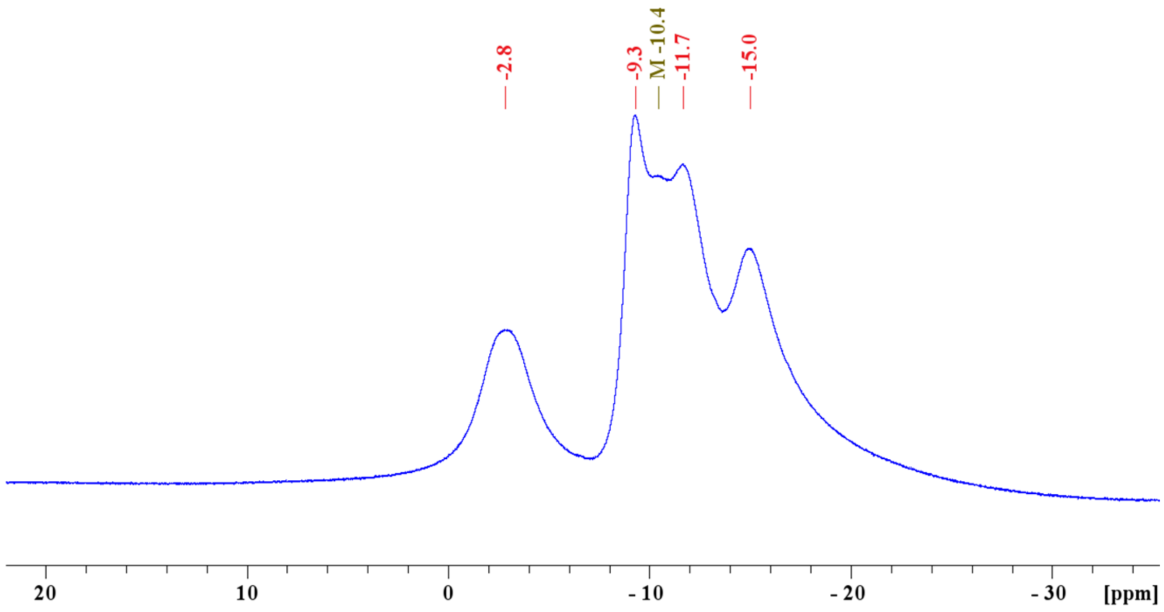

Figure S22 $^{11}B\{^{1}H\}$ NMR spectrum of **2** in $CDCl_3$

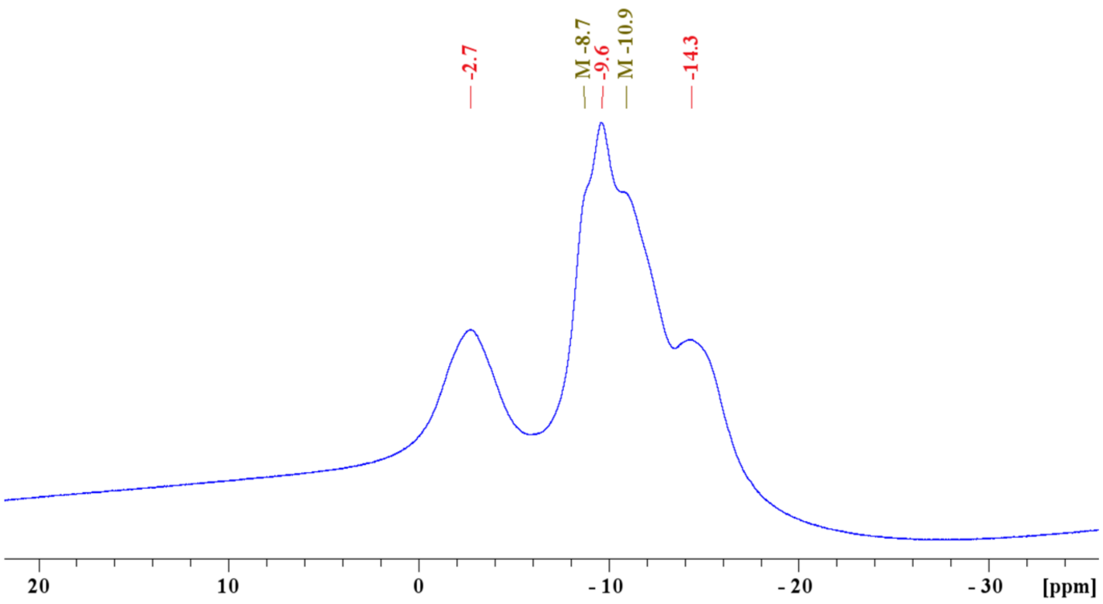

Figure S23 ^{11}B NMR spectrum of **2** in $CDCl_3$

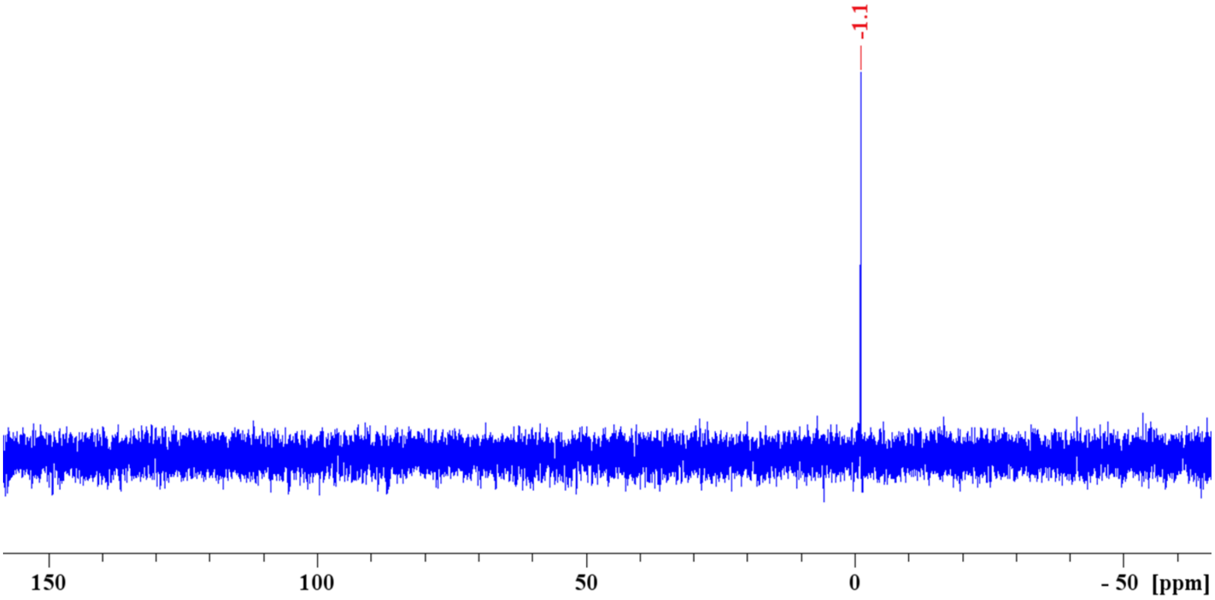

Figure S24 ^{29}Si{^{1}H} NMR spectrum of **2** in $CDCl_3$

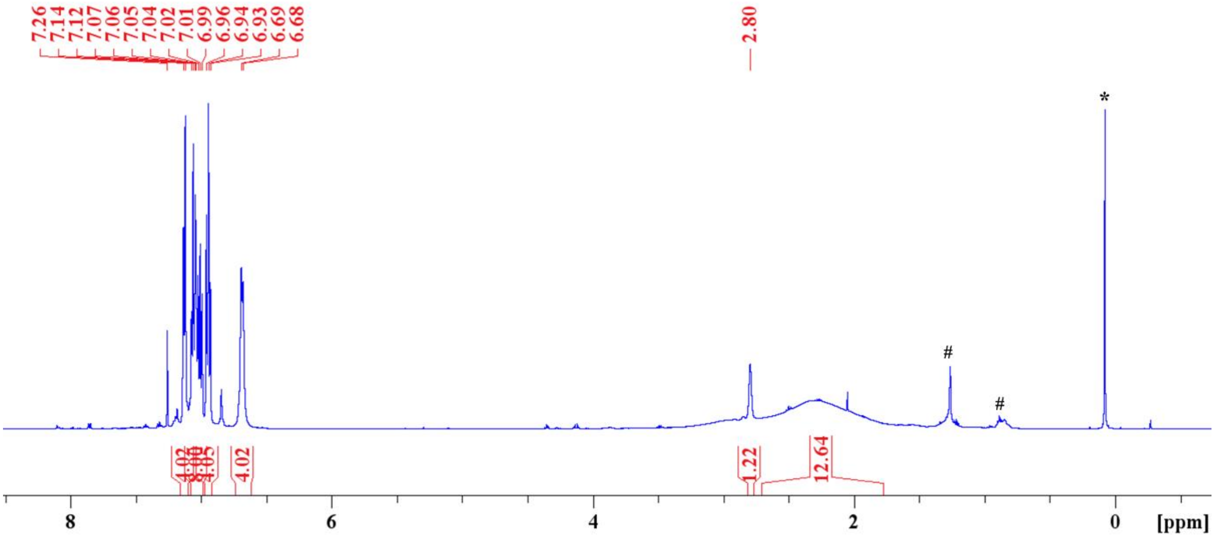

Figure S25 ^{1}H NMR spectrum of **3** in $CDCl_3$ (*: silicone grease; #: vacuum grease)

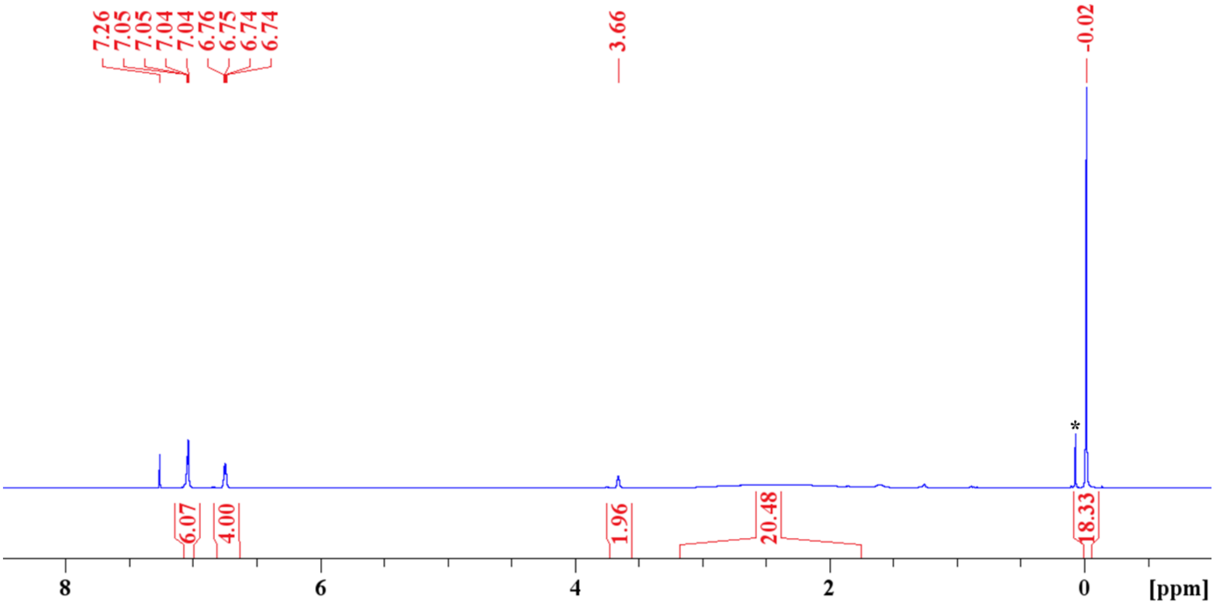

Figure S26 ^{1}H NMR spectrum of **4** in $CDCl_3$ (*: silicone grease)

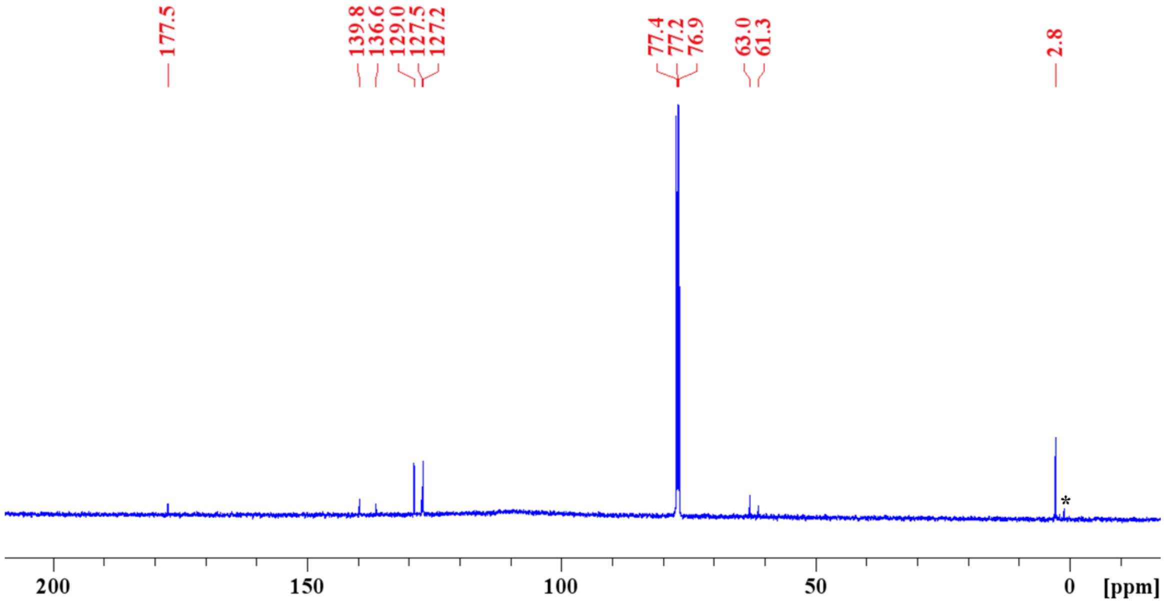

Figure S27 ^{13}C NMR spectrum of **4** in $CDCl_3$ (*: silicone grease)

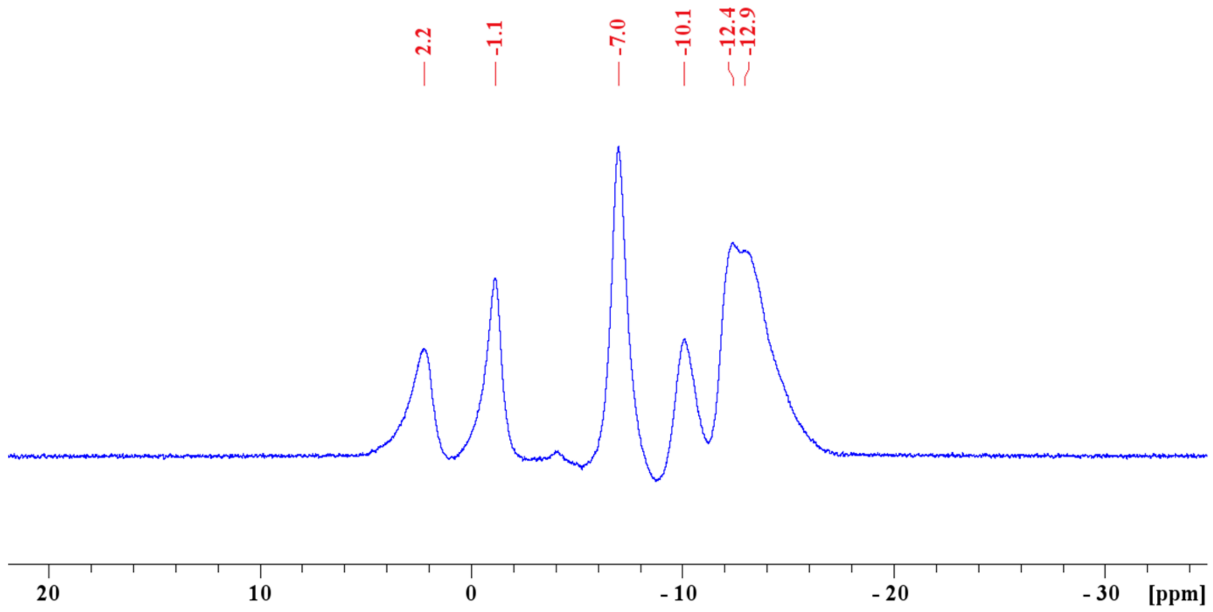

Figure S28 $^{11}B\{^1H\}$ NMR spectrum of **4** in $CDCl_3$

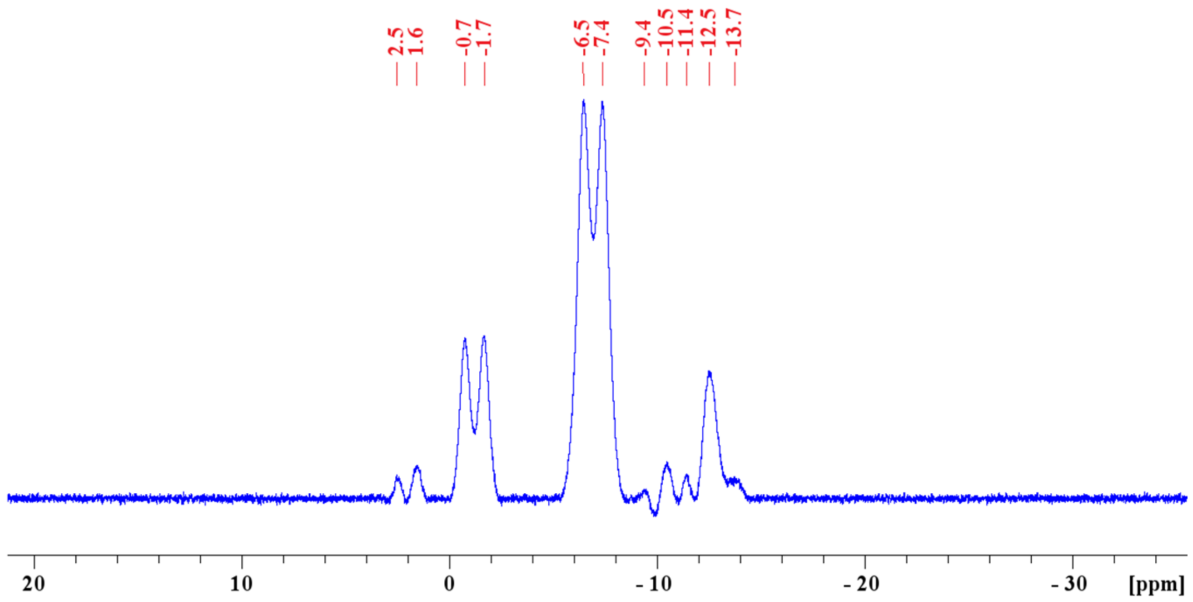

Figure S29 ^{11}B NMR spectrum of **4** in $CDCl_3$

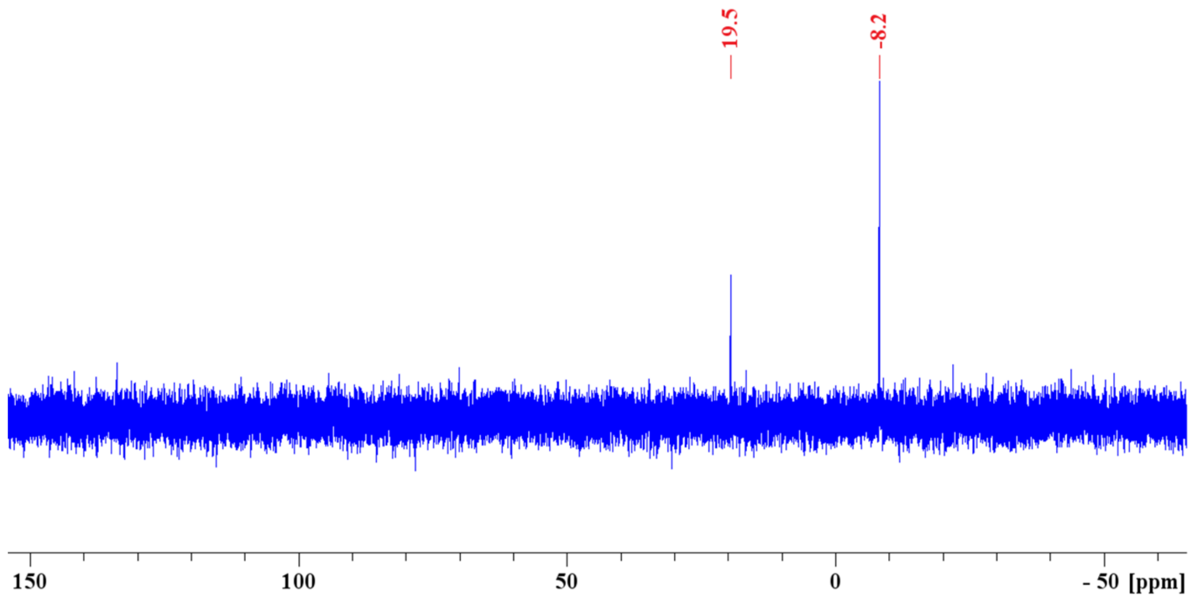

Figure S30 $^{29}Si\{^{1}H\}$ NMR spectrum of **4** in $CDCl_3$

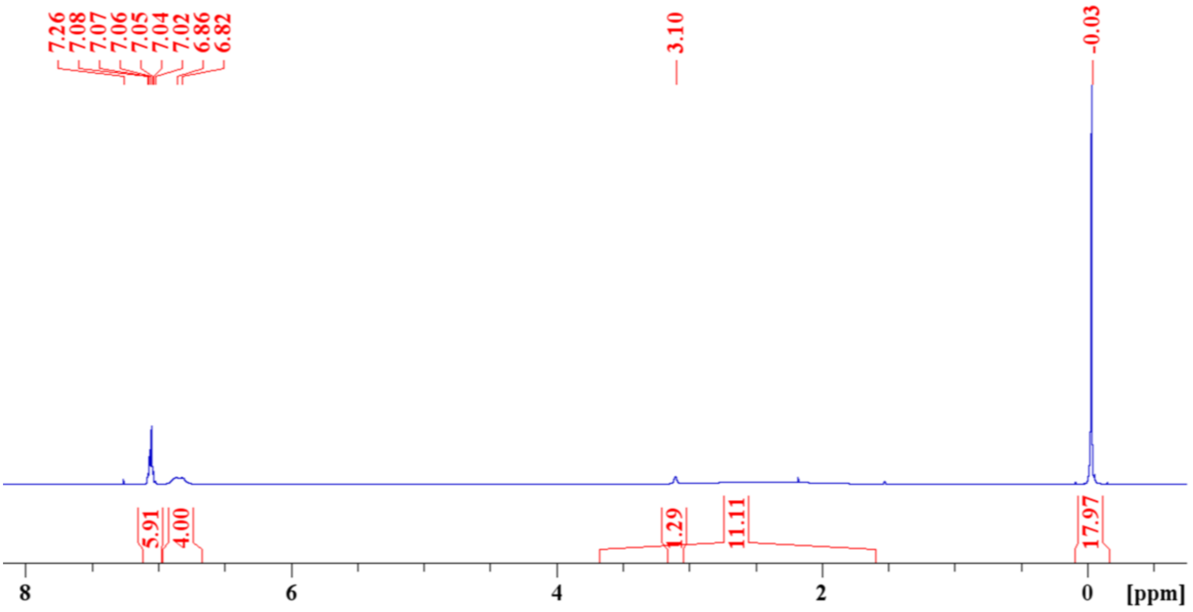

Figure S31 ^{1}H NMR spectrum of **5** in $CDCl_3$

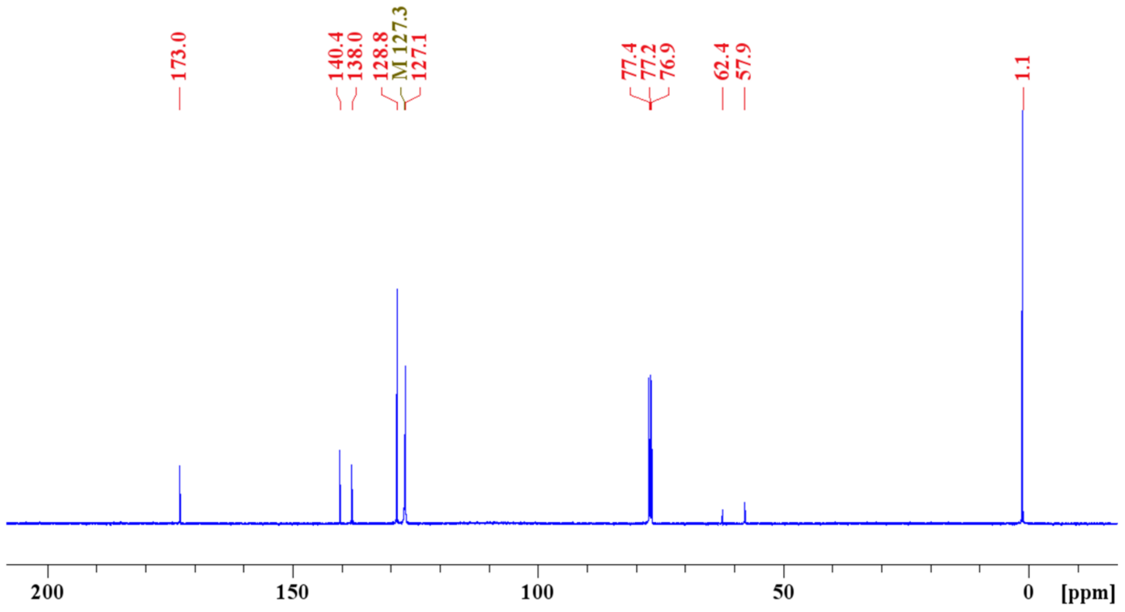

Figure S32 ^{13}C NMR spectrum of **5** in $CDCl_3$

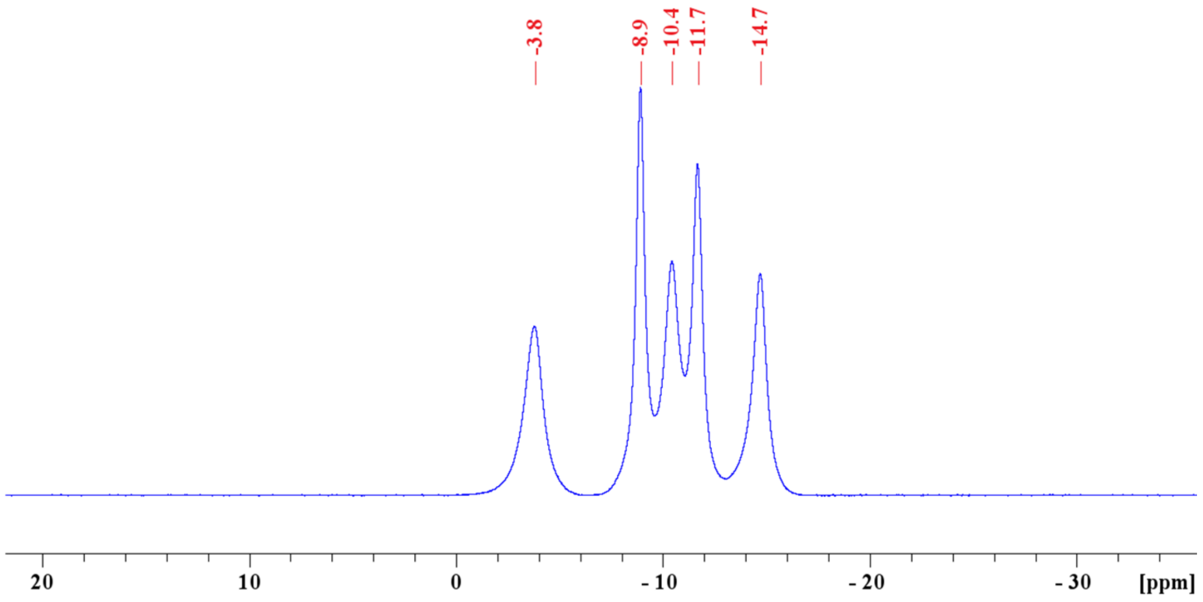

Figure S33 $^{11}B\{^1H\}$ NMR spectrum of **5** in $CDCl_3$

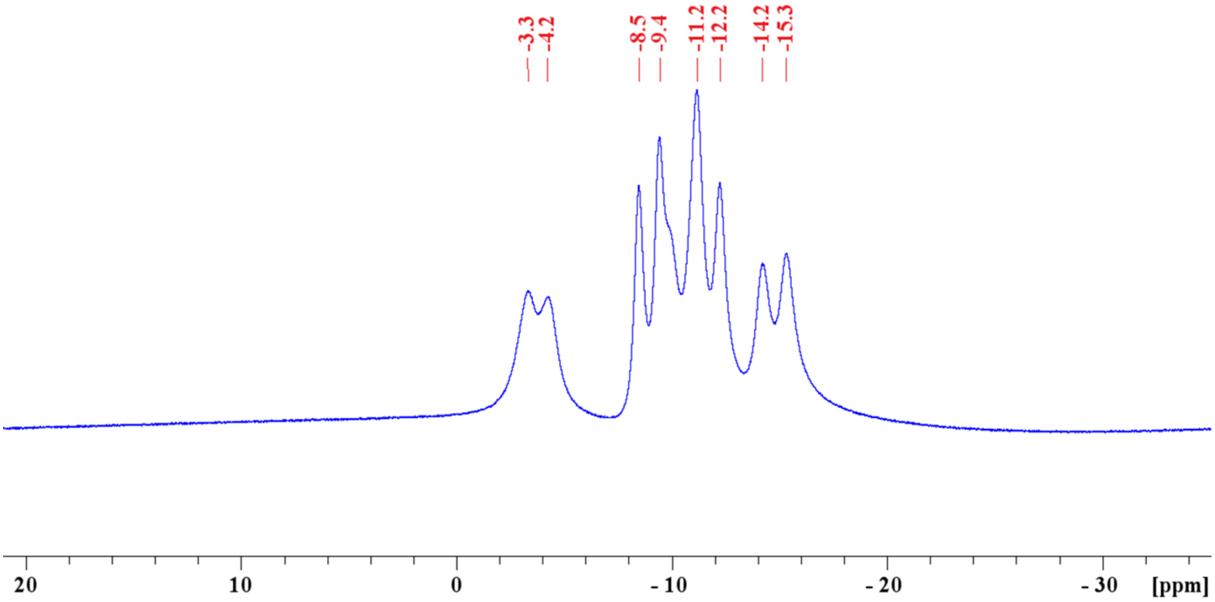

Figure S34 ^{11}B NMR spectrum of **5** in $CDCl_3$

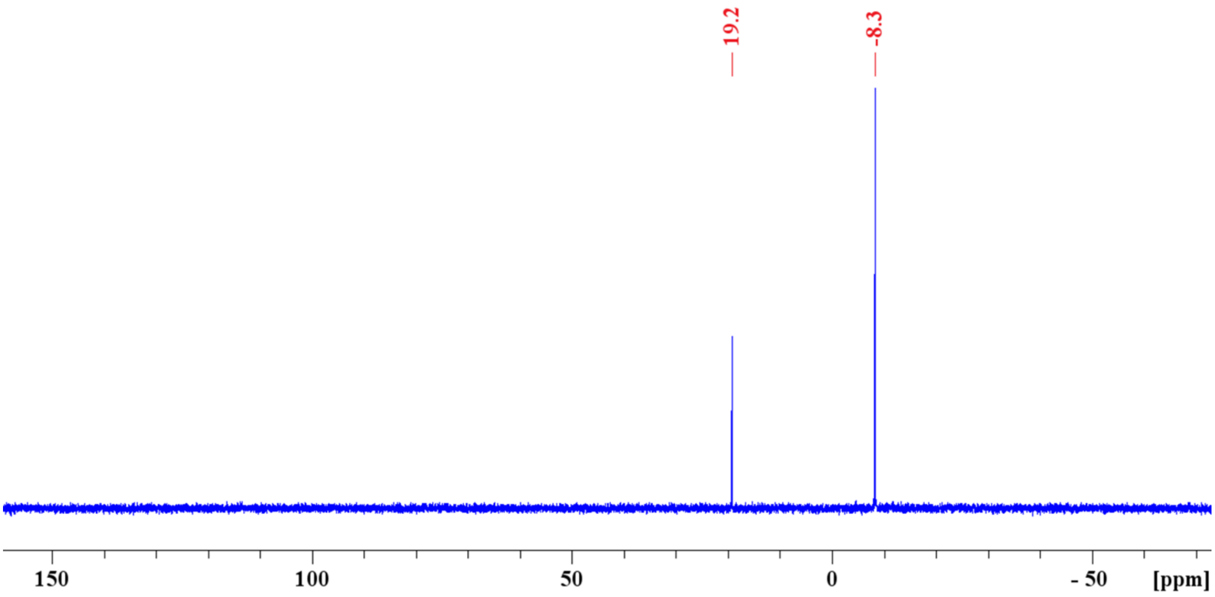

Figure S35 $^{29}Si\{^1H\}$ NMR spectrum of **5** in $CDCl_3$

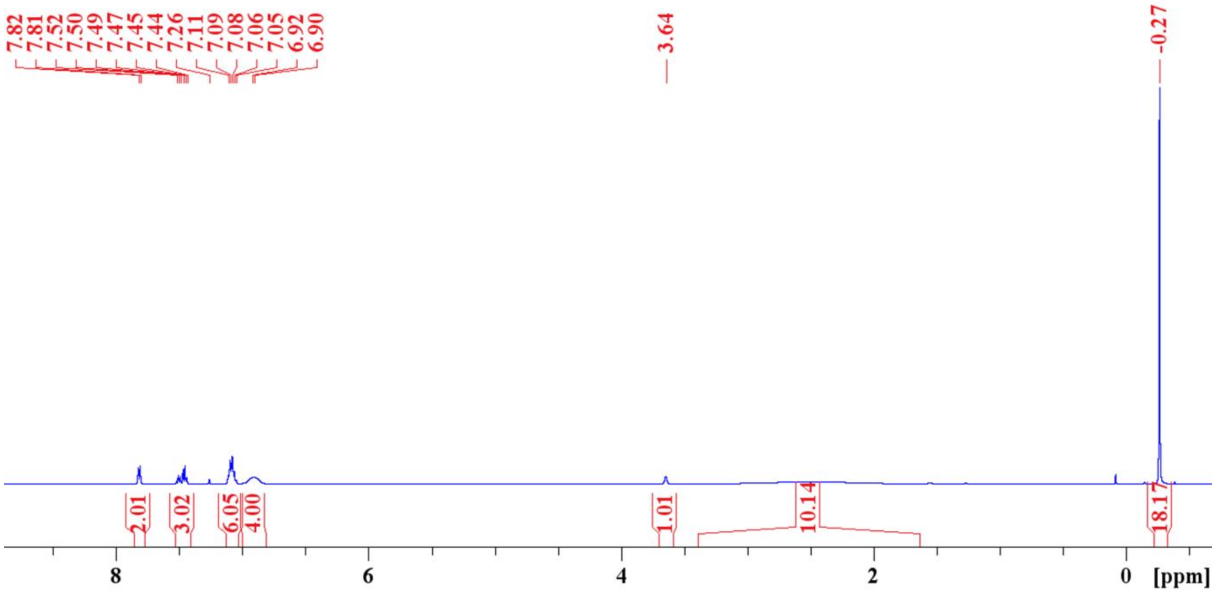

Figure S36 ^{1}H NMR spectrum of **6** in $CDCl_3$

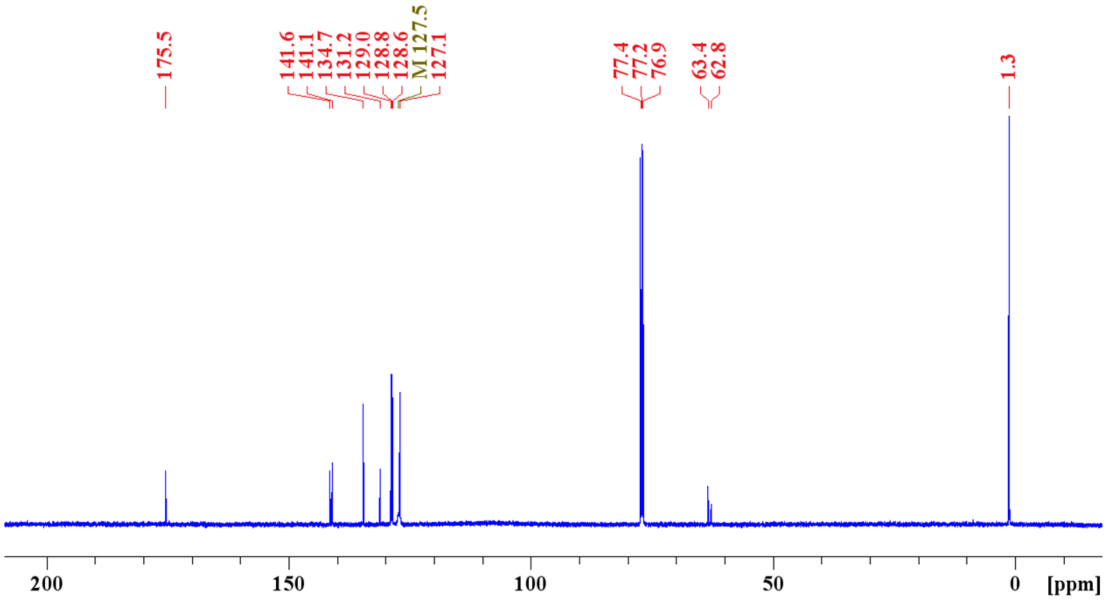

Figure S37 ^{13}C NMR spectrum of **6** in $CDCl_3$

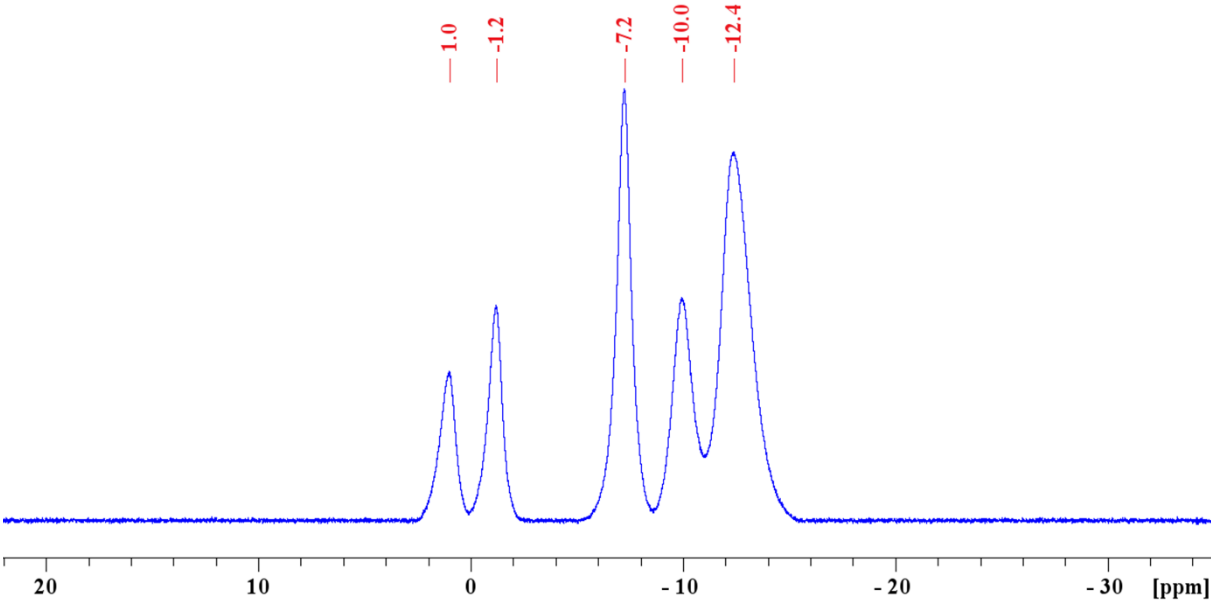

Figure S38 $^{11}B\{^{1}H\}$ NMR spectrum of **6** in $CDCl_3$

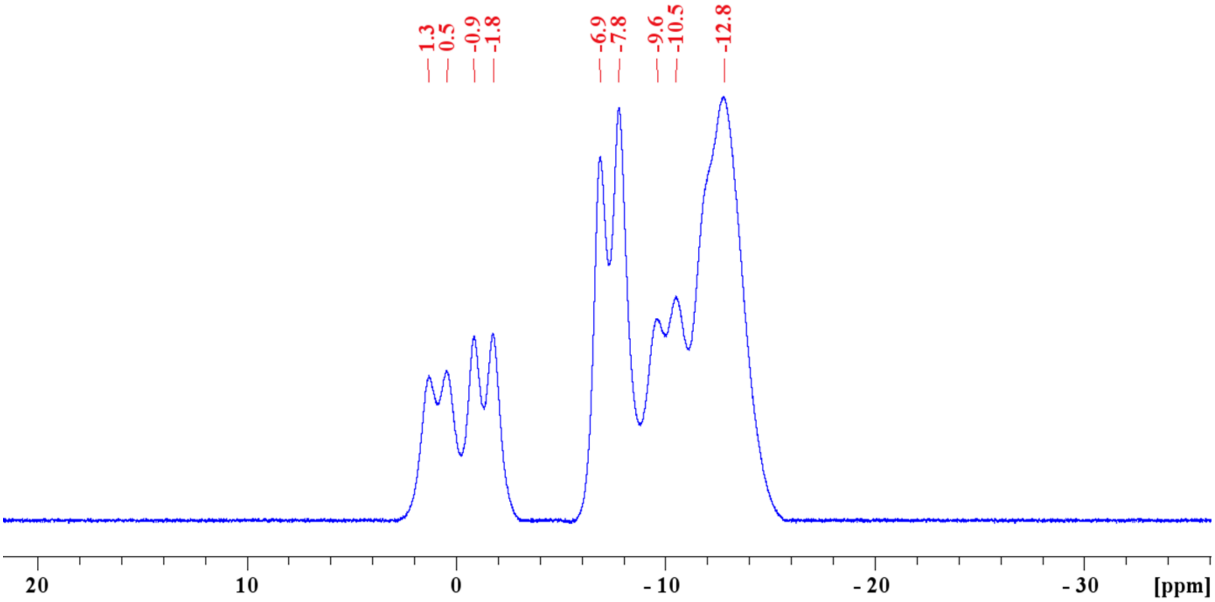

Figure S39 ^{11}B NMR spectrum of **6** in $CDCl_3$

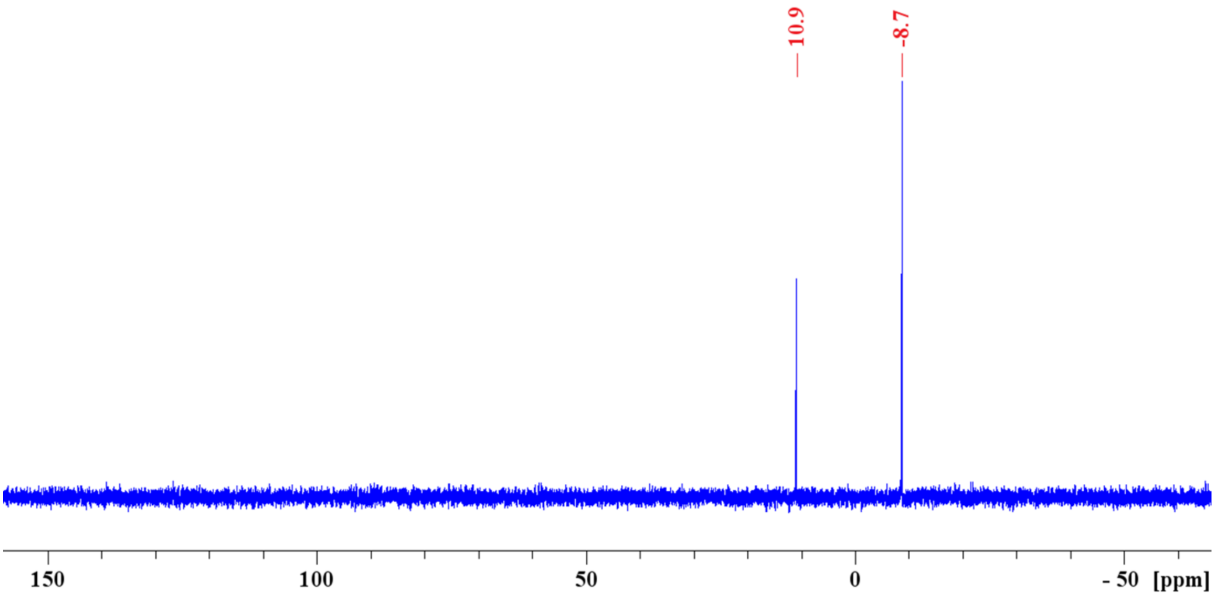

Figure S40 $^{29}Si\{^{1}H\}$ NMR spectrum of **6** in $CDCl_3$

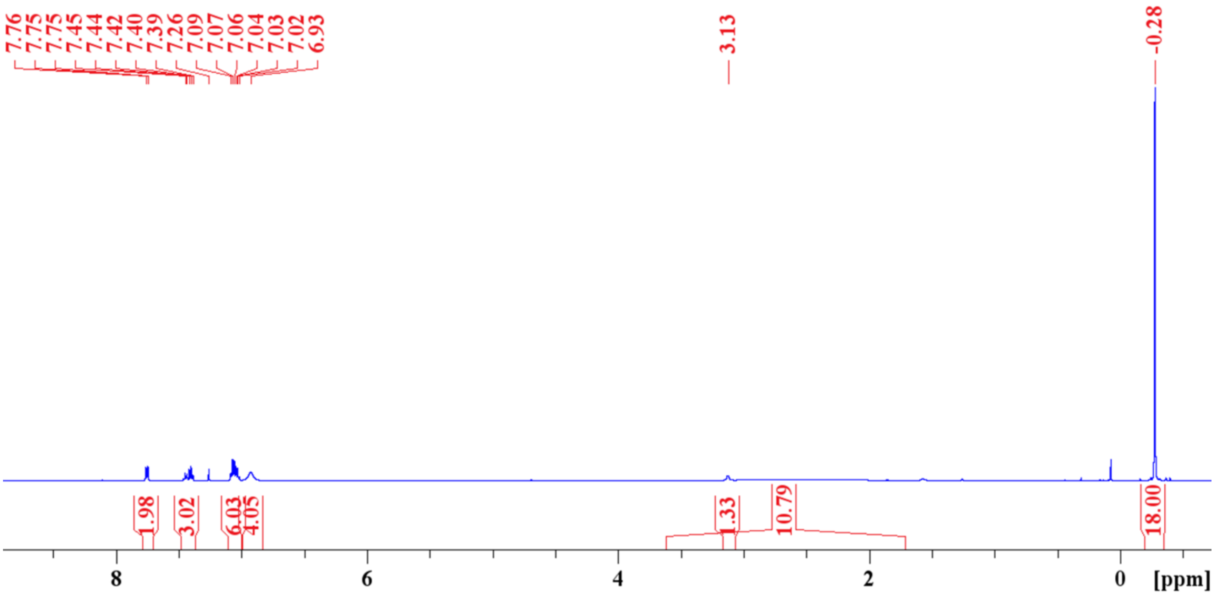

Figure S41 ^{1}H NMR spectrum of **7** in $CDCl_3$

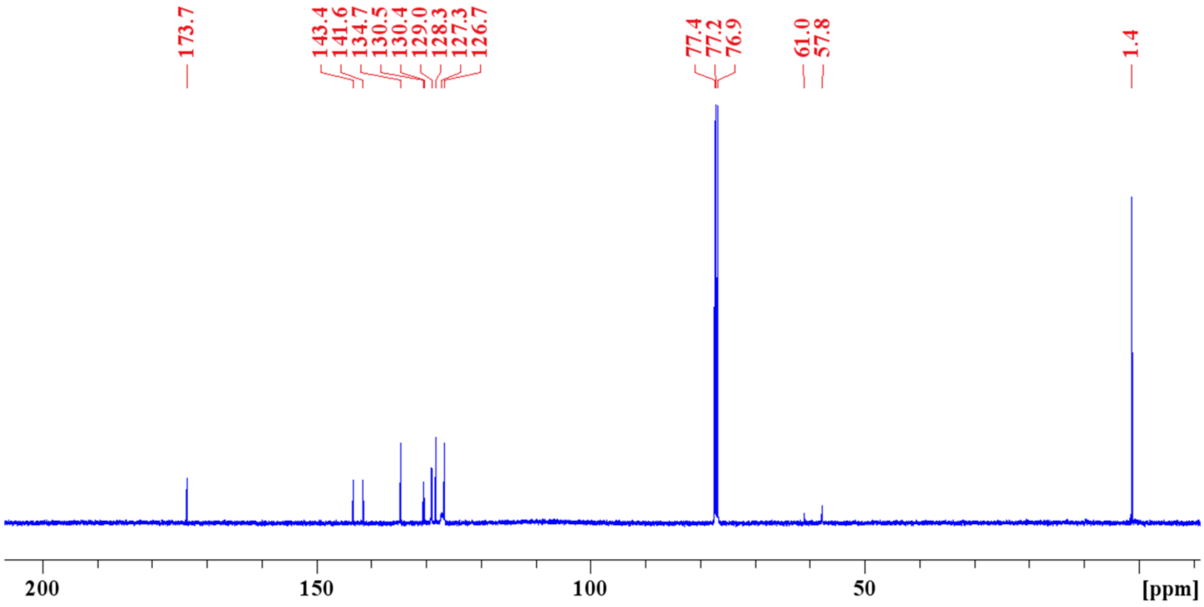

Figure S42 ^{13}C NMR spectrum of **7** in $CDCl_3$

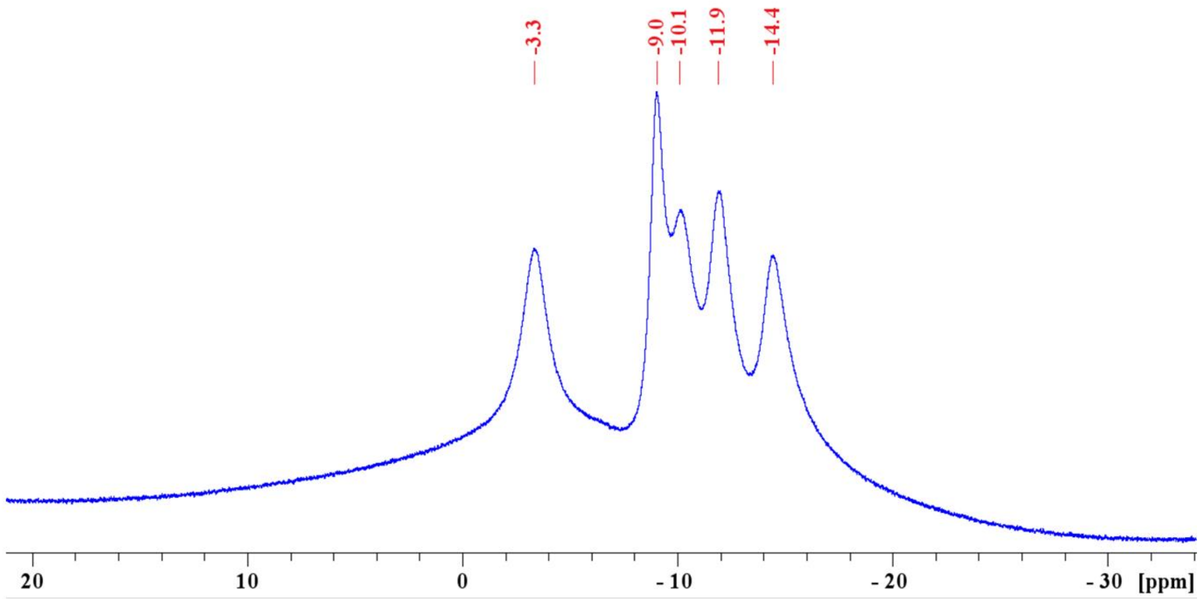

Figure S43 $^{11}B\{^1H\}$ NMR spectrum of **7** in $CDCl_3$

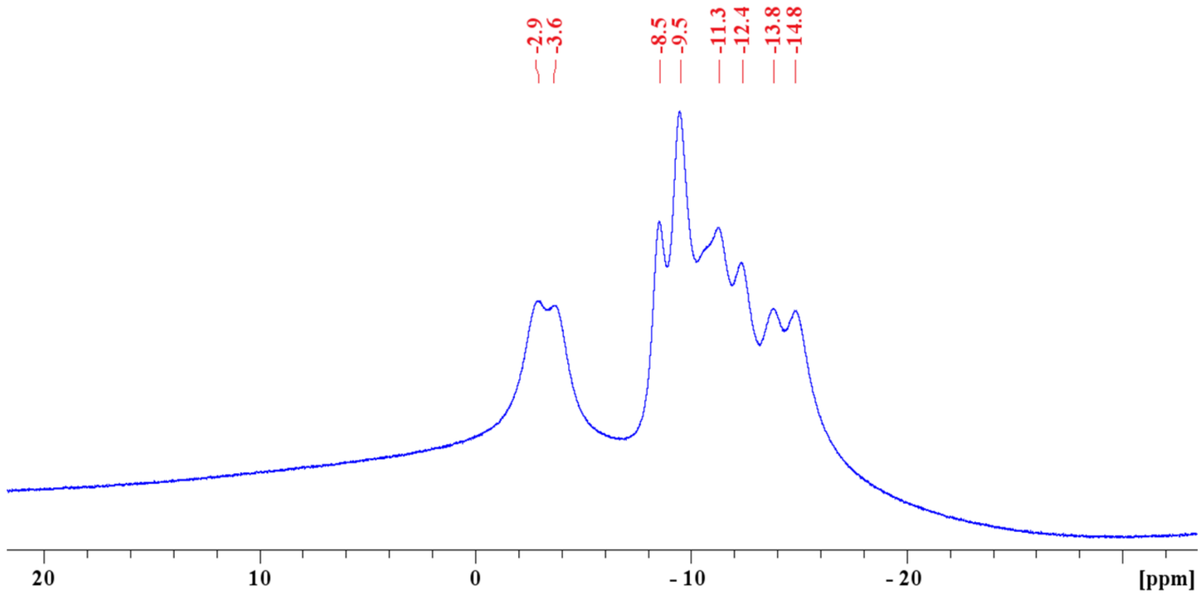

Figure S44 ^{11}B NMR spectrum of **7** in $CDCl_3$

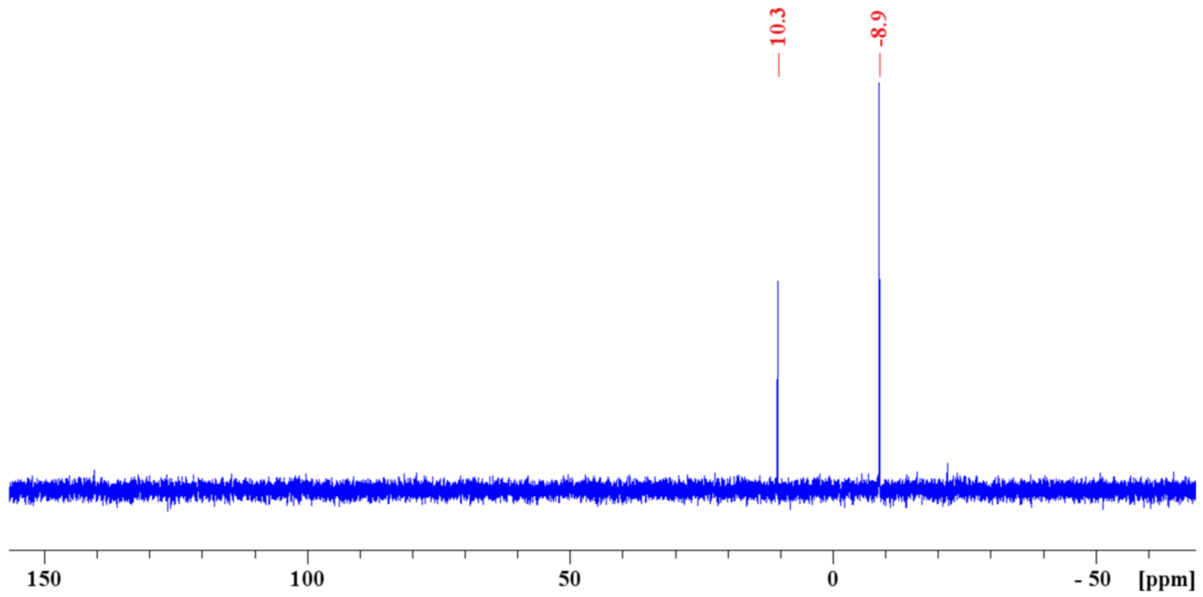

Figure S45 $^{29}Si\{^1H\}$ NMR spectrum of **7** in $CDCl_3$

IR spectra

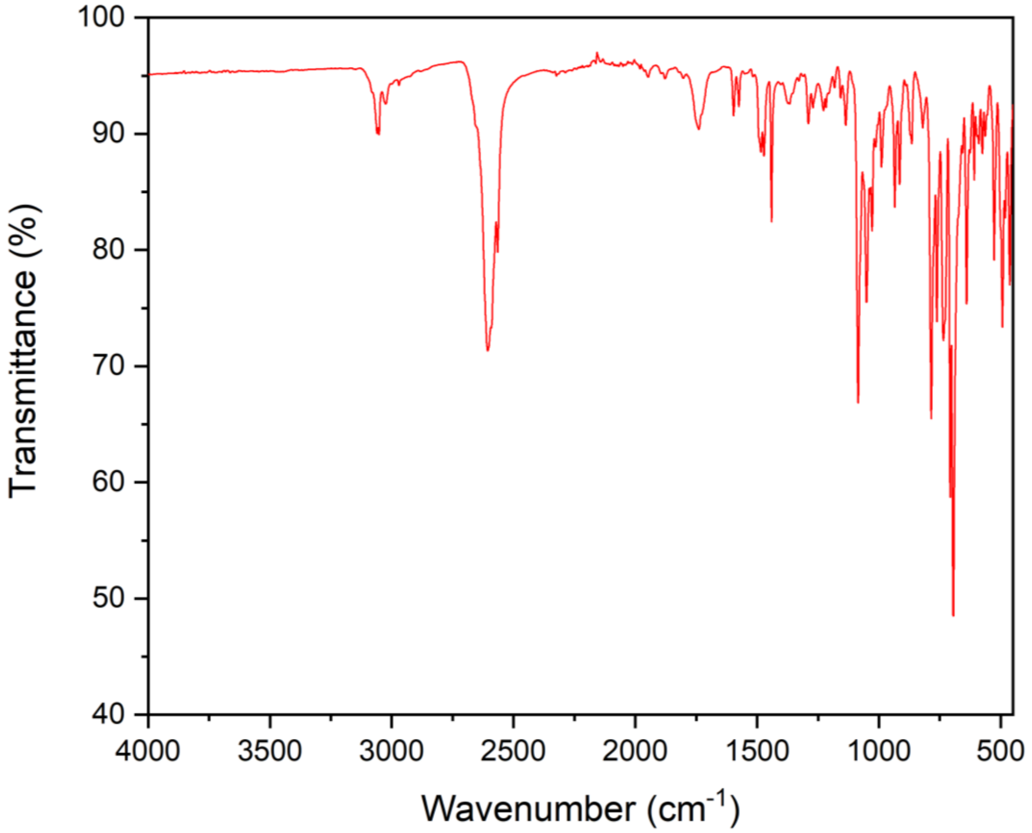

Figure S46 IR spectrum of **2**

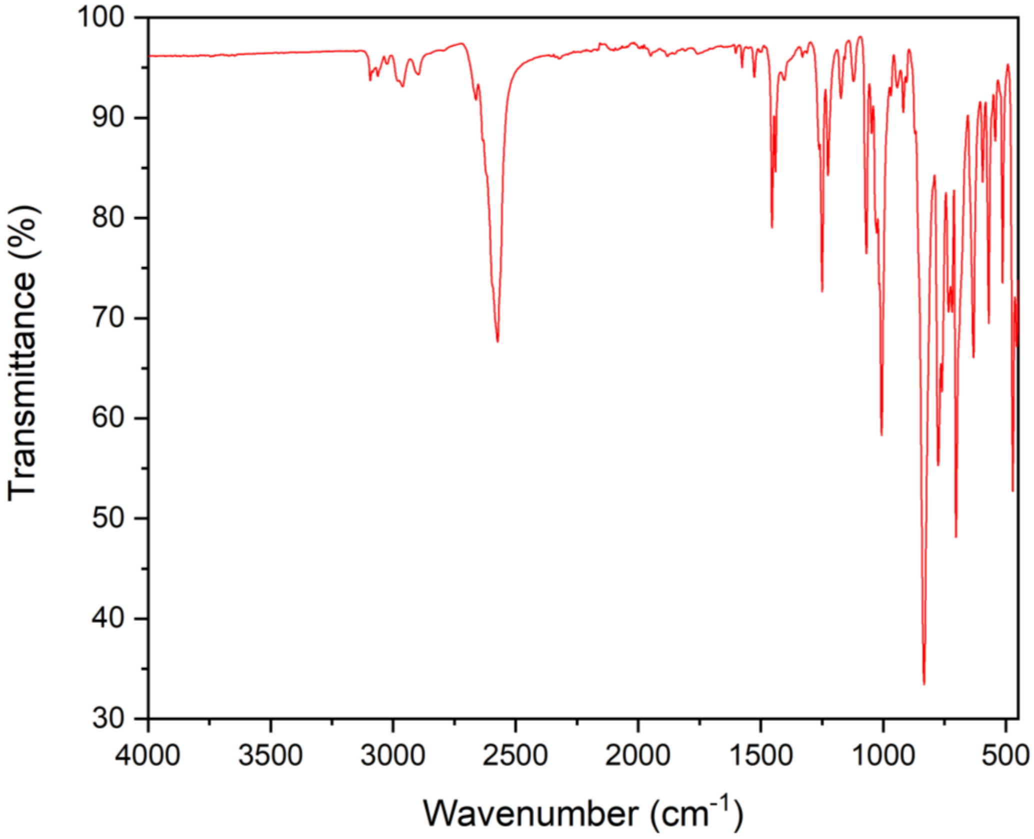

Figure S47 IR spectrum of **4**

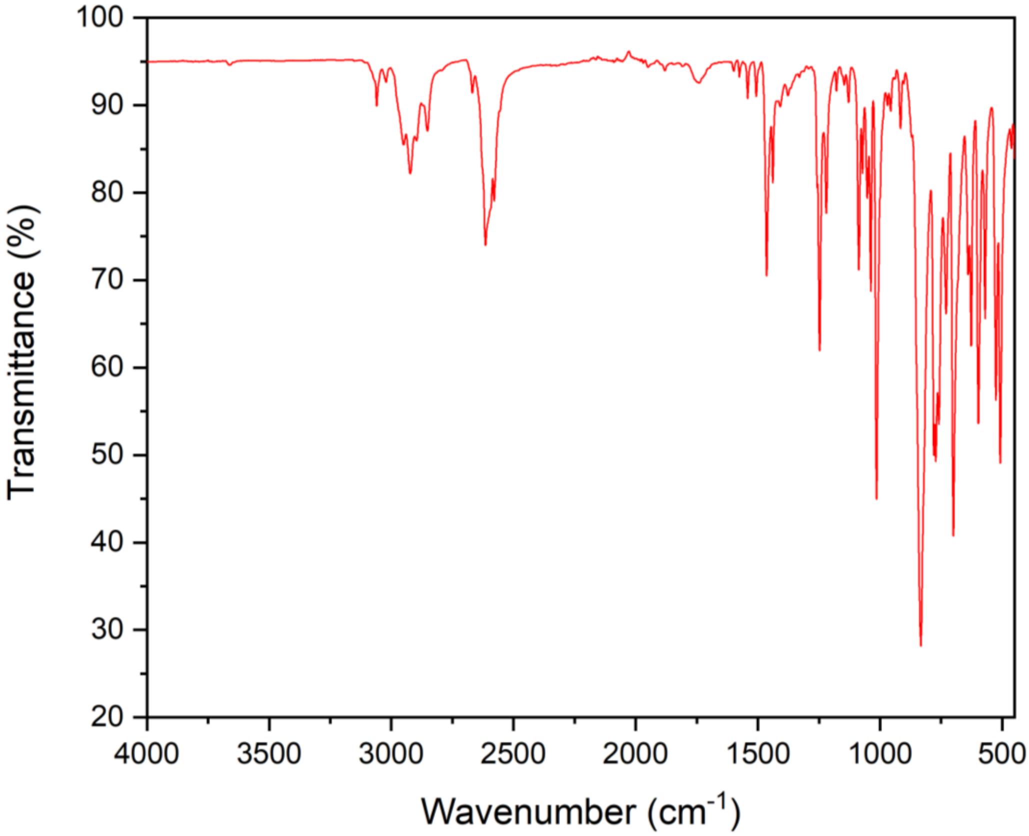

Figure S48 IR spectrum of **5**

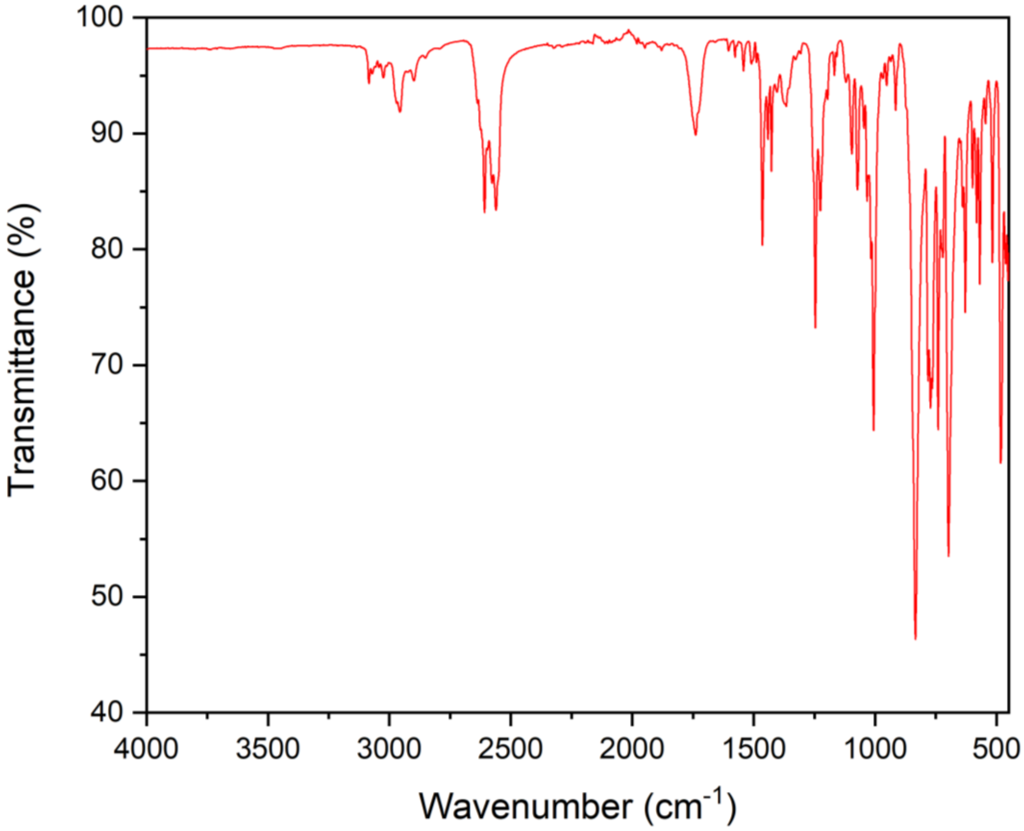

Figure S49 IR spectrum of **6**

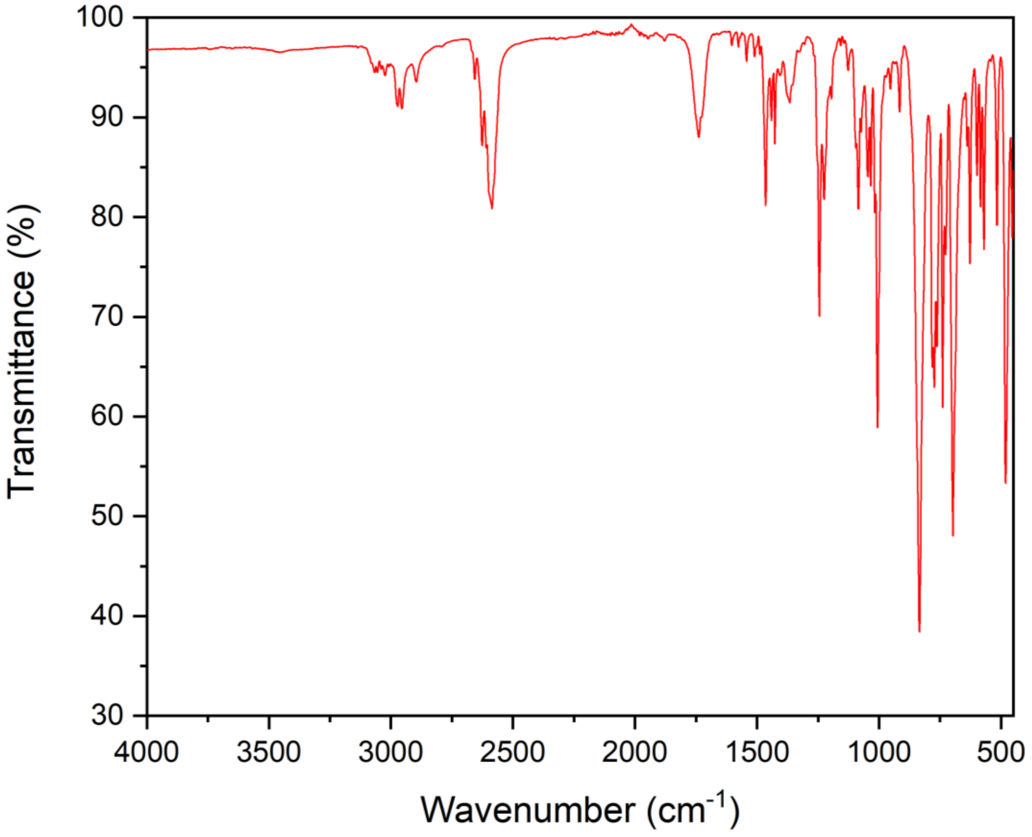

Figure S50 IR spectrum of **7**

Simulated UV-Vis spectra

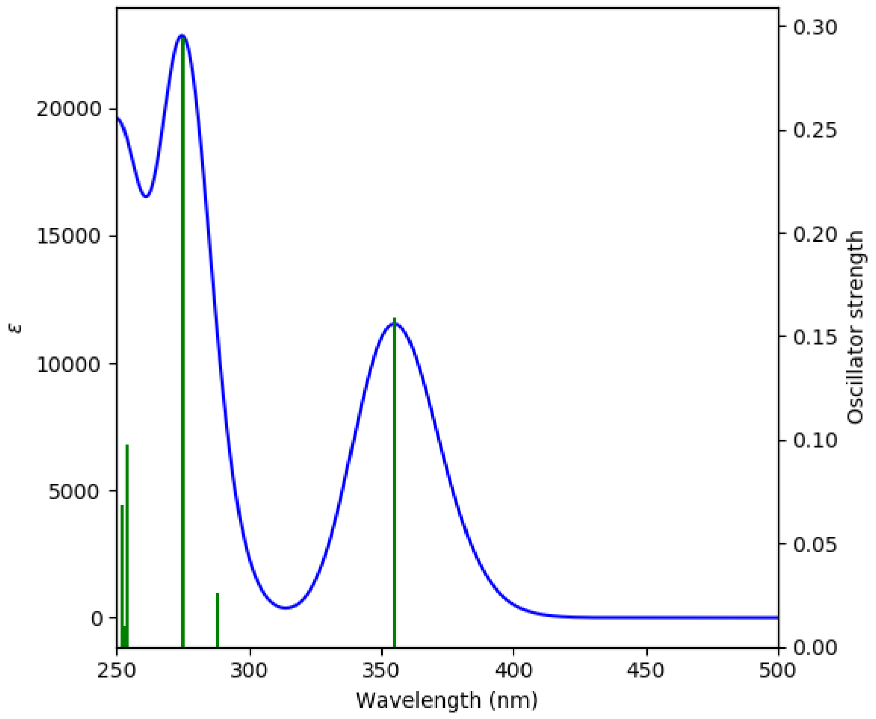

Figure S51 Simulated UV-Vis spectrum of **HPS**

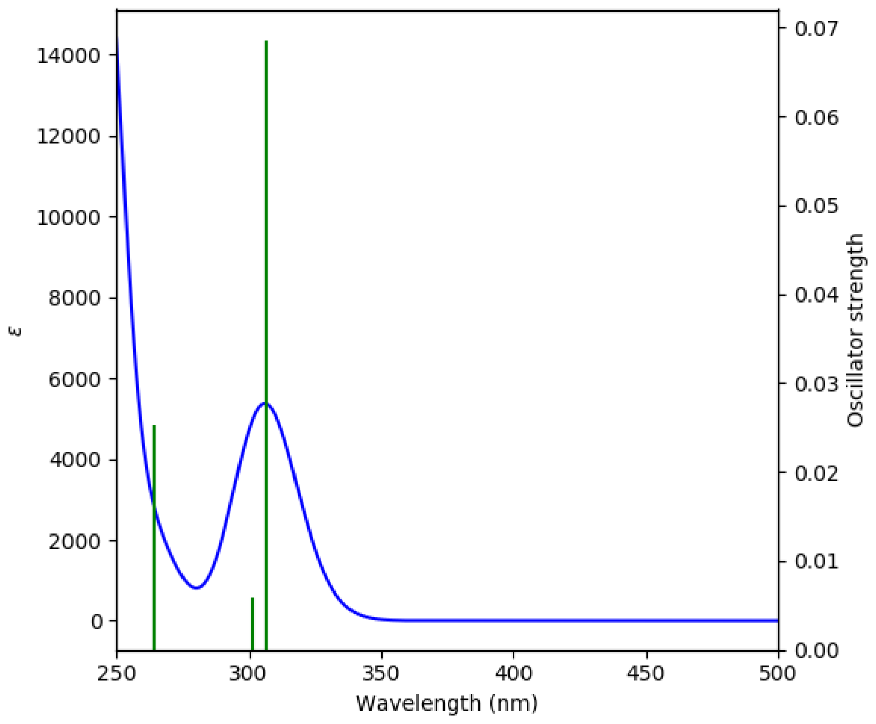

Figure S52 Simulated UV-Vis spectrum of **TPSS**

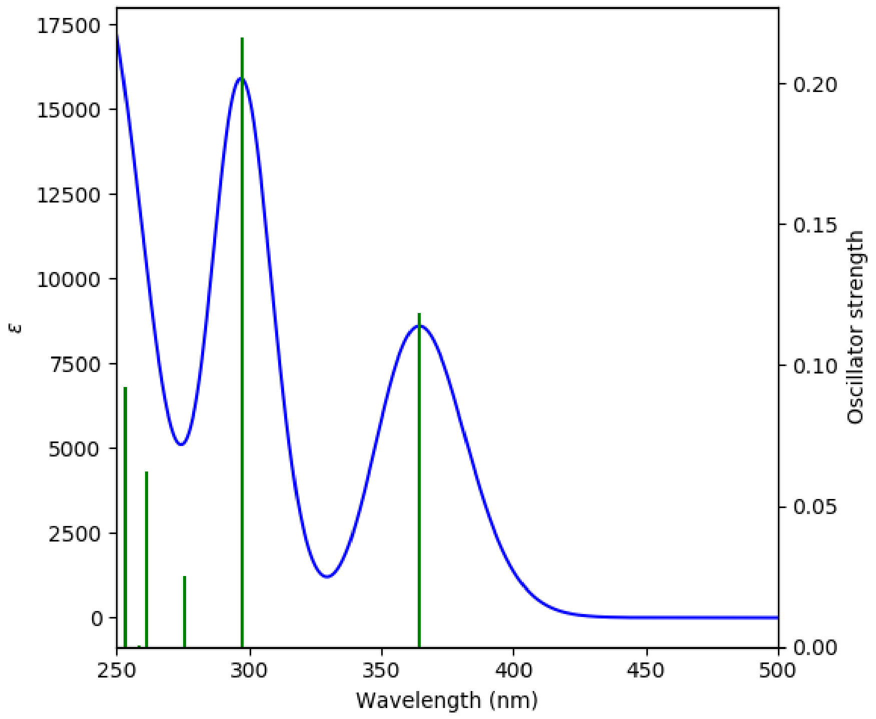

Figure S53 Simulated UV-Vis spectrum of **1**

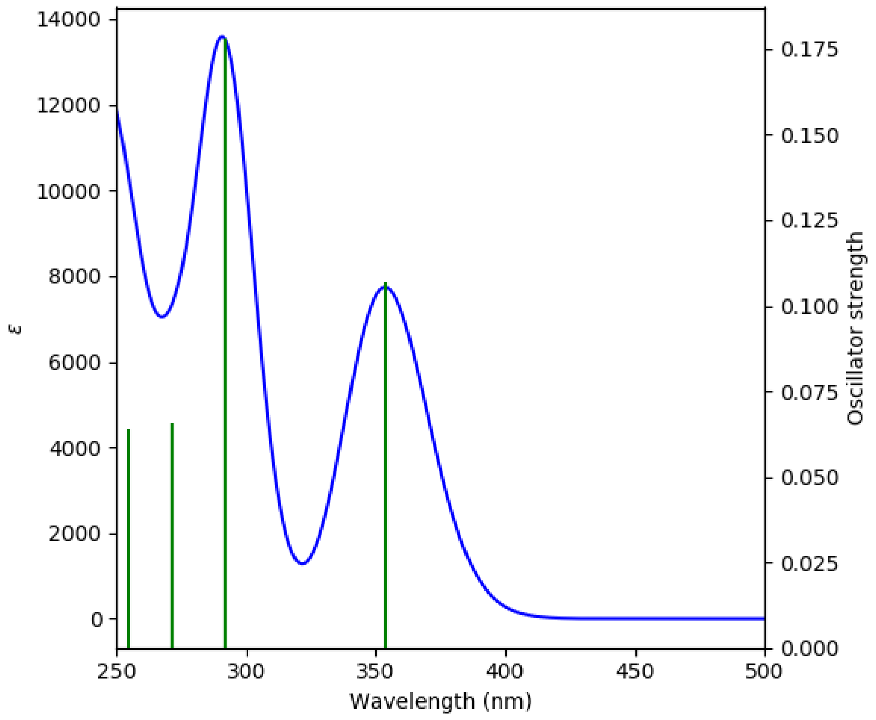

Figure S54 Simulated UV-Vis spectrum of **2**

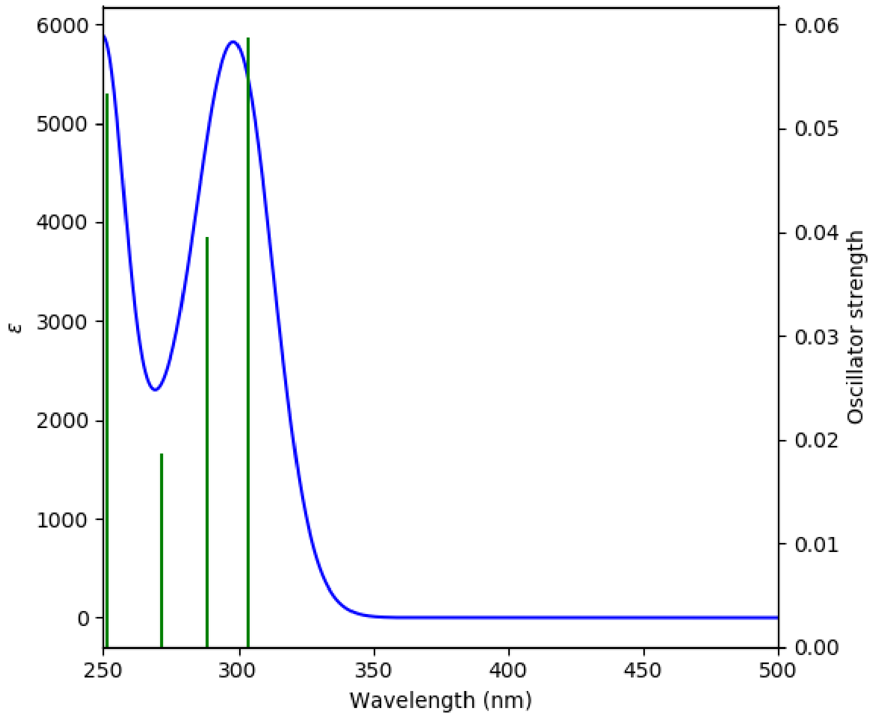

Figure S55 Simulated UV-Vis spectrum of **4**

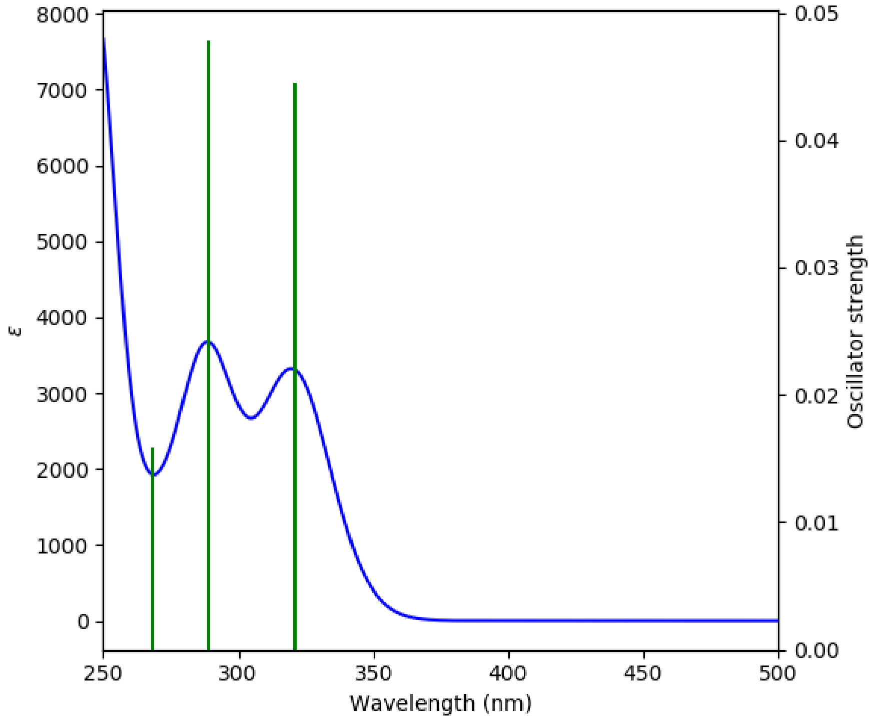

Figure S56 Simulated UV-Vis spectrum of **5**

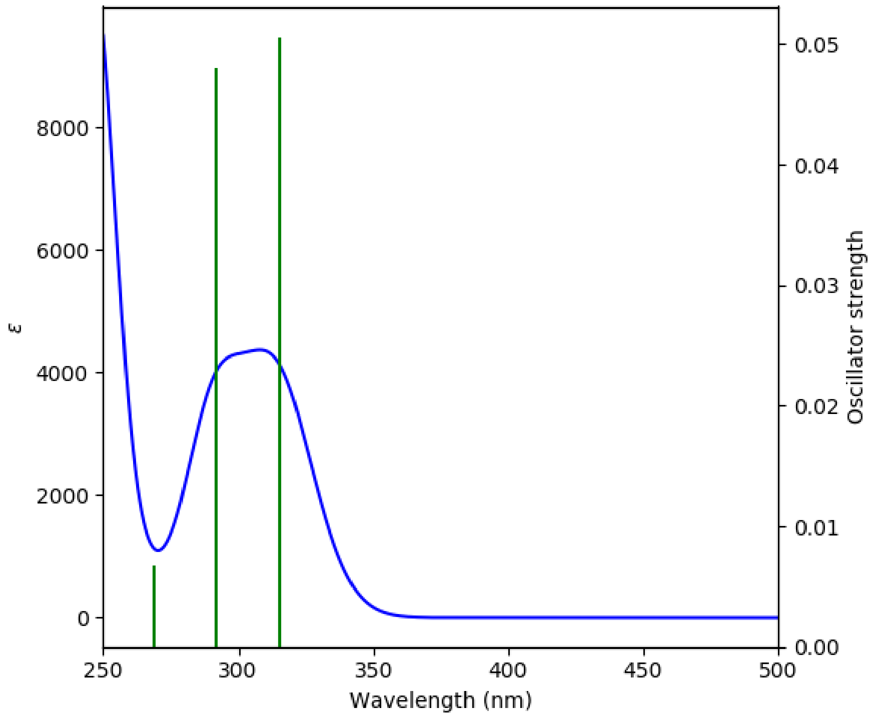

Figure S57 Simulated UV-Vis spectrum of **6**

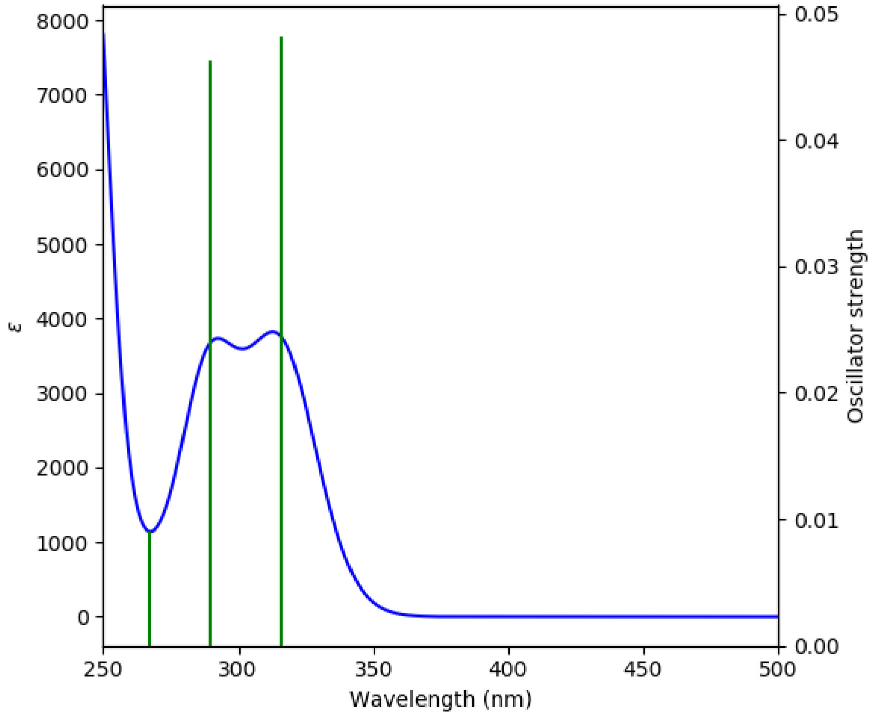

Figure S58 Simulated UV-Vis spectrum of **7**

Crystal structure and refinement details

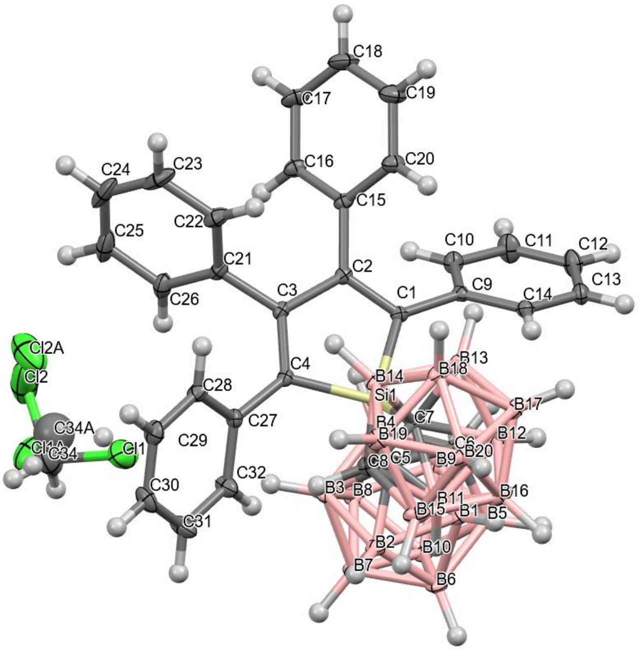

Figure S59 ORTEP view of an asymmetric unit of **1**×CH_2Cl_2 (displacement ellipsoids are drawn at the 30% probability level)

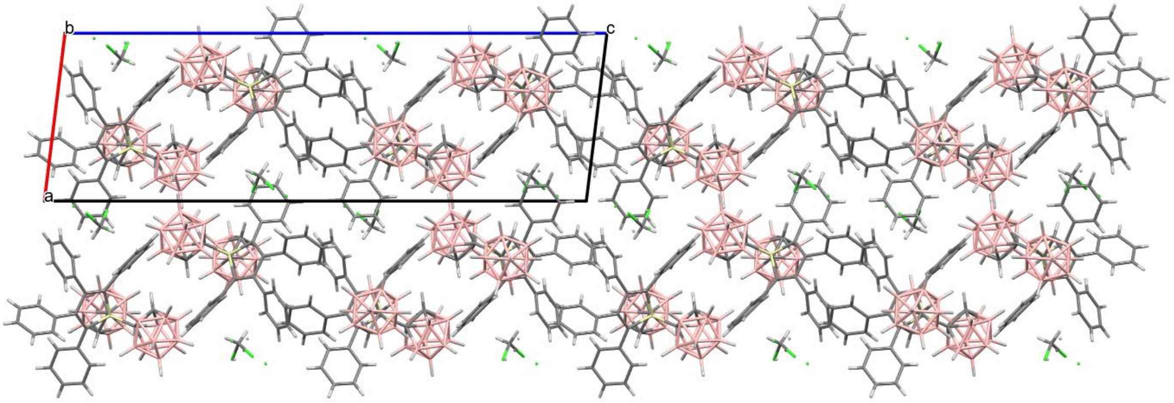

Figure S60 The molecular packing of **1**×CH_2Cl_2 showing along the **b-axis**

Experimental

Single fluorescent yellow prism-shaped crystals of **1**×CH_2Cl_2 were used as supplied. A suitable crystal with dimensions 0.34 × 0.09 × 0.08 mm^3 was selected and mounted on a Bruker Venture D8 APEX-II CCD diffractometer. The crystal was kept at a steady *T* = 150.0 K during data collection. The structure was solved with the ShelXT 2018/2[57] solution program using iterative methods and Olex2 1.5[58] as the graphical interface. The model was refined with ShelXL 2018/3[59] using full matrix least squares minimisation on $\boldsymbol{F^2}$. Data were measured using ϕ and ω scans with Mo$K\alpha$ radiation. The final completeness is 99.80 %.

All non-hydrogen atoms were refined anisotropically. Hydrogen atom positions were calculated geometrically and refined using the riding model. Disordered CH_2Cl_2 was modelled on 70%-30% ratios.

There is a single formula unit in the asymmetric unit, represented by the reported sum formula. In other words, Z is 4, and Z' is 1.

The structure of **1**×C_2Cl_2 was validated by PLATON[60], and the publication materials were prepared using OLEX2, Mercury[61] softwares.

Crystallography data including structural factors (fcf) were deposited in the Cambridge Crystallographic Data Centre (CCDC) number of 2395439.

Table S12 Crystal data and structure refinement for **1**×CH_2Cl_2.

Identification code	**1**×CH_2Cl_2
Empirical formula	$C_{33}H_{44}B_{20}Cl_2Si$
Formula weight	755.87
Temperature/K	150.00
Crystal system	monoclinic
Space group	$P2_1/c$
a/Å	11.0625(7)
b/Å	10.5884(5)
c/Å	35.4898(19)
α/°	90
β/°	97.016(2)
γ/°	90
Volume/Å^3	4125.9(4)
Z	4
ρ_{calc}g/cm^3	1.217
μ/mm^{-1}	0.214
F(000)	1560.0
Crystal size/mm^3	0.339 × 0.093 × 0.084

Radiation	MoKα (λ = 0.71073)
2Θ range for data collection/°	3.71 to 50.698
Index ranges	-12 ≤ *h* ≤ 13, -12 ≤ *k* ≤ 12, -42 ≤ *l* ≤ 42
Reflections collected	43227
Independent reflections	7546 [R_{int} = 0.0681, R_{sigma} = 0.0474]
Data/restraints/parameters	7546/3/528
Goodness-of-fit on F^2	1.044
Final R indexes [I>=2σ (I)]	R_1 = 0.0537, wR_2 = 0.1319
Final R indexes [all data]	R_1 = 0.0626, wR_2 = 0.1381
Largest diff. peak/hole / e Å^{-3}	0.51/-0.53

Table S13 Bond Lengths for **1**×CH_2Cl_2.

Atom	Atom	Length/Å	Atom	Atom	Length/Å
Si1	C1	1.860(2)	C29	C30	1.378(3)
Si1	C4	1.8653(19)	C30	C31	1.382(3)
Si1	C5	1.9191(19)	C31	C32	1.383(3)
Si1	C7	1.923(2)	B1	B2	1.780(3)
C1	C2	1.361(3)	B1	B5	1.779(3)
C1	C9	1.487(3)	B1	B6	1.770(3)
C2	C3	1.504(3)	B2	B3	1.784(3)
C2	C15	1.489(3)	B2	B6	1.783(3)
C3	C4	1.361(3)	B2	B7	1.781(3)
C3	C21	1.486(3)	B3	B4	1.772(3)
C4	C27	1.484(3)	B3	B7	1.779(3)
C5	C6	1.665(3)	B3	B8	1.772(3)
C5	B1	1.735(3)	B4	B8	1.774(3)
C5	B2	1.712(3)	B4	B9	1.779(3)
C5	B3	1.724(3)	B5	B6	1.778(4)
C5	B4	1.738(3)	B5	B9	1.776(3)
C6	B1	1.711(3)	B5	B10	1.771(4)
C6	B4	1.708(3)	B6	B7	1.784(4)
C6	B5	1.692(3)	B6	B10	1.793(4)
C6	B9	1.694(3)	B7	B8	1.793(4)
C7	C8	1.668(3)	B7	B10	1.779(3)
C7	B11	1.725(3)	B8	B9	1.777(4)
C7	B12	1.716(3)	B8	B10	1.792(3)
C7	B13	1.723(3)	B9	B10	1.780(3)
C7	B14	1.743(3)	B11	B12	1.775(4)
C8	B11	1.713(3)	B11	B15	1.775(3)
C8	B14	1.710(3)	B11	B16	1.763(4)
C8	B15	1.702(3)	B12	B13	1.776(3)

C8	B19	1.698(3)	B12	B16	1.779(3)
C9	C10	1.399(3)	B12	B17	1.781(3)
C9	C14	1.394(3)	B13	B14	1.772(3)
C10	C11	1.383(3)	B13	B17	1.778(3)
C11	C12	1.381(3)	B13	B18	1.776(3)
C12	C13	1.376(3)	B14	B18	1.766(3)
C13	C14	1.385(3)	B14	B19	1.779(3)
C15	C16	1.396(3)	B15	B16	1.781(4)
C15	C20	1.393(3)	B15	B19	1.779(3)
C16	C17	1.389(3)	B15	B20	1.768(4)
C17	C18	1.372(4)	B16	B17	1.783(4)
C18	C19	1.381(4)	B16	B20	1.785(3)
C19	C20	1.386(3)	B17	B18	1.786(3)
C21	C22	1.388(3)	B17	B20	1.776(4)
C21	C26	1.388(3)	B18	B19	1.772(4)
C22	C23	1.386(3)	B18	B20	1.787(3)
C23	C24	1.366(5)	B19	B20	1.776(4)
C24	C25	1.369(5)	Cl1	C34	1.750(6)
C25	C26	1.393(3)	Cl2	C34	1.706(6)
C27	C28	1.402(3)	Cl2A	C34A	1.716(15)
C27	C32	1.391(3)	C34A	Cl1A	1.773(17)
C28	C29	1.383(3)			

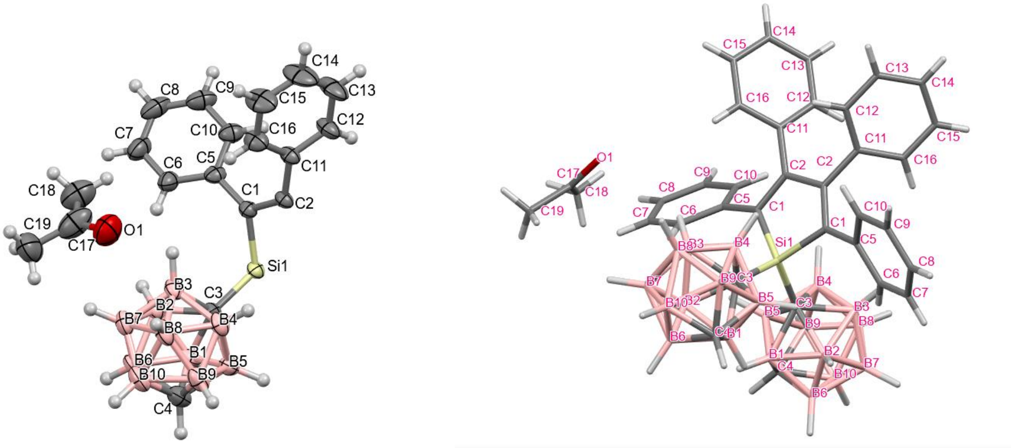

Figure S61 ORTEP view of an asymmetric unit of **2**×C_3H_6O (displacement ellipsoids are drawn at the 30% probability level) and capped sticks presentation of the symmetry generated molecule (1-X,+Y,3/2-Z)

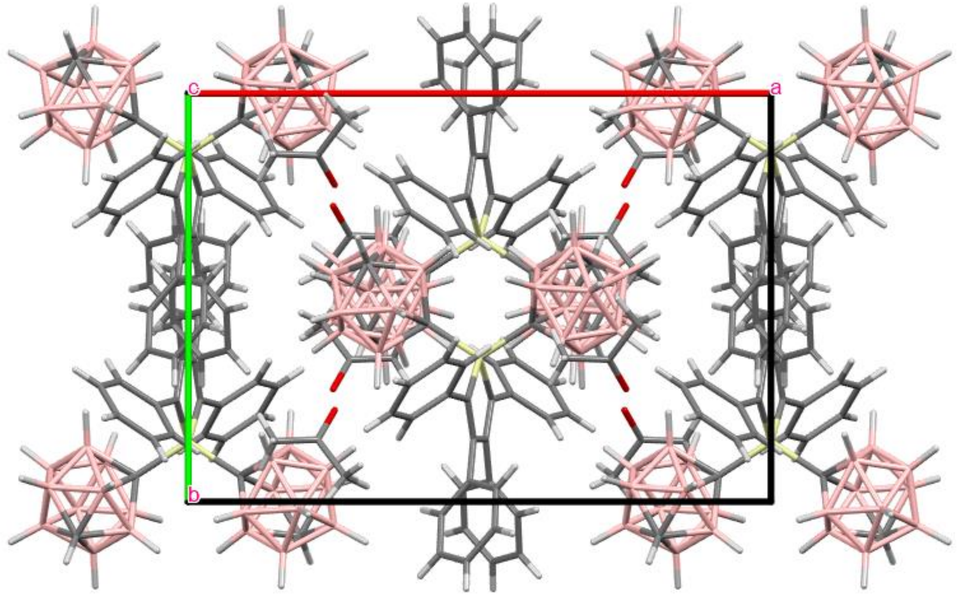

Figure S62 The molecular packing of **2**×C_3H_6O showing along the **c-axis**

Experimental

Single colourless block-shaped crystals of **2**×C_3H_6O were used as supplied. A suitable crystal with dimensions 0.56 × 0.24 × 0.19 mm^3 was selected and mounted on a Bruker Venture D8 APEX-II CCD diffractometer. During data collection, the crystal was kept at a steady T = 296(2) K. Data were measured using ϕ and ω scans with MoKα radiation. The final completeness is 99.40 %.

The structure was solved with the ShelXT[57] solution program using iterative methods and Olex2 1.5[58] as the graphical interface. The model was refined with ShelXL 2018/3[59] using full matrix least squares minimisation on $\boldsymbol{F^2}$.

All non-hydrogen atoms were refined anisotropically. Hydrogen atom positions were calculated geometrically and refined using the riding model.

The value of Z' is 0.5. This means that only half of the formula unit is present in the asymmetric unit, with the other half consisting of symmetry equivalent atoms.

The structure of **2**×C_3H_6O was validated by PLATON[60], and the publication materials were prepared using OLEX2, Mercury[61] softwares.

Crystallography data including structural factors (fcf) were deposited in the Cambridge Crystallographic Data Centre (CCDC) number of 2395432.

Table S14. Crystal data and structure refinement for **2**×C_3H_6O

Identification code	**2**×C_3H_6O
Empirical formula	$C_{35}H_{48}B_{20}OSi$
Formula weight	729.02
Temperature/K	296.15
Crystal system	monoclinic
Space group	*C2/c*
a/Å	18.1509(15)
b/Å	12.4086(9)
c/Å	20.5343(14)
α/°	90
β/°	102.767(3)
γ/°	90
Volume/Å^3	4510.5(6)
Z	4
ρ_{calc}g/cm^3	1.074
μ/mm^{-1}	0.080
F(000)	1520.0
Crystal size/mm^3	0.563 × 0.241 × 0.192
Radiation	MoKα (λ = 0.71073)
2Θ range for data collection/°	5.338 to 50.678
Index ranges	$-21 \le h \le 21$, $-14 \le k \le 14$, $-24 \le l \le 24$
Reflections collected	30314
Independent reflections	4110 [R_{int} = 0.1391, R_{sigma} = 0.0630]
Data/restraints/parameters	4110/0/278
Goodness-of-fit on F^2	1.031
Final R indexes [I>=2σ (I)]	R_1 = 0.0694, wR_2 = 0.1803
Final R indexes [all data]	R_1 = 0.1162, wR_2 = 0.2122
Largest diff. peak/hole / e Å^{-3}	0.30/-0.20

Table S15 Bond Lengths for **2**×C_3H_6O

Atom	Atom	Length/Å	Atom	Atom	Length/Å
Si1	C3^1	1.911(3)	C4	B6	1.710(5)
Si1	C3	1.911(3)	C4	B9	1.714(5)
Si1	C1^1	1.871(3)	C4	B10	1.705(6)
Si1	C1	1.871(3)	B1	B2	1.756(5)
C2	C2^1	1.497(5)	B1	B5	1.759(5)
C2	C11	1.487(3)	B1	B6	1.752(6)
C2	C1	1.355(4)	B2	B3	1.768(5)
C11	C16	1.380(4)	B2	B6	1.756(5)

C11	C12	1.381(4)	B2	B7	1.774(6)
C16	C15	1.384(5)	B3	B4	1.771(5)
C15	C14	1.386(7)	B3	B7	1.761(5)
C14	C13	1.352(7)	B3	B8	1.746(6)
C13	C12	1.368(5)	B4	B5	1.759(6)
C5	C10	1.387(4)	B4	B8	1.751(5)
C5	C6	1.382(4)	B4	B9	1.747(5)
C5	C1	1.486(4)	B5	B9	1.756(6)
C10	C9	1.386(5)	B6	B7	1.758(6)
C9	C8	1.363(6)	B6	B10	1.758(6)
C8	C7	1.361(6)	B7	B8	1.757(6)
C7	C6	1.368(5)	B7	B10	1.757(6)
C3	B1	1.708(4)	B8	B9	1.740(6)
C3	B2	1.716(4)	B8	B10	1.750(6)
C3	B3	1.728(5)	B9	B10	1.775(6)
C3	B4	1.734(4)	O1	C17	1.306(13)
C3	B5	1.722(4)	C19	C17	1.519(17)
C4	B1	1.705(5)	C18	C17	1.353(15)
C4	B5	1.718(5)			

[1]1-X,+Y,3/2-Z

Table S16 Bond Angles for **2**×C_3H_6O.

Atom	Atom	Atom	Angle/˚	Atom	Atom	Atom	Angle/˚
C3	Si1	C3[1]	115.00(16)	B7	B3	B2	60.3(2)
C1[1]	Si1	C3[1]	114.85(12)	B7	B3	B4	108.4(3)
C1	Si1	C3	114.85(12)	B8	B3	B2	107.8(3)
C1[1]	Si1	C3	108.23(11)	B8	B3	B4	59.7(2)
C1	Si1	C3[1]	108.23(11)	B8	B3	B7	60.1(2)
C1	Si1	C1[1]	93.98(17)	C3	B4	B3	59.05(19)
C11	C2	C2[1]	119.43(15)	C3	B4	B5	59.08(19)
C1	C2	C2[1]	117.19(16)	C3	B4	B8	105.0(2)
C1	C2	C11	123.4(2)	C3	B4	B9	105.8(2)
C16	C11	C2	118.7(3)	B5	B4	B3	107.8(2)
C16	C11	C12	118.7(3)	B8	B4	B3	59.4(2)
C12	C11	C2	122.6(3)	B8	B4	B5	107.3(3)
C11	C16	C15	120.1(4)	B9	B4	B3	107.6(3)
C16	C15	C14	119.5(4)	B9	B4	B5	60.1(2)
C13	C14	C15	120.5(4)	B9	B4	B8	59.7(2)
C14	C13	C12	119.9(4)	C3	B5	B1	58.76(18)
C13	C12	C11	121.3(4)	C3	B5	B4	59.75(19)
C10	C5	C1	119.5(3)	C3	B5	B9	105.9(3)
C6	C5	C10	117.1(3)	C4	B5	C3	103.1(3)

C6	C5	C1	123.4(3)	C4	B5	B1	58.7(2)
C9	C10	C5	120.8(4)	C4	B5	B4	105.4(3)
C8	C9	C10	120.4(4)	C4	B5	B9	59.1(2)
C7	C8	C9	119.5(4)	B4	B5	B1	108.0(3)
C8	C7	C6	120.6(4)	B9	B5	B1	107.7(3)
C7	C6	C5	121.7(4)	B9	B5	B4	59.6(2)
B1	C3	Si1	124.8(2)	C4	B6	B1	59.0(2)
B1	C3	B2	61.7(2)	C4	B6	B2	105.9(3)
B1	C3	B3	111.6(2)	C4	B6	B7	105.6(3)
B1	C3	B4	111.5(2)	C4	B6	B10	58.9(2)
B1	C3	B5	61.7(2)	B1	B6	B2	60.1(2)
B2	C3	Si1	121.4(2)	B1	B6	B7	108.4(2)
B2	C3	B3	61.80(19)	B1	B6	B10	107.9(3)
B2	C3	B4	112.3(2)	B2	B6	B7	60.6(2)
B2	C3	B5	112.3(2)	B2	B6	B10	108.5(3)
B3	C3	Si1	115.89(19)	B10	B6	B7	60.0(2)
B3	C3	B4	61.6(2)	B3	B7	B2	60.0(2)
B4	C3	Si1	114.69(18)	B6	B7	B2	59.6(2)
B5	C3	Si1	120.0(2)	B6	B7	B3	107.6(3)
B5	C3	B3	111.5(2)	B8	B7	B2	107.1(3)
B5	C3	B4	61.2(2)	B8	B7	B3	59.5(2)
B1	C4	B5	61.9(2)	B8	B7	B6	107.1(3)
B1	C4	B6	61.7(2)	B8	B7	B10	59.7(2)
B1	C4	B9	112.3(3)	B10	B7	B2	107.7(3)
B6	C4	B5	112.7(3)	B10	B7	B3	107.8(3)
B6	C4	B9	112.9(3)	B10	B7	B6	60.0(2)
B9	C4	B5	61.6(2)	B3	B8	B4	60.9(2)
B10	C4	B1	112.7(3)	B3	B8	B7	60.4(2)
B10	C4	B5	113.1(3)	B3	B8	B10	108.8(3)
B10	C4	B6	62.0(2)	B4	B8	B7	109.5(3)
B10	C4	B9	62.5(2)	B9	B8	B3	109.1(3)
C2	C1	Si1	105.79(19)	B9	B8	B4	60.1(2)
C2	C1	C5	123.5(2)	B9	B8	B7	109.5(3)
C5	C1	Si1	129.98(18)	B9	B8	B10	61.1(2)
C3	B1	B2	59.35(18)	B10	B8	B4	109.4(3)
C3	B1	B5	59.54(18)	B10	B8	B7	60.2(2)
C3	B1	B6	106.4(3)	C4	B9	B4	106.1(3)
C4	B1	C3	104.2(2)	C4	B9	B5	59.3(2)
C4	B1	B2	106.1(3)	C4	B9	B8	104.8(3)
C4	B1	B5	59.4(2)	C4	B9	B10	58.5(2)
C4	B1	B6	59.3(2)	B4	B9	B5	60.3(2)
B2	B1	B5	108.7(3)	B4	B9	B10	108.4(3)
B6	B1	B2	60.1(2)	B5	B9	B10	107.9(3)

B6	B1	B5	108.7(3)	B8	B9	B4	60.3(2)
C3	B2	B1	58.95(18)	B8	B9	B5	107.9(3)
C3	B2	B3	59.44(18)	B8	B9	B10	59.7(2)
C3	B2	B6	105.9(3)	C4	B10	B6	59.2(2)
C3	B2	B7	106.0(2)	C4	B10	B7	105.9(3)
B1	B2	B3	107.5(3)	C4	B10	B8	104.8(3)
B1	B2	B6	59.9(2)	C4	B10	B9	59.0(2)
B1	B2	B7	107.5(3)	B6	B10	B9	107.8(3)
B3	B2	B7	59.6(2)	B7	B10	B6	60.0(2)
B6	B2	B3	107.4(3)	B7	B10	B9	107.9(3)
B6	B2	B7	59.7(2)	B8	B10	B6	107.4(3)
C3	B3	B2	58.76(19)	B8	B10	B7	60.1(2)
C3	B3	B4	59.40(19)	B8	B10	B9	59.2(2)
C3	B3	B7	106.1(3)	O1	C17	C19	121.9(11)
C3	B3	B8	105.5(3)	O1	C17	C18	124.7(15)
B2	B3	B4	108.0(3)	C18	C17	C19	113.4(12)

[1]1-X,+Y,3/2-Z

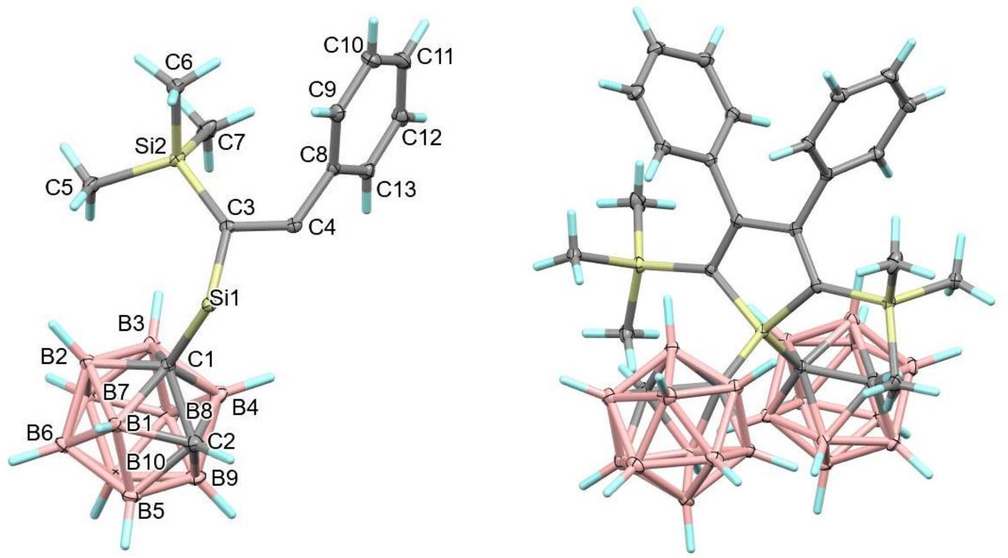

Figure S63 ORTEP view of an asymmetric unit of **4** (displacement ellipsoids are drawn at the 30% probability level) and capped sticks presentation of the symmetry generated molecule.

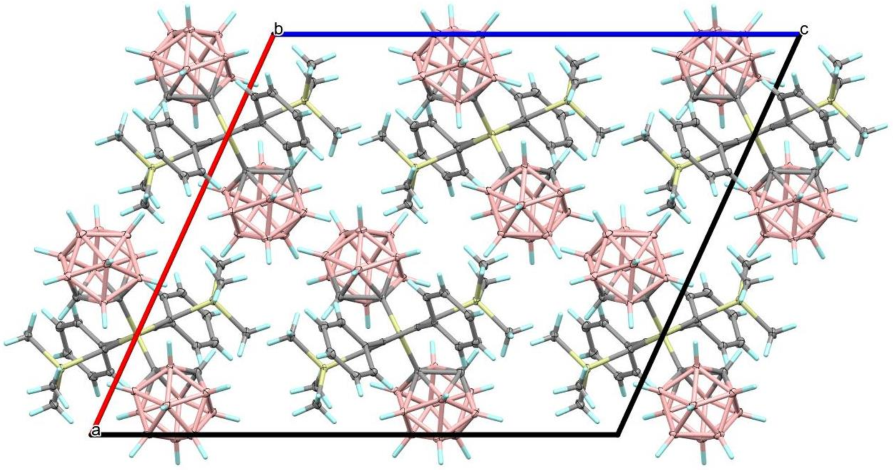

Figure S64 The molecular packing of **4** showing along the **b** axis.

Experimental

Crystal data: C26 H50 B20 Si3, *Fwt.*: 663.13, colourless, block, size: 0.500 x 0.50 x 0.30 mm, monoclinic, space group *I* 1 2/a 1, *a* = 17.4814(6)Å, *b* = 11.4884(3)Å, *c* = 20.882(2)Å, α = 90°, β = 114.467(6)°, γ = 90°, *V* = 3817.2(4)Å^3, *T* = 103(2)K, *Z*= 4, Z'=1, *F*(000) = 1392, D_x = 1.154 Mg/m^3, μ= 0.146mm^{-1}.

A crystal of LSQDATA_SB13 was mounted on a fibre. Cell parameters were determined by least-squares using 90512 (3.415 ≤ θ ≤ 27.465°) reflections.

Intensity data were collected on a Rigaku RAXIS-RAPID II diffractometer (graphite monochromator; ok-*K*α radiation, λ = 0.71075Å) at 103(2) K in the range 3.414 ≤ θ ≤ 26.023°. A total of 106541 reflections were collected of which 106541were unique [*R*(int) = 4.80, *R(*σ) = 0.0596]; intensities of 80079 reflections were greater than 2σ(*I*). Completeness to θ = 0.998. A numerical absorption correction was applied to the data (the minimum and maximum transmission factors were 0.978649 and 0.988687).

The structure was solved by direct methods (and subsequent difference syntheses).

Anisotropic full-matrix least-squares refinement on F^2 for all non-hydrogen atoms yielded R_1 = 0.0473 and wR^2 = 0.1077 for 1332 [*I*>2σ(*I*)] and R_1 = 0.0723 and wR^2 = 0.1154 for all (106541) intensity data, (number of parameters = 226, goodness-of-fit = 1.032, the maximum and mean shift/esd is 0.000 and 0.000).

The maximum and minimum residual electron density in the final difference map was 0.35 and -0.29e.Å^{-3}.

The weighting scheme applied was $w = 1/[\sigma^2(F_o^2)+(0.0573P)^2+1.1345P]$ where $P = (F_o^2+2F_c^2)/3$.

Hydrogen atomic positions were calculated from assumed geometries. Hydrogen atoms were included in structure factor calculations, but they were not refined. The isotropic displacement parameters of the hydrogen atoms were approximated from the *U*(eq) value of the atom they were bonded to.

Table S17 Crystal data and structure refinement for **4.**

Number	**4**
CCDC	2401434
Empirical formula	C_{26} H_{50} B_{20} Si_3
Formula weight	663.13
Temperature	103.15
Radiation and wavelength	Mok-Kα,<br>λ =0.71075Å
Crystal system	monoclinic
Space group	*I* 1 2/a 1
Unit cell dimensions	*a* =17.4814(6)Å<br>*b* =11.4884(3)Å<br>*c* =20.882(2)Å<br>α =90°<br>β =114.467(6)°<br>γ=90°
Volume	3817.2(4)$Å^3$
Z, Z'	4, 1
Density (calculated)	1.154 Mg/m^3
Absorption coefficient, [illegible]	0.146 mm^{-1}
F(000)	1392
Crystal colour	colourless
Crystal description	block
Crystal size	0.50 x 0.50 x 0.30 mm
Absorption correction	numerical
Max. and min. transmission	0.978649 and 0.988687
θ–range for data collection	3.414 ≤ θ ≤ 26.023°
Index ranges	-21 ≤ *h* ≤ 21;<br>-14 ≤ *k* ≤ 14;<br>-25 ≤ *l* ≤ 25
Reflections collected	106541
Completeness to 2θ	0.998
Independent reflections	106541 [*R*(int) =4.80]
Reflections *I*>2θ(*I*)	80079
Data / restraints / parameters	106541 /0 /226
Goodness-of-fit on F^2	1.032
Final *R* indices [*I*>2θ(*I*)]	R_1 =0.0473,<br>wR^2 =0.1077
R indices (all data)	R_1 =0.0723,<br>wR^2 =0.1154

Max. and mean shift/esd	0.000;0.000
Largest diff. peak and hole	0.35 and -0.29 e.Å^{-3}

Table S18 Bond Lengths for **4.**

Si1-C1	1.925(2)	Si1-C1#1	1.925(2)
Si1-C3#1	1.870(2)	Si1-C3	1.871(2)
Si2-C3	1.903(2)	Si2-C5	1.872(2)
Si2-C6	1.875(2)	Si2-C7	1.862(2)
C1-C2	1.684(3)	C1-B1	1.716(3)
C1-B2	1.721(3)	C1-B3	1.720(3)
C1-B4	1.740(3)	C2-B1	1.723(3)
C2-B4	1.725(3)	C2-B5	1.728(3)
C2-B9	1.715(4)	C3-C4	1.360(3)
C4-C4#1	1.515(4)	C4-C8	1.497(3)
C8-C9	1.385(3)	C8-C13	1.393(3)
C9-C10	1.393(3)	C10-C11	1.376(3)
C11-C12	1.383(3)	C12-C13	1.386(3)
B1-B2	1.758(3)	B1-B5	1.759(3)
B1-B6	1.745(3)	B2-B3	1.766(3)
B2-B6	1.778(3)	B2-B7	1.770(4)
B3-B4	1.763(3)	B3-B7	1.767(3)
B3-B8	1.766(4)	B4-B8	1.774(4)
B4-B9	1.782(4)	B5-B6	1.777(4)
B5-B9	1.782(4)	B5-B10	1.777(4)
B6-B7	1.781(4)	B6-B10	1.789(4)
B7-B8	1.790(4)	B7-B10	1.785(4)
B8-B9	1.779(4)	B8-B10	1.789(4)
B9-B10	1.786(4)		

Table S19 Bond Angles for **4**.

C1-Si1-C1#1	115.4(1)	C3-Si1-C1	111.36(9)
C3-Si1-C1#1	109.70(9)	C3#1-Si1-C1#1	111.35(9)
C3#1-Si1-C1	109.70(9)	C3#1-Si1-C3	98.0(1)
C5-Si2-C3	111.6(1)	C5-Si2-C6	108.5(1)
C6-Si2-C3	110.6(1)	C7-Si2-C3	113.2(1)
C7-Si2-C5	105.3(1)	C7-Si2-C6	107.5(1)
C2-C1-Si1	117.7(1)	C2-C1-B1	60.9(1)
C2-C1-B2	110.1(2)	C2-C1-B3	108.9(2)
C2-C1-B4	60.5(1)	B1-C1-Si1	123.6(2)
B1-C1-B2	61.5(1)	B1-C1-B3	111.1(2)
B1-C1-B4	111.7(2)	B2-C1-Si1	125.6(1)
B2-C1-B4	112.4(2)	B3-C1-Si1	119.9(1)
B3-C1-B2	61.7(1)	B3-C1-B4	61.3(1)
B4-C1-Si1	112.7(1)	C1-C2-B1	60.5(1)
C1-C2-B4	61.3(1)	C1-C2-B5	110.3(2)
C1-C2-B9	111.1(2)	B1-C2-B4	112.0(2)
B1-C2-B5	61.3(1)	B4-C2-B5	113.3(2)
B9-C2-B1	112.1(2)	B9-C2-B4	62.4(2)
B9-C2-B5	62.3(2)	Si1-C3-Si2	134.8(1)
C4-C3-Si1	102.2(2)	C4-C3-Si2	122.9(2)
C3-C4-C4#1	118.7(1)	C3-C4-C8	123.7(2)
C8-C4-C4#1	117.6(1)	C9-C8-C4	119.5(2)
C9-C8-C13	119.2(2)	C13-C8-C4	121.3(2)
C8-C9-C10	120.5(2)	C11-C10-C9	119.9(2)
C10-C11-C12	120.0(2)	C11-C12-C13	120.3(2)
C12-C13-C8	120.0(2)	C1-B1-C2	58.6(1)
C1-B1-B2	59.4(1)	C1-B1-B5	107.4(2)
C1-B1-B6	107.6(2)	C2-B1-B2	106.6(2)
C2-B1-B5	59.5(1)	C2-B1-B6	107.2(2)
B2-B1-B5	109.8(2)	B6-B1-B2	61.0(1)

B6-B1-B5	61.0(2)	C1-B2-B1	59.1(1)
C1-B2-B3	59.1(1)	C1-B2-B6	105.9(2)
C1-B2-B7	106.2(2)	B1-B2-B3	107.1(2)
B1-B2-B6	59.1(1)	B1-B2-B7	107.1(2)
B3-B2-B6	107.8(2)	B3-B2-B7	60.0(1)
B7-B2-B6	60.3(1)	C1-B3-B2	59.2(1)
C1-B3-B4	59.9(1)	C1-B3-B7	106.4(2)
C1-B3-B8	106.9(2)	B2-B3-B7	60.2(1)
B2-B3-B8	109.2(2)	B4-B3-B2	109.1(2)
B4-B3-B7	109.3(2)	B4-B3-B8	60.4(2)
B8-B3-B7	60.9(2)	C1-B4-B3	58.8(1)
C1-B4-B8	105.7(2)	C1-B4-B9	105.5(2)
C2-B4-C1	58.2(1)	C2-B4-B3	105.1(2)
C2-B4-B8	105.3(2)	C2-B4-B9	58.5(1)
B3-B4-B8	59.9(2)	B3-B4-B9	107.5(2)
B8-B4-B9	60.0(2)	C2-B5-B1	59.2(1)
C2-B5-B6	105.6(2)	C2-B5-B9	58.5(1)
C2-B5-B10	105.4(2)	B1-B5-B6	59.1(1)
B1-B5-B9	107.3(2)	B1-B5-B10	107.3(2)
B6-B5-B9	108.4(2)	B10-B5-B6	60.5(2)
B10-B5-B9	60.3(2)	B1-B6-B2	59.9(1)
B1-B6-B5	59.9(1)	B1-B6-B7	107.2(2)
B1-B6-B10	107.3(2)	B2-B6-B7	59.6(1)
B2-B6-B10	107.7(2)	B5-B6-B2	108.0(2)
B5-B6-B7	107.7(2)	B5-B6-B10	59.8(2)
B7-B6-B10	60.0(2)	B2-B7-B6	60.1(1)
B2-B7-B8	107.9(2)	B2-B7-B10	108.3(2)
B3-B7-B2	59.9(1)	B3-B7-B6	107.7(2)
B3-B7-B8	59.5(1)	B3-B7-B10	107.5(2)
B6-B7-B8	108.2(2)	B6-B7-B10	60.2(2)
B10-B7-B8	60.1(2)	B3-B8-B4	59.8(1)

B3-B8-B7	59.6(1)	B3-B8-B9	107.5(2)
B3-B8-B10	107.4(2)	B4-B8-B7	107.8(2)
B4-B8-B9	60.2(2)	B4-B8-B10	108.2(2)
B9-B8-B7	107.8(2)	B9-B8-B10	60.1(2)
B10-B8-B7	59.8(2)	C2-B9-B4	59.1(1)
C2-B9-B5	59.2(1)	C2-B9-B8	105.5(2)
C2-B9-B10	105.6(2)	B4-B9-B5	108.1(2)
B4-B9-B10	108.0(2)	B5-B9-B10	59.7(2)
B8-B9-B4	59.8(2)	B8-B9-B5	107.9(2)
B8-B9-B10	60.2(2)	B5-B10-B6	59.8(2)
B5-B10-B7	107.6(2)	B5-B10-B8	107.7(2)
B5-B10-B9	60.0(2)	B7-B10-B6	59.8(2)
B7-B10-B8	60.1(2)	B7-B10-B9	107.6(2)
B8-B10-B6	107.8(2)	B9-B10-B6	107.7(2)
B9-B10-B8	59.7(2)		

Symmetry codes to generate equivalent atoms:
1. [2_556] -x+1/2,y,-z+1

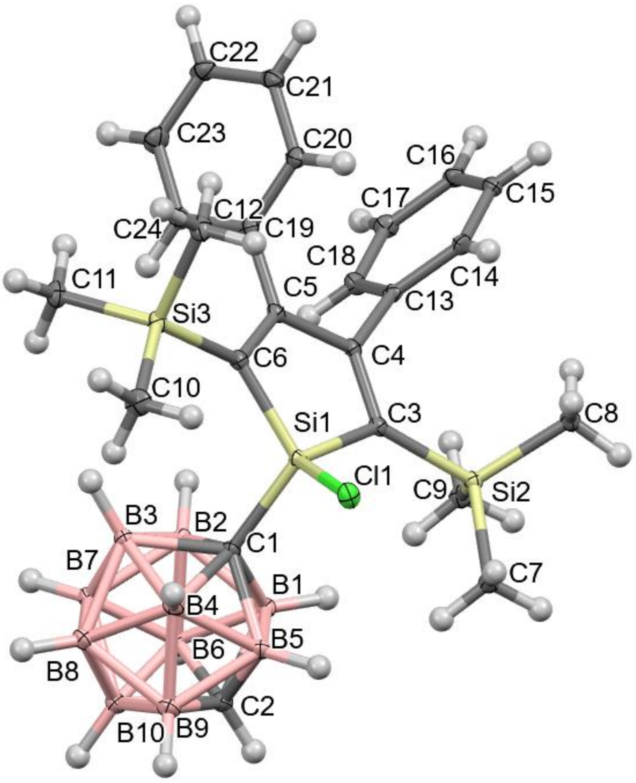

Figure S65 ORTEP view of an asymmetric unit of **5** (displacement ellipsoids are drawn at the 30% probability level).

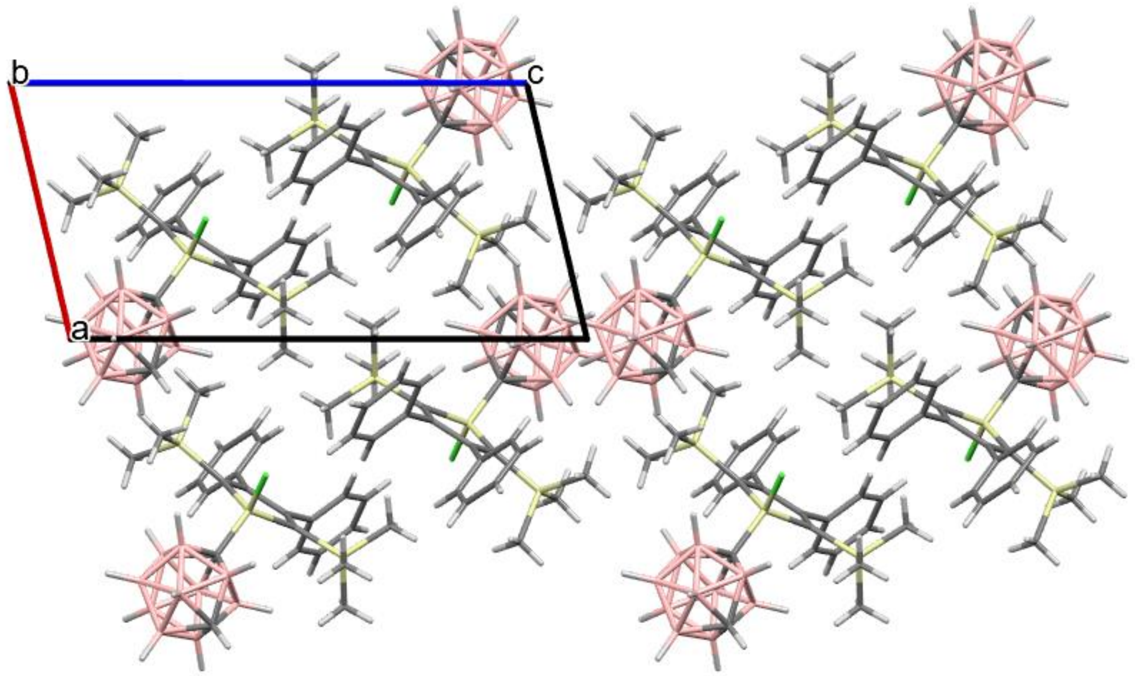

Figure S66 The molecular packing of **5** is shown along the **b-**axis.

Experimental

Single colourless plate-shaped crystals of **5** were used as supplied. A suitable crystal with dimensions 0.20 × 0.12 × 0.06 mm^3 was selected and mounted on a STOE STADIVARI diffractometer. The crystal was kept at a steady T = 100 K during data collection.

The structure was solved with the ShelXT[57] solution program using dual methods and Olex2 1.5[58] as the graphical interface. The model was refined with ShelXL 2019/3[59] using full matrix least squares minimisation on $\boldsymbol{F}^2$. Data were measured using rotation method, ω scans with Cu K_α radiation (λ=1.54186). The final completeness is 98.70 %.

All non-hydrogen atoms were refined anisotropically. Hydrogen atom positions were calculated geometrically and refined using the riding model. Most hydrogen atom positions were calculated geometrically and refined using the riding model, but some hydrogen atoms were refined freely.

There is a single formula unit in the asymmetric unit, represented by the reported sum formula. In other words, Z is 2, and Z' is 1.

Crystallography data including structural factors (fcf) were deposited in the Cambridge Crystallographic Data Centre (CCDC) number of 2402095.

Table S20 Crystal data and structure refinement for **5.**

Identification code	**5**
Empirical formula	$C_{24}H_{39}B_{10}ClSi_3$
Formula weight	555.37
Temperature/K	100
Crystal system	triclinic
Space group	P-1
a/Å	9.3159(3)
b/Å	11.2414(4)
c/Å	16.8595(6)
α/°	82.506(3)
β/°	74.949(3)
γ/°	66.147(2)
Volume/Å^3	1558.74(10)
Z	2
ρ_{calc}g/cm^3	1.183
μ/mm^{-1}	2.277
F(000)	584.0
Crystal size/mm^3	0.2 × 0.123 × 0.06
Radiation	Cu Kα (λ = 1.54186)

2Θ range for data collection/°	5.43 to 141.104
Index ranges	-10 ≤ *h* ≤ 11, -13 ≤ *k* ≤ 10, -19 ≤ *l* ≤ 20
Reflections collected	14679
Independent reflections	5766 [R_{int} = 0.0202, R_{sigma} = 0.0181]
Data/restraints/parameters	5766/0/352
Goodness-of-fit on F^2	1.043
Final R indexes [I>=2σ (I)]	R_1 = 0.0371, wR_2 = 0.0997
Final R indexes [all data]	R_1 = 0.0391, wR_2 = 0.1017
Largest diff. peak/hole / e Å^{-3}	0.47/-0.35

Table S21 Bond Lengths for **5**.

Atom	Atom	Length/Å	Atom	Atom	Length/Å
C1	B1	1.709(2)	B3	B7	1.774(3)
C1	Si1	1.9031(15)	B3	B8	1.772(2)
C1	B2	1.732(2)	C4	C5	1.532(2)
C1	B3	1.734(2)	C4	C13	1.500(2)
C1	B4	1.730(2)	B4	B5	1.769(3)
C1	B5	1.707(2)	B4	B8	1.785(3)
B1	C2	1.692(2)	B4	B9	1.775(3)
B1	B2	1.763(2)	C5	C6	1.359(2)
B1	B5	1.785(3)	C5	C19	1.489(2)
B1	B6	1.764(2)	B5	B9	1.768(3)
Cl1	Si1	2.0541(5)	B6	B7	1.775(3)
Si1	C3	1.8613(15)	B6	B10	1.785(3)
Si1	C6	1.8561(15)	B7	B8	1.785(3)
C2	B5	1.698(3)	B7	B10	1.775(3)
C2	B6	1.716(2)	B8	B9	1.782(3)
C2	B9	1.714(3)	B8	B10	1.776(3)
C2	B10	1.713(2)	B9	B10	1.782(3)
B2	B3	1.780(3)	C13	C14	1.391(2)
B2	B6	1.776(2)	C13	C18	1.397(2)
B2	B7	1.785(2)	C14	C15	1.393(2)
Si2	C3	1.8854(15)	C15	C16	1.392(2)
Si2	C7	1.8799(16)	C16	C17	1.387(2)
Si2	C8	1.8724(16)	C17	C18	1.389(2)
Si2	C9	1.8682(17)	C19	C20	1.401(2)
C3	C4	1.358(2)	C19	C24	1.392(2)
Si3	C6	1.8875(15)	C20	C21	1.387(2)
Si3	C10	1.8654(17)	C21	C22	1.385(3)
Si3	C11	1.8653(17)	C22	C23	1.385(3)
Si3	C12	1.8704(16)	C23	C24	1.393(2)

B3	B4	1.780(3)

Table S22 Bond Angles for **5**.

Atom	Atom	Atom	Angle/˚	Atom	Atom	Atom	Angle/˚
B1	C1	Si1	118.37(10)	B5	B4	B9	59.83(10)
B1	C1	B2	61.66(10)	B9	B4	B3	107.52(14)
B1	C1	B3	112.17(12)	B9	B4	B8	60.08(11)
B1	C1	B4	113.56(12)	C6	C5	C4	118.05(13)
B2	C1	Si1	117.03(10)	C6	C5	C19	125.19(13)
B2	C1	B3	61.80(10)	C19	C5	C4	116.76(12)
B3	C1	Si1	118.75(10)	C1	B5	B1	58.54(9)
B4	C1	Si1	119.66(11)	C1	B5	B4	59.65(9)
B4	C1	B2	113.24(12)	C1	B5	B9	106.19(12)
B4	C1	B3	61.85(10)	C2	B5	C1	101.89(12)
B5	C1	B1	63.01(10)	C2	B5	B1	58.08(10)
B5	C1	Si1	119.78(10)	C2	B5	B4	105.27(13)
B5	C1	B2	113.64(12)	C2	B5	B9	59.26(11)
B5	C1	B3	112.53(11)	B4	B5	B1	108.06(12)
B5	C1	B4	61.96(10)	B9	B5	B1	107.97(13)
C1	B1	B2	59.81(9)	B9	B5	B4	60.24(11)
C1	B1	B5	58.46(9)	B1	B6	B2	59.76(10)
C1	B1	B6	106.53(12)	B1	B6	B7	107.83(13)
C2	B1	C1	102.05(12)	B1	B6	B10	107.43(13)
C2	B1	B2	105.68(12)	C2	B6	B1	58.18(10)
C2	B1	B5	58.38(10)	C2	B6	B2	104.15(13)
C2	B1	B6	59.49(10)	C2	B6	B7	104.33(14)
B2	B1	B5	108.42(12)	C2	B6	B10	58.56(10)
B2	B1	B6	60.45(10)	B2	B6	B10	107.92(13)
B6	B1	B5	108.55(13)	B7	B6	B2	60.35(10)
C1	Si1	Cl1	106.51(5)	B7	B6	B10	59.79(10)
C3	Si1	C1	113.90(7)	Si1	C6	Si3	131.41(8)
C3	Si1	Cl1	112.18(5)	C5	C6	Si1	102.94(11)
C6	Si1	C1	112.49(7)	C5	C6	Si3	125.15(12)
C6	Si1	Cl1	114.04(5)	B2	B7	B8	108.08(13)
C6	Si1	C3	97.85(7)	B3	B7	B2	60.03(10)
B1	C2	B5	63.54(10)	B3	B7	B6	107.78(12)
B1	C2	B6	62.33(10)	B3	B7	B8	59.74(10)
B1	C2	B9	115.01(13)	B3	B7	B10	107.41(13)
B1	C2	B10	114.28(13)	B6	B7	B2	59.84(10)
B5	C2	B6	115.13(13)	B6	B7	B8	108.34(13)
B5	C2	B9	62.39(11)	B10	B7	B2	107.97(12)
B5	C2	B10	114.36(14)	B10	B7	B6	60.37(11)

B9	C2	B6	114.90(13)	B10	B7	B8	59.86(11)
B10	C2	B6	62.73(11)	B3	B8	B4	60.05(10)
B10	C2	B9	62.65(12)	B3	B8	B7	59.81(10)
C1	B2	B1	58.53(9)	B3	B8	B9	107.53(12)
C1	B2	B3	59.17(9)	B3	B8	B10	107.40(13)
C1	B2	B6	105.02(12)	B7	B8	B4	107.99(12)
C1	B2	B7	105.24(12)	B9	B8	B4	59.67(11)
B1	B2	B3	107.48(12)	B9	B8	B7	107.88(14)
B1	B2	B6	59.79(10)	B10	B8	B4	107.74(13)
B1	B2	B7	107.43(12)	B10	B8	B7	59.77(11)
B3	B2	B7	59.67(10)	B10	B8	B9	60.10(11)
B6	B2	B3	107.49(12)	C2	B9	B4	104.33(12)
B6	B2	B7	59.82(10)	C2	B9	B5	58.35(10)
C7	Si2	C3	110.31(7)	C2	B9	B8	104.38(13)
C8	Si2	C3	111.55(7)	C2	B9	B10	58.65(11)
C8	Si2	C7	104.66(7)	B4	B9	B8	60.25(11)
C9	Si2	C3	109.00(7)	B4	B9	B10	107.95(13)
C9	Si2	C7	111.77(8)	B5	B9	B4	59.93(10)
C9	Si2	C8	109.52(8)	B5	B9	B8	107.98(13)
Si1	C3	Si2	131.40(8)	B5	B9	B10	107.73(14)
C4	C3	Si1	103.07(10)	B10	B9	B8	59.78(11)
C4	C3	Si2	125.53(11)	C2	B10	B6	58.71(10)
C10	Si3	C6	109.95(7)	C2	B10	B7	104.48(12)
C10	Si3	C12	107.07(8)	C2	B10	B8	104.69(13)
C11	Si3	C6	113.79(7)	C2	B10	B9	58.70(11)
C11	Si3	C10	109.21(8)	B7	B10	B6	59.84(10)
C11	Si3	C12	109.53(8)	B7	B10	B8	60.37(11)
C12	Si3	C6	107.06(7)	B7	B10	B9	108.38(14)
C1	B3	B2	59.02(9)	B8	B10	B6	108.32(13)
C1	B3	B4	58.95(9)	B8	B10	B9	60.12(11)
C1	B3	B7	105.61(12)	B9	B10	B6	108.32(13)
C1	B3	B8	105.69(12)	C14	C13	C4	121.54(13)
B2	B3	B4	108.56(12)	C14	C13	C18	118.85(14)
B7	B3	B2	60.30(10)	C18	C13	C4	119.33(13)
B7	B3	B4	108.73(13)	C13	C14	C15	120.67(14)
B8	B3	B2	108.87(13)	C16	C15	C14	120.01(14)
B8	B3	B4	60.34(10)	C17	C16	C15	119.62(14)
B8	B3	B7	60.46(11)	C16	C17	C18	120.28(15)
C3	C4	C5	117.72(13)	C17	C18	C13	120.55(14)
C3	C4	C13	126.53(13)	C20	C19	C5	121.10(14)
C13	C4	C5	115.75(12)	C24	C19	C5	119.75(14)
C1	B4	B3	59.20(9)	C24	C19	C20	119.06(14)
C1	B4	B5	58.39(9)	C21	C20	C19	120.26(16)

C1	B4	B8	105.32(13)	C22	C21	C20	120.21(16)
C1	B4	B9	104.90(13)	C21	C22	C23	120.06(16)
B3	B4	B8	59.62(10)	C22	C23	C24	119.99(16)
B5	B4	B3	107.47(13)	C19	C24	C23	120.41(15)
B5	B4	B8	107.77(14)				

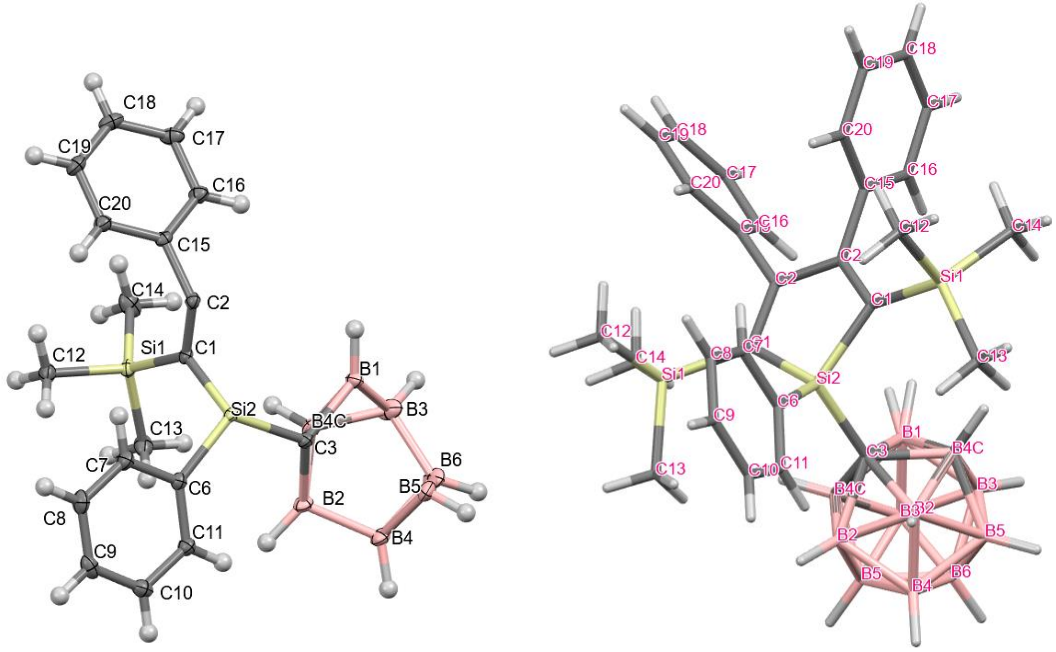

Figure S67 ORTEP view of an asymmetric unit of **6** (displacement ellipsoids are drawn at the 30% probability level) and capped sticks presentation of the symmetry generated molecule (+X,3/2-Y,+Z).

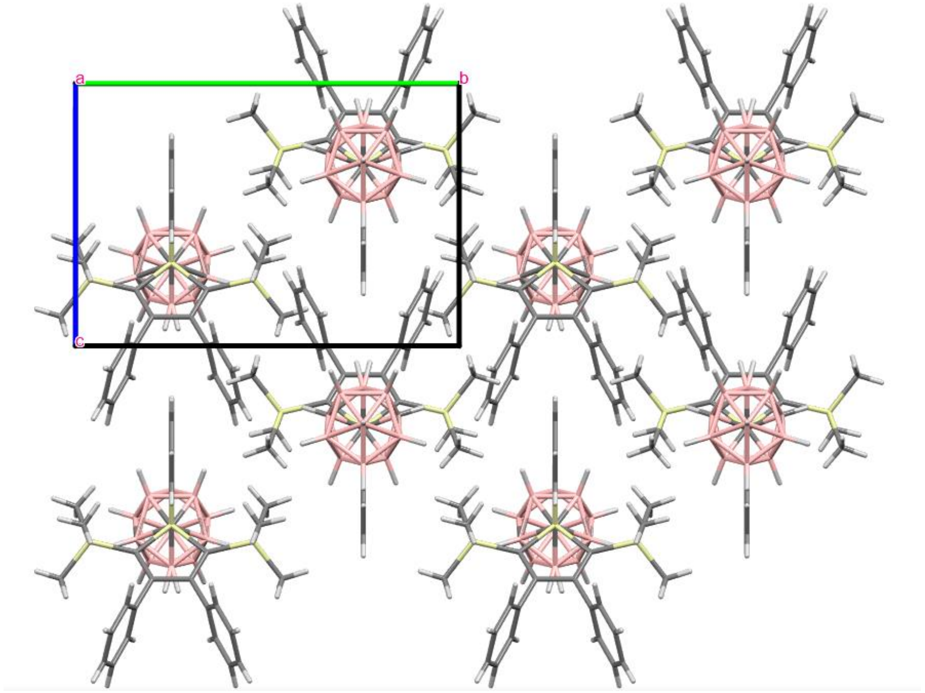

Figure S68 The molecular packing of **6** showing along the **a** axis

Experimental

Single colourless block-shaped crystals of **6** were used as supplied. A suitable crystal with dimensions 0.54 × 0.37 × 0.25 mm^3 was selected and mounted on a Bruker Venture D8 APEX-II CCD diffractometer. The crystal was kept at a steady T = 150.15 K during data collection. Data were measured using ϕ and ω scans with MoKα radiation. The final completeness is 99.60 %.

The structure was solved with the ShelXT 2018/2[57] solution program using direct methods and Olex2 1.5[58] as the graphical interface. The model was refined with ShelXL 2018/3[59] using full matrix least squares minimisation on $\boldsymbol{F^2}$.

B4C and C4B atoms are in the same position with a 50% probability.

All non-hydrogen atoms were refined anisotropically. Hydrogen atom positions were calculated geometrically and refined using the riding model.

The value of Z' is 0.5. This means that only half of the formula unit is present in the asymmetric unit, with the other half consisting of symmetry equivalent atoms.

The structure of **6** was validated by PLATON[60], and the publication materials were prepared using OLEX2, Mercury[61] softwares.

Crystallography data including structural factors (fcf) were deposited in the Cambridge Crystallographic Data Centre (CCDC) number of 2395433.

Table S23 Crystal data and structure refinement for **6.**

Identification code	**6**
Empirical formula	$C_{30}H_{43}B_{10}Si_3$
Formula weight	596.01
Temperature/K	150.15
Crystal system	monoclinic
Space group	$P2_1/m$
a/Å	11.0874(9)
b/Å	15.0263(12)
c/Å	11.3396(9)
α/°	90
β/°	114.989(3)
γ/°	90
Volume/Å^3	1712.4(2)
Z	2
ρ_{calc}g/cm^3	1.156
μ/mm^{-1}	0.160

F(000)	630.0
Crystal size/mm^3	0.54 × 0.37 × 0.254
Radiation	MoKα (λ = 0.71073)
2Θ range for data collection/°	3.962 to 52.744
Index ranges	-13 ≤ h ≤ 13, -18 ≤ k ≤ 18, -14 ≤ l ≤ 14
Reflections collected	44526
Independent reflections	3625 [R_{int} = 0.1424, R_{sigma} = 0.0535]
Data/restraints/parameters	3625/0/214
Goodness-of-fit on F^2	1.034
Final R indexes [I>=2σ (I)]	R_1 = 0.0436, wR_2 = 0.1146
Final R indexes [all data]	R_1 = 0.0555, wR_2 = 0.1231
Largest diff. peak/hole / e Å^{-3}	0.43/-0.43

Table S24 Bond Lengths for **6.**

Atom	Atom	Length/Å	Atom	Atom	Length/Å
Si1	C1	1.8799(17)	C7	C8	1.387(4)
Si1	C12	1.861(2)	C8	C9	1.385(4)
Si1	C13	1.8690(19)	C9	C10	1.380(4)
Si1	C14	1.862(2)	C10	C11	1.389(4)
Si2	C1	1.8689(17)	C15	C16	1.393(2)
Si2	C1^1	1.8689(17)	C15	C20	1.389(3)
Si2	C3	1.921(2)	C16	C17	1.388(3)
Si2	C6	1.878(2)	C17	C18	1.375(3)
C1	C2	1.355(2)	C18	C19	1.384(3)
C2	C2^1	1.513(3)	C19	C20	1.385(2)
C2	C15	1.493(2)	B1	B3	1.776(4)
C3	C4^1	1.687(2)	B1	B3^1	1.776(4)
C3	C4	1.687(2)	B2	B2^1	1.776(4)
C3	B1	1.734(3)	B2	B4	1.767(3)
C3	B2^1	1.714(3)	B2	B5^1	1.772(3)
C3	B2	1.714(3)	B3	B3^1	1.770(6)
C4	B1	1.735(3)	B3	B5^1	1.784(3)
C4	B2	1.740(3)	B3	B6	1.781(4)
C4	B3	1.724(3)	B4	B5	1.773(3)
C4	B5^1	1.731(3)	B4	B5^1	1.773(3)
C6	C7	1.399(4)	B4	B6	1.788(5)
C6	C11	1.393(3)	B5	B6	1.767(4)

^{1}+X,3/2-Y,+Z

Table 25 Bond Angles for **6.**

Atom	Atom	Atom	Angle/˚	Atom	Atom	Atom	Angle/˚
C12	Si1	C1	107.74(8)	C3	B1	C4	58.20(10)
C12	Si1	C13	106.90(9)	C3	B1	B3^{1}	105.15(16)
C12	Si1	C14	109.53(10)	C3	B1	B3	105.15(16)
C13	Si1	C1	110.68(8)	C4^{1}	B1	C4	104.08(19)
C14	Si1	C1	113.44(8)	C4^{1}	B1	B3	105.57(18)
C14	Si1	C13	108.36(11)	C4	B1	B3	58.83(13)
C1^{1}	Si2	C1	96.27(10)	C4^{1}	B1	B3^{1}	58.83(13)
C1^{1}	Si2	C3	109.53(7)	C4	B1	B3^{1}	105.57(18)
C1	Si2	C3	109.53(7)	B3^{1}	B1	B3	59.8(2)
C1^{1}	Si2	C6	112.17(7)	C3	B2	C4	58.46(10)
C1	Si2	C6	112.17(7)	C3	B2	B2^{1}	58.81(8)
C6	Si2	C3	115.51(10)	C3	B2	B4	105.57(14)
Si2	C1	Si1	129.37(9)	C3	B2	B5^{1}	105.64(15)
C2	C1	Si1	125.35(13)	C4	B2	B2^{1}	106.00(9)
C2	C1	Si2	103.86(12)	C4	B2	B4	106.19(16)
C1	C2	C2^{1}	117.96(10)	C4	B2	B5^{1}	59.04(12)
C1	C2	C15	124.92(15)	B4	B2	B2^{1}	59.83(8)
C15	C2	C2^{1}	117.01(9)	B4	B2	B5^{1}	60.15(14)
C4	C3	Si2	120.64(10)	B5^{1}	B2	B2^{1}	107.75(12)
C4^{1}	C3	Si2	120.64(10)	C4	B3	B1	59.40(12)
C4^{1}	C3	C4	108.33(19)	C4	B3	B3^{1}	106.25(12)
C4^{1}	C3	B1	60.92(10)	C4	B3	B5^{1}	59.10(13)
C4	C3	B1	60.92(10)	C4	B3	B6	105.70(17)
C4^{1}	C3	B2^{1}	61.54(11)	B1	B3	B5^{1}	107.90(16)
C4	C3	B2	61.54(11)	B1	B3	B6	108.08(18)
C4^{1}	C3	B2	111.30(16)	B3^{1}	B3	B1	60.10(11)
C4	C3	B2^{1}	111.30(16)	B3^{1}	B3	B5^{1}	107.72(13)
B1	C3	Si2	116.64(15)	B3^{1}	B3	B6	60.20(11)
B2^{1}	C3	Si2	120.17(13)	B6	B3	B5^{1}	59.42(14)
B2	C3	Si2	120.17(13)	B2	B4	B2^{1}	60.34(17)
B2	C3	B1	113.17(16)	B2^{1}	B4	B5^{1}	108.09(18)
B2^{1}	C3	B1	113.17(16)	B2	B4	B5	108.09(18)
B2^{1}	C3	B2	62.39(16)	B2	B4	B5^{1}	60.08(12)
C3	C4	B1	60.88(12)	B2^{1}	B4	B5	60.08(12)
C3	C4	B2	60.00(12)	B2^{1}	B4	B6	107.64(17)
C3	C4	B3	109.58(17)	B2	B4	B6	107.64(17)
C3	C4	B5^{1}	108.72(15)	B5	B4	B5^{1}	107.3(2)
B1	C4	B2	111.85(15)	B5^{1}	B4	B6	59.49(13)
B3	C4	B1	61.77(15)	B5	B4	B6	59.49(13)
B3	C4	B2	112.30(15)	C4^{1}	B5	B2^{1}	59.56(12)

B3	C4	B5[1]	62.17(14)	C4[1]	B5	B3[1]	58.74(13)
B5[1]	C4	B1	112.29(18)	C4[1]	B5	B4	106.31(17)
B5[1]	C4	B2	61.39(12)	C4[1]	B5	B6	106.04(18)
C7	C6	Si2	112.93(18)	B2[1]	B5	B3[1]	108.03(15)
C11	C6	Si2	129.2(2)	B2[1]	B5	B4	59.78(14)
C11	C6	C7	117.8(2)	B4	B5	B3[1]	108.64(19)
C8	C7	C6	121.6(2)	B6	B5	B2[1]	108.33(18)
C9	C8	C7	119.5(3)	B6	B5	B3[1]	60.21(17)
C10	C9	C8	119.9(2)	B6	B5	B4	60.66(16)
C9	C10	C11	120.6(2)	B3[1]	B6	B3	59.6(2)
C10	C11	C6	120.7(3)	B3	B6	B4	108.1(2)
C16	C15	C2	120.20(15)	B3[1]	B6	B4	108.1(2)
C20	C15	C2	120.75(15)	B5	B6	B3	108.0(2)
C20	C15	C16	119.02(15)	B5	B6	B3[1]	60.38(14)
C17	C16	C15	120.05(18)	B5[1]	B6	B3	60.37(14)
C18	C17	C16	120.40(18)	B5[1]	B6	B3[1]	108.0(2)
C17	C18	C19	120.04(16)	B5[1]	B6	B4	59.85(13)
C18	C19	C20	119.88(18)	B5	B6	B4	59.85(13)
C19	C20	C15	120.61(17)	B5	B6	B5[1]	107.9(2)
C3	B1	C4[1]	58.20(10)				

[1]+X,3/2-Y,+Z

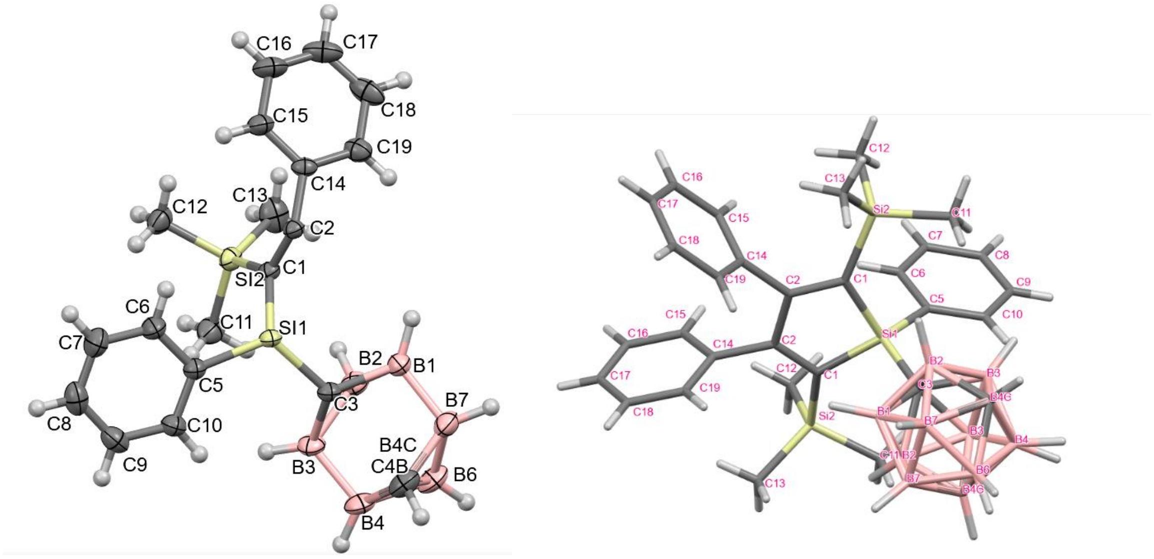

Figure S69 ORTEP view of an asymmetric unit of **7** (displacement ellipsoids are drawn at the 30% probability level) and capped sticks presentation of the symmetry generated molecule (+X,3/2-Y,+Z)

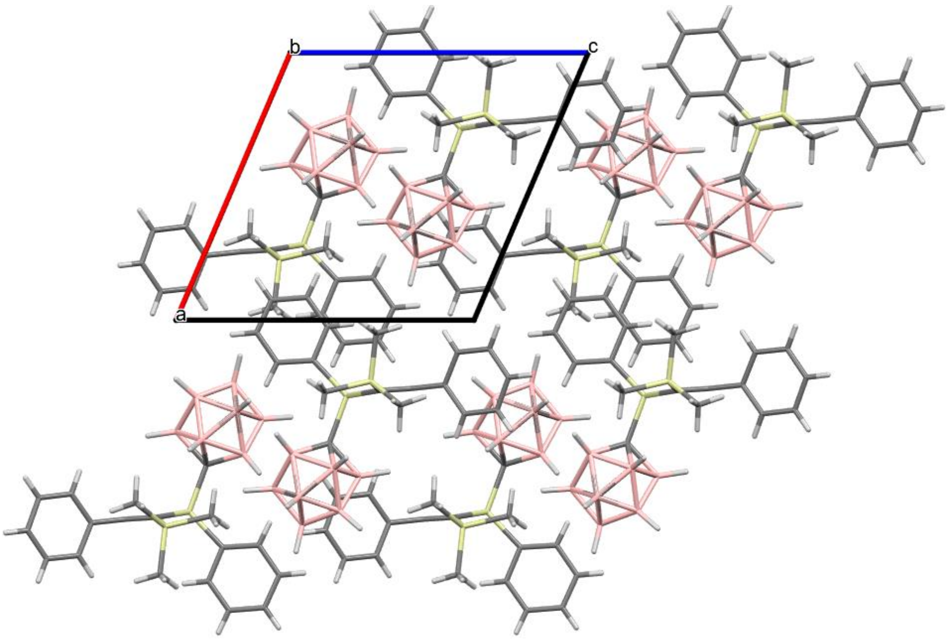

Figure S70 The molecular packing of **7** showing along the **b** axis

Experimental

Single colourless block-shaped crystals of **7** were used as supplied. A suitable crystal with dimensions 0.54 × 0.37 × 0.25 mm^3 was selected and mounted on a Bruker Venture D8 APEX-II CCD diffractometer. The crystal was kept at a steady *T* = 293.(2) K during data collection.

The structure was solved with the ShelXT[57] solution program using iterative methods and Olex2 1.5[58] as the graphical interface. The model was refined with ShelXL 2018/3[59] using full matrix least squares minimisation on $\boldsymbol{F^2}$. Data were measured using ϕ and ω scans with MoKα radiation. The final completeness is 99.80 %.

B4C and C4B atoms are in the same position with 50% probability.

All non-hydrogen atoms were refined anisotropically. Hydrogen atom positions were calculated geometrically and refined using the riding model.

The value of Z' is 0.5. This means that only half of the formula unit is present in the asymmetric unit, with the other half consisting of symmetry equivalent atoms.

The structure of **7** was validated by PLATON[60], and the publication materials were prepared using OLEX2, Mercury[61] softwares.

Crystallography data including structural factors (fcf) were deposited in the Cambridge Crystallographic Data Centre (CCDC) number of 2395434.

Table S26 Crystal data and structure refinement for **7.**

Identification code	**7**
Empirical formula	$C_{30}H_{43}B_{10}Si_3$
Formula weight	596.01
Temperature/K	293.15
Crystal system	monoclinic
Space group	$P2_1/m$
a/Å	11.0917(18)
b/Å	15.219(2)
c/Å	11.3723(19)
α/°	90
β/°	113.161(7)
γ/°	90
Volume/Å^3	1764.9(5)
Z	2
ρ_{calc}g/cm^3	1.122
μ/mm^{-1}	0.155
F(000)	630.0
Crystal size/mm^3	0.54 × 0.37 × 0.254
Radiation	MoKα (λ = 0.71073)
2Θ range for data collection/°	3.994 to 50.698
Index ranges	$-13 \le h \le 13$, $-18 \le k \le 18$, $-13 \le l \le 13$
Reflections collected	31650
Independent reflections	3354 [R_{int} = 0.1531, R_{sigma} = 0.0660]
Data/restraints/parameters	3354/0/209
Goodness-of-fit on F^2	1.023
Final R indexes [I>=2σ (I)]	R_1 = 0.0597, wR_2 = 0.1356
Final R indexes [all data]	R_1 = 0.0890, wR_2 = 0.1546
Largest diff. peak/hole / e Å^{-3}	0.32/-0.33

Table S27 Bond Lengths for **7**

**Atom**	**Atom**	**Length/Å**	**Atom**	**Atom**	**Length/Å**
Si1	C1	1.860(3)	C6	C7	1.362(7)
Si1	C1^1	1.860(3)	C7	C8	1.363(7)
Si1	C3	1.919(4)	C8	C9	1.359(7)
Si1	C5	1.860(4)	C9	C10	1.385(7)
Si2	C1	1.876(3)	C14	C15	1.384(4)
Si2	C11	1.859(3)	C14	C19	1.371(4)
Si2	C12	1.854(3)	C15	C16	1.372(4)
Si2	C13	1.843(4)	C16	C17	1.340(6)
C1	C2	1.344(3)	C17	C18	1.358(6)

Table S27 Bond Lengths for **7**

Atom	Atom	Length/Å	Atom	Atom	Length/Å
C2	C2[1]	1.512(5)	C18	C19	1.399(5)
C2	C14	1.491(3)	B1	B2[1]	1.747(5)
C3	B1	1.709(7)	B1	B2	1.747(5)
C3	B2	1.715(4)	B1	B7	1.758(6)
C3	B2[1]	1.715(4)	B1	B7[1]	1.758(6)
C3	B3[1]	1.708(5)	B2	B3	1.767(5)
C3	B3	1.708(4)	B2	B7[1]	1.728(6)
C4B	B2[1]	1.737(5)	B3	B3[1]	1.748(8)
C4B	B3[1]	1.741(6)	B3	B4	1.753(6)
C4B	B4	1.739(6)	B4	B6	1.754(10)
C4B	B6	1.748(6)	B6	B7	1.741(7)
C4B	B7	1.711(6)	B6	B7[1]	1.741(7)
C5	C6	1.399(6)	B7	B7[1]	1.756(9)
C5	C10	1.382(5)			

[1]+X,3/2-Y,+Z

Table S28 Bond Angles for **7.**

Atom	Atom	Atom	Angle/˚	Atom	Atom	Atom	Angle/˚
C1	Si1	C1[1]	95.66(16)	C3	B1	B2[1]	59.5(2)
C1	Si1	C3	109.39(11)	C3	B1	B7[1]	104.8(3)
C1[1]	Si1	C3	109.39(11)	C3	B1	B7	104.8(3)
C5	Si1	C1	111.99(12)	B2	B1	B2[1]	108.5(4)
C5	Si1	C1[1]	111.99(12)	B2	B1	B7	107.3(3)
C5	Si1	C3	116.46(17)	B2	B1	B7[1]	59.1(2)
C11	Si2	C1	110.77(14)	B2[1]	B1	B7	59.1(2)
C12	Si2	C1	107.61(14)	B2[1]	B1	B7[1]	107.3(3)
C12	Si2	C11	106.54(17)	B7[1]	B1	B7	59.9(3)
C13	Si2	C1	113.51(15)	C3	B2	C4B[1]	105.0(3)
C13	Si2	C11	108.72(19)	C3	B2	B1	59.2(2)
C13	Si2	C12	109.45(18)	C3	B2	B3	58.7(2)
Si1	C1	Si2	128.80(14)	C3	B2	B7[1]	105.9(3)
C2	C1	Si1	104.40(19)	C4B[1]	B2	B1	107.8(3)
C2	C1	Si2	125.87(19)	C4B[1]	B2	B3	59.6(2)
C1	C2	C2[1]	117.59(15)	B1	B2	B3	107.8(3)
C1	C2	C14	125.1(2)	B7[1]	B2	C4B[1]	59.2(2)
C14	C2	C2[1]	117.21(14)	B7[1]	B2	B1	60.8(3)
B1	C3	Si1	117.2(3)	B7[1]	B2	B3	107.2(3)
B1	C3	B2[1]	61.34(19)	C3	B3	C4B[1]	105.1(3)
B1	C3	B2	61.34(19)	C3	B3	B2	59.11(19)

B2	C3	Si1	118.73(18)	C3	B3	B3^1	59.23(14)
B2^1	C3	Si1	118.73(18)	C3	B3	B4	105.8(3)
B2^1	C3	B2	111.6(4)	C4B^1	B3	B2	59.4(2)
B3^1	C3	Si1	120.7(2)	C4B^1	B3	B3^1	107.54(19)
B3	C3	Si1	120.7(2)	C4B^1	B3	B4	59.7(3)
B3^1	C3	B1	112.3(3)	B3^1	B3	B2	107.94(18)
B3	C3	B1	112.4(3)	B3^1	B3	B4	60.10(15)
B3	C3	B2^1	112.3(3)	B4	B3	B2	107.5(3)
B3^1	C3	B2	112.3(3)	C4B	B4	C4B^1	107.0(4)
B3	C3	B2	62.2(2)	C4B^1	B4	B3	59.8(2)
B3^1	C3	B2^1	62.2(2)	C4B	B4	B3^1	59.8(2)
B3^1	C3	B3	61.5(3)	C4B^1	B4	B3^1	107.4(3)
B2^1	C4B	B3^1	61.1(2)	C4B	B4	B3	107.4(3)
B2^1	C4B	B4	109.5(3)	C4B^1	B4	B6	60.1(3)
B2^1	C4B	B6	109.2(3)	C4B	B4	B6	60.1(3)
B3^1	C4B	B6	109.3(3)	B3	B4	B3^1	59.8(3)
B4	C4B	B3^1	60.5(3)	B3	B4	B6	108.5(4)
B4	C4B	B6	60.4(3)	B3^1	B4	B6	108.5(4)
B7	C4B	B2^1	60.1(2)	C4B^1	B6	C4B	106.2(4)
B7	C4B	B3^1	109.1(3)	C4B	B6	B4	59.6(3)
B7	C4B	B4	108.8(3)	C4B^1	B6	B4	59.6(3)
B7	C4B	B6	60.4(3)	B7^1	B6	C4B^1	58.7(2)
C6	C5	Si1	113.9(3)	B7	B6	C4B	58.7(2)
C10	C5	Si1	130.0(3)	B7	B6	C4B^1	106.7(3)
C10	C5	C6	116.1(4)	B7^1	B6	C4B	106.7(3)
C7	C6	C5	122.1(5)	B7^1	B6	B4	106.7(4)
C6	C7	C8	120.6(5)	B7	B6	B4	106.7(4)
C9	C8	C7	119.0(5)	B7^1	B6	B7	60.6(4)
C8	C9	C10	121.0(5)	C4B	B7	B1	108.5(3)
C5	C10	C9	121.2(4)	C4B	B7	B2^1	60.7(2)
C15	C14	C2	121.0(3)	C4B	B7	B6	60.8(3)
C19	C14	C2	120.7(3)	C4B	B7	B7^1	107.7(2)
C19	C14	C15	118.3(3)	B2^1	B7	B1	60.1(2)
C16	C15	C14	120.9(4)	B2^1	B7	B6	110.0(4)
C17	C16	C15	120.2(4)	B2^1	B7	B7^1	108.21(19)
C16	C17	C18	120.8(3)	B6	B7	B1	108.9(3)
C17	C18	C19	119.7(4)	B6	B7	B7^1	59.72(18)
C14	C19	C18	120.0(3)	B7^1	B7	B1	60.04(17)
C3	B1	B2	59.5(2)				

^{1}+X,3/2-Y,+Z

Calculated structures

Atomic coordinates of **HPS** at M06-2X/def2-TZVP

Energy: –1831.733175

C	2.793770	2.371997	1.165025
C	2.525719	1.497166	0.113605
C	3.471028	1.355129	-0.901569
C	4.650754	2.083284	-0.872790
C	4.909469	2.951215	0.179882
C	3.978898	3.090828	1.200781
C	1.242964	0.751275	0.074918
C	1.242948	-0.751303	-0.074909
C	2.525686	-1.497222	-0.113597
C	3.470995	-1.355212	0.901581
C	4.650704	-2.083394	0.872802
C	4.909403	-2.951325	-0.179874
C	3.978833	-3.090911	-1.200777
C	2.793722	-2.372053	-1.165022
C	0.030997	1.342267	0.159474
C	-0.247202	2.788194	0.207341
C	-1.233352	3.274466	1.069362
C	-1.538715	4.627148	1.112893
C	-0.877315	5.518496	0.280570
C	0.092396	5.046232	-0.595215
C	0.406064	3.697335	-0.630971
Si	-1.266679	0.000015	0.000000
C	-2.343813	-0.292129	1.501656
C	-1.925541	-1.152875	2.519813
C	-2.697381	-1.340650	3.658423
C	-3.903332	-0.668074	3.797797

C	-4.337353	0.189905	2.795648
C	-3.564554	0.372368	1.657732
C	0.030967	-1.342268	-0.159467
C	-0.247267	-2.788188	-0.207335
C	0.405976	-3.697345	0.630977
C	0.092274	-5.046234	0.595221
C	-0.877449	-5.518475	-0.280564
C	-1.538827	-4.627110	-1.112886
C	-1.233430	-3.274436	-1.069356
C	-2.343795	0.292182	-1.501664
C	-3.564561	-0.372268	-1.657739
C	-4.337345	-0.189789	-2.795663
C	-3.903281	0.668157	-3.797821
C	-2.697304	1.340686	-3.658449
C	-1.925480	1.152896	-2.519831
H	-0.989028	-1.690350	2.418705
H	-2.359739	-2.015390	4.434641
H	-4.507093	-0.815193	4.684284
H	-5.279370	0.713230	2.899525
H	-3.916537	1.040436	0.878456
H	-3.916576	-1.040310	-0.878457
H	-5.279382	-0.713078	-2.899540
H	-4.507030	0.815288	-4.684315
H	-2.359629	2.015401	-4.434675
H	-0.988946	1.690334	-2.418724
H	1.163425	-3.337303	1.316280
H	0.606743	-5.732896	1.255327
H	-1.119204	-6.572961	-0.308495
H	-2.298689	-4.983521	-1.796557

H	-1.751820	-2.582338	-1.723059
H	-1.751759	2.582382	1.723066
H	-2.298568	4.983578	1.796564
H	-1.119043	6.572989	0.308502
H	0.606883	5.732881	-1.255320
H	1.163505	3.337273	-1.316273
H	3.278483	-0.669778	1.718121
H	5.372217	-1.967976	1.671270
H	5.833488	-3.514084	-0.205627
H	4.174408	-3.763724	-2.025751
H	2.060132	-2.488084	-1.953482
H	3.278504	0.669695	-1.718106
H	5.372267	1.967845	-1.671254
H	5.833566	3.513953	0.205636
H	4.174485	3.763642	2.025751
H	2.060181	2.488049	1.953482

Atomic coordinates of **HPS** at M06-2X/6-31G*

Energy: –1831.150592

C	1.933951	1.207273	2.502632
C	2.345859	0.317189	1.500529
C	3.569829	-0.349457	1.667776
C	4.349884	-0.143191	2.802256
C	3.920567	0.741822	3.788987
C	2.712940	1.418372	3.637566
Si	1.264718	0.000012	0.000000
C	-0.038566	-1.343372	0.172494
C	0.245330	-2.791462	0.222085
C	-0.433391	-3.713704	-0.589987
C	-0.106551	-5.063963	-0.557077

C	0.901990	-5.524488	0.287911
C	1.588470	-4.620322	1.093197
C	1.269155	-3.266340	1.053613
C	-0.038592	1.343369	-0.172503
C	0.245272	2.791465	-0.222092
C	1.269090	3.266366	-1.053617
C	1.588374	4.620355	-1.093200
C	0.901868	5.524506	-0.287917
C	-0.106667	5.063958	0.557065
C	-0.433475	3.713691	0.589974
C	-1.257084	0.751420	-0.080946
C	-1.257069	-0.751448	0.080934
C	-2.541689	-1.499309	0.136023
C	-3.503699	-1.363178	-0.871329
C	-4.686380	-2.094095	-0.821756
C	-4.930391	-2.959974	0.241823
C	-3.983127	-3.094676	1.254245
C	-2.796082	-2.372018	1.199773
C	-2.541717	1.499259	-0.136032
C	-2.796122	2.371974	-1.199774
C	-3.983180	3.094610	-1.254244
C	-4.930446	2.959878	-0.241827
C	-4.686423	2.093993	0.821743
C	-3.503728	1.363099	0.871314
C	2.345878	-0.317143	-1.500520
C	1.934005	-1.207247	-2.502619
C	2.713010	-1.418331	-3.637546
C	3.920616	-0.741743	-3.788963
C	4.349899	0.143290	-2.802236

C	3.569829	0.349541	-1.667763
H	0.997980	-1.749739	-2.390068
H	2.380061	-2.114754	-4.401186
H	4.529643	-0.907311	-4.672719
H	5.293839	0.668285	-2.914670
H	3.919482	1.035199	-0.897948
H	3.919510	-1.035098	0.897959
H	5.293840	-0.668156	2.914694
H	4.529582	0.907402	4.672749
H	2.379964	2.114779	4.401210
H	0.997909	1.749736	2.390079
H	-1.219656	-3.361044	-1.251465
H	-0.640904	-5.760694	-1.195988
H	1.154044	-6.580163	0.312873
H	2.378261	-4.967590	1.752539
H	1.806518	-2.563126	1.685629
H	1.806473	2.563164	-1.685629
H	2.378160	4.967641	-1.752537
H	1.153899	6.580187	-0.312879
H	-0.641040	5.760677	1.195972
H	-1.219736	3.361013	1.251447
H	-3.321794	-0.678515	-1.695252
H	-5.421199	-1.982370	-1.613126
H	-5.856420	-3.525228	0.282836
H	-4.167501	-3.765754	2.087570
H	-2.049233	-2.482312	1.981053
H	-3.321814	0.678430	1.695231
H	-5.421244	1.982246	1.613109
H	-5.856485	3.525116	-0.282838

H	-4.167563	3.765694	-2.087562
H	-2.049271	2.482291	-1.981049

Atomic coordinates of the first excited state (S1) of **HPS** at M06-2X/6-31G*

Energy: –1831.131433

C	1.956788	1.295720	2.459177
C	2.380417	0.397573	1.468546
C	3.621790	-0.233650	1.644780
C	4.407452	0.016842	2.767110
C	3.967897	0.915130	3.736367
C	2.741518	1.556863	3.579123
Si	1.256675	-0.000067	-0.000002
C	-0.065407	-1.307845	0.187977
C	0.209622	-2.726619	0.190640
C	-0.651195	-3.704800	-0.371892
C	-0.283463	-5.041408	-0.408571
C	0.936590	-5.460411	0.122327
C	1.802669	-4.517372	0.678742
C	1.452271	-3.177985	0.701445
C	-0.065252	1.307875	-0.187948
C	0.209968	2.726613	-0.190609
C	1.452638	3.177811	-0.701512
C	1.803235	4.517146	-0.678812
C	0.937345	5.460299	-0.122296
C	-0.282717	5.041462	0.408709
C	-0.650649	3.704909	0.372033
C	-1.363882	0.700980	-0.121111
C	-1.363962	-0.700795	0.121158
C	-2.605043	-1.473397	0.325073
C	-3.663062	-1.433515	-0.596067

C	-4.803717	-2.201574	-0.395038
C	-4.915771	-3.014175	0.732779
C	-3.873218	-3.060145	1.655494
C	-2.724132	-2.305240	1.449238
C	-2.604879	1.473710	-0.325048
C	-2.723906	2.305487	-1.449269
C	-3.872913	3.060504	-1.655550
C	-4.915449	3.014714	-0.732807
C	-4.803458	2.202176	0.395062
C	-3.662885	1.434003	0.596116
C	2.380338	-0.397845	-1.468572
C	1.956580	-1.295943	-2.459193
C	2.741256	-1.557184	-3.579154
C	3.967710	-0.915601	-3.736423
C	4.407394	-0.017365	-2.767177
C	3.621785	0.233226	-1.644832
H	1.000449	-1.803196	-2.348084
H	2.396323	-2.262147	-4.329831
H	4.579582	-1.116177	-4.610977
H	5.364140	0.483648	-2.882707
H	3.985384	0.926549	-0.888039
H	3.985289	-0.927016	0.887979
H	5.364139	-0.484288	2.882621
H	4.579810	1.115629	4.610909
H	2.396686	2.261866	4.329810
H	1.000717	1.803090	2.348089
H	-1.590744	-3.400880	-0.818532
H	-0.954805	-5.765088	-0.861169
H	1.213311	-6.509805	0.096430

H	2.755263	-4.830860	1.094754
H	2.123759	-2.455249	1.158138
H	2.123980	2.454988	-1.158283
H	2.755836	4.830504	-1.094904
H	1.214224	6.509652	-0.096399
H	-0.953907	5.765229	0.861394
H	-1.590194	3.401119	0.818769
H	-3.579508	-0.802003	-1.475541
H	-5.608994	-2.166500	-1.122443
H	-5.809444	-3.610540	0.888392
H	-3.951239	-3.691059	2.535597
H	-1.903745	-2.350335	2.159937
H	-3.579382	0.802542	1.475631
H	-5.608722	2.167240	1.122489
H	-5.809059	3.611167	-0.888440
H	-3.950885	3.691366	-2.535695
H	-1.903532	2.350440	-2.159992

Atomic coordinates of **TPPS** at M06-2X/def2-TZVP

Energy: –2186.954773

C	-1.836923	-0.893407	2.633479
C	-2.381834	-0.220284	1.535000
C	-3.673734	0.296844	1.661643
C	-4.397177	0.142476	2.836461
C	-3.838817	-0.533202	3.912824
C	-2.554208	-1.049343	3.811451
Si	-1.323863	0.000018	0.000004
C	-2.381805	0.220324	-1.535010
C	-3.673740	-0.296720	-1.661640
C	-4.397158	-0.142364	-2.836475

C	-3.838737	0.533215	-3.912868
C	-2.554092	1.049270	-3.811509
C	-1.836833	0.893348	-2.633519
C	-0.049043	1.371374	0.118564
Si	-0.373042	3.197763	0.385483
C	-0.352238	4.156360	-1.229878
C	1.150018	0.755520	0.054834
C	1.150004	-0.755530	-0.054823
C	2.449237	-1.475713	-0.100359
C	3.310438	-1.326853	-1.185736
C	4.488546	-2.053747	-1.252495
C	4.832683	-2.923024	-0.224365
C	3.995074	-3.055634	0.873652
C	2.809701	-2.335119	0.933472
C	2.449263	1.475678	0.100379
C	3.310460	1.326799	1.185757
C	4.488580	2.053671	1.252522
C	4.832736	2.922946	0.224395
C	3.995132	3.055575	-0.873624
C	2.809746	2.335082	-0.933449
C	0.850231	3.954731	1.587416
C	-2.103487	3.349309	1.090535
C	-0.049068	-1.371361	-0.118552
Si	-0.373104	-3.197742	-0.385482
C	0.850194	-3.954747	-1.587369
C	-0.352393	-4.156327	1.229886
C	-2.103526	-3.349243	-1.090599
H	-2.206719	-2.816101	-2.037424
H	-1.048835	-3.725737	1.952163

H	0.518521	4.959108	1.861693
H	-0.665320	5.186281	-1.041061
H	0.640204	-4.188234	1.679788
H	-0.665250	-5.186307	1.041023
H	-1.048474	3.725649	-1.952281
H	-2.206732	2.816152	2.037346
H	2.152913	-2.431350	1.789611
H	4.262473	-3.721840	1.683902
H	5.753630	-3.488823	-0.277224
H	5.141047	-1.942568	-2.108819
H	3.043939	-0.647897	-1.986886
H	2.152963	2.431325	-1.789591
H	4.262546	3.721776	-1.683872
H	5.753693	3.488727	0.277257
H	5.141077	1.942478	2.108846
H	3.043947	0.647846	1.986905
H	0.640433	4.188454	-1.679603
H	-2.354256	4.398812	1.259518
H	-2.840056	2.940311	0.393359
H	0.912947	3.359846	2.500798
H	1.853755	4.030115	1.165759
H	0.518422	-4.959083	-1.861720
H	0.913022	-3.359820	-2.500716
H	1.853683	-4.030233	-1.165648
H	-2.354327	-4.398741	-1.259569
H	-2.840107	-2.940204	-0.393459
H	-4.121905	-0.838232	-0.836593
H	-5.396589	-0.551504	-2.912472
H	-4.402688	0.655596	-4.828805

H	-2.112077	1.573979	-4.649073
H	-0.832781	1.299659	-2.565162
H	-4.121852	0.838432	0.836620
H	-5.396579	0.551685	2.912468
H	-4.402787	-0.655593	4.828747
H	-2.112241	-1.574132	4.648991
H	-0.832901	-1.299790	2.565112

Atomic coordinates of **TPSS** at M06-2X/6-31G*

Energy: –2186.377654

C	2.800176	-2.381377	0.891085
C	2.460663	-1.481505	-0.122677
C	3.341416	-1.304299	-1.194578
C	4.516329	-2.043912	-1.269739
C	4.837666	-2.954632	-0.264195
C	3.981671	-3.115752	0.821840
C	1.162219	-0.754734	-0.070253
C	1.162226	0.754720	0.070255
C	-0.043048	1.370265	0.150161
Si	-0.365433	3.194854	0.466652
C	-2.111021	3.325720	1.165072
C	-0.043061	-1.370268	-0.150161
Si	-0.365470	-3.194850	-0.466666
C	-2.111068	-3.325682	-1.165068
Si	-1.324547	0.000004	0.000004
C	-2.387348	-0.280376	1.527923
C	-3.681424	0.239863	1.673560
C	-4.416350	0.027462	2.837513
C	-3.868167	-0.711456	3.883090
C	-2.582133	-1.232288	3.762918

C	-1.852891	-1.017499	2.596238
C	-2.387357	0.280390	-1.527908
C	-1.852915	1.017536	-2.596214
C	-2.582166	1.232332	-3.762888
C	-3.868193	0.711483	-3.883061
C	-4.416360	-0.027460	-2.837493
C	-3.681425	-0.239867	-1.673546
C	-0.334393	-4.219601	1.119750
C	0.860212	-3.914270	-1.705322
C	2.460674	1.481483	0.122674
C	3.341440	1.304258	1.194561
C	4.516357	2.043865	1.269718
C	4.837684	2.954600	0.264184
C	3.981674	3.115743	-0.821836
C	2.800175	2.381373	-0.891076
C	0.860274	3.914269	1.705285
C	-0.334358	4.219586	-1.119777
H	-2.228151	2.753597	2.091105
H	-1.017733	3.810914	-1.871613
H	0.517768	-4.901924	-2.034625
H	-0.661738	-5.241566	0.895886
H	0.666874	4.280167	-1.555995
H	-0.661642	5.241570	-0.895912
H	-1.017720	-3.810903	1.871616
H	-2.228202	-2.753542	-2.091090
H	2.128659	2.496703	-1.737905
H	4.232152	3.813164	-1.615538
H	5.756145	3.530200	0.324391
H	5.184375	1.909989	2.115019

H	3.091460	0.593196	1.977414
H	2.128673	-2.496688	1.737926
H	4.232158	-3.813159	1.615552
H	5.756124	-3.530236	-0.324404
H	5.184336	-1.910053	-2.115051
H	3.091428	-0.593248	-1.977439
H	0.666852	-4.280239	1.555931
H	-2.366483	-4.370359	-1.374226
H	-2.843580	-2.944784	-0.442517
H	0.942993	-3.274707	-2.590298
H	1.861383	-4.025003	-1.277288
H	0.517845	4.901929	2.034586
H	0.943060	3.274712	2.590264
H	1.861441	4.024989	1.277237
H	-2.366419	4.370404	1.374217
H	-2.843547	2.944820	0.442537
H	-4.122484	0.824709	0.869687
H	-5.417121	0.439109	2.928395
H	-4.441601	-0.879734	4.789784
H	-2.148414	-1.806310	4.576459
H	-0.847959	-1.427362	2.511772
H	-4.122472	-0.824732	-0.869680
H	-5.417125	-0.439120	-2.928376
H	-4.441634	0.879766	-4.789750
H	-2.148460	1.806373	-4.576421
H	-0.847989	1.427413	-2.511747

Atomic coordinates of the first excited state (S1) of **TPSS** at M06-2X/6-31G*

Energy: –2186.329929

C	2.921234	-1.973872	1.628263

C	2.493821	-1.368658	0.441018
C	3.262236	-1.522611	-0.717164
C	4.428402	-2.280927	-0.690767
C	4.844706	-2.885806	0.493710
C	4.091618	-2.726519	1.655709
C	1.228225	-0.595890	0.410149
C	1.165261	0.811958	0.248004
C	-0.172639	1.326749	0.308590
Si	-0.710541	2.894499	1.188191
C	-2.142371	2.595808	2.378418
C	-0.029873	-1.249388	0.468476
Si	-0.401716	-3.059327	0.812310
C	-2.111249	-3.470545	0.131564
Si	-1.267521	-0.050044	-0.276530
C	-3.005378	-0.029032	0.416091
C	-4.057457	0.587954	-0.275644
C	-5.314309	0.732438	0.309354
C	-5.542641	0.245191	1.593875
C	-4.512809	-0.381175	2.294616
C	-3.256047	-0.505263	1.711666
C	-1.336586	-0.210339	-2.154290
C	-0.303425	0.309803	-2.947026
C	-0.300846	0.134566	-4.328082
C	-1.338205	-0.563055	-4.943514
C	-2.373668	-1.086974	-4.174011
C	-2.369516	-0.912525	-2.791550
C	-0.359686	-3.501548	2.647007
C	0.829567	-4.145251	-0.125495
C	2.315085	1.695728	-0.013466

C	3.551996	1.545063	0.633728
C	4.585872	2.443756	0.397954
C	4.416327	3.503299	-0.491661
C	3.196121	3.661251	-1.145585
C	2.156280	2.771547	-0.903623
C	0.730333	3.608613	2.172440
C	-1.306109	4.147976	-0.095108
H	-1.924455	1.786410	3.081895
H	-2.099326	3.716225	-0.715093
H	0.467011	-5.180185	-0.114696
H	-0.675154	-4.542239	2.784145
H	-0.495861	4.473133	-0.754621
H	-1.712385	5.035057	0.404975
H	-1.033341	-2.868442	3.233297
H	-2.169811	-3.223307	-0.934820
H	1.200603	2.895904	-1.407832
H	3.052333	4.480902	-1.843216
H	5.227749	4.201768	-0.670705
H	5.530048	2.319197	0.919511
H	3.693138	0.736833	1.343499
H	2.332202	-1.836989	2.531474
H	4.416035	-3.188703	2.583151
H	5.754807	-3.477271	0.512054
H	5.013409	-2.402242	-1.597300
H	2.929097	-1.051415	-1.638093
H	0.647218	-3.401253	3.062779
H	-2.290170	-4.547596	0.229432
H	-2.923586	-2.948667	0.645025
H	0.908689	-3.825527	-1.169488

H	1.831422	-4.124158	0.311966
H	0.373070	4.431188	2.802512
H	1.168797	2.848549	2.828000
H	1.526658	3.992937	1.528515
H	-2.310235	3.512330	2.956945
H	-3.073265	2.348676	1.858783
H	-3.891095	0.966360	-1.282326
H	-6.114810	1.222220	-0.236972
H	-6.521935	0.354392	2.050300
H	-4.688360	-0.759080	3.297327
H	-2.445013	-0.962956	2.277296
H	-3.186252	-1.324310	-2.201386
H	-3.184981	-1.630002	-4.649784
H	-1.339722	-0.696818	-6.021239
H	0.507538	0.545513	-4.925753
H	0.511211	0.857845	-2.477017

Atomic coordinates of **1** at M06-2X/def2-TZVP

Energy: –2031.503247

C	0.715953	2.918705	2.005510
C	-0.267313	2.610537	1.061762
C	-0.936371	3.672313	0.440605
C	-0.632260	4.986677	0.753768
C	0.343062	5.276151	1.700110
C	1.013269	4.236339	2.326681
C	-0.512076	1.213985	0.638443
C	-1.716250	0.679309	0.326698
C	-3.000831	1.378884	0.569348
C	-3.918102	1.570354	-0.463009
C	-5.097324	2.261925	-0.233103

C	-5.382421	2.751736	1.034809
C	-4.479594	2.553195	2.070826
C	-3.292690	1.875786	1.837868
C	-1.716405	-0.678896	-0.326866
C	-3.001135	-1.378217	-0.569445
C	-3.918430	-1.569418	0.462941
C	-5.097802	-2.260756	0.233104
C	-5.383022	-2.750601	-1.034768
C	-4.480168	-2.552329	-2.070813
C	-3.293118	-1.875153	-1.837924
C	-0.512353	-1.213888	-0.638531
Si	0.751102	-0.000077	-0.000070
C	1.761305	0.872941	-1.381729
B	0.830347	2.029143	-2.261401
B	1.535232	2.067125	-3.874303
B	1.971707	3.307914	-2.696292
C	2.111251	2.468943	-1.241124
B	3.546325	2.891156	-2.013067
B	3.388134	1.357141	-1.150166
B	4.098368	1.385773	-2.762457
B	3.223310	2.597088	-3.725164
B	2.853155	0.879095	-3.920666
B	2.948335	0.113943	-2.328607
B	1.377905	0.535692	-3.014400
C	-0.267933	-2.610561	-1.061664
C	-0.937569	-3.672040	-0.440626
C	-0.633825	-4.986545	-0.753558
C	0.341711	-5.276461	-1.699542
C	1.012501	-4.236948	-2.325987

C	0.715540	-2.919173	-2.005050
C	1.761152	-0.873290	1.381568
C	2.110553	-2.469413	1.241110
B	0.829912	-2.029069	2.261486
B	1.970903	-3.308169	2.696392
B	3.545578	-2.892011	2.012931
B	4.098206	-1.386725	2.762077
B	2.853294	-0.879499	3.920375
B	3.222863	-2.597634	3.725032
B	1.534979	-2.067096	3.874304
B	1.378052	-0.535714	3.014242
B	2.948543	-0.114569	2.328214
B	3.387799	-1.358053	1.149867
H	0.557738	-0.282447	-3.231436
H	0.845334	2.311097	-4.801042
H	4.215180	-3.722268	1.511710
H	1.827804	-2.938305	0.310322
H	5.247283	1.149153	-2.894763
H	0.558184	0.282734	3.231264
H	0.845118	-2.310741	4.801158
H	1.578837	-4.419021	2.651477
H	3.890991	-1.169423	0.106577
H	3.756266	-3.240775	4.559700
H	3.214940	1.008541	2.082201
H	3.114372	-0.264630	4.894837
H	5.247210	-1.150454	2.894234
H	4.216267	3.721128	-1.511830
H	3.214391	-1.009285	-2.082761
H	3.113912	0.264251	-4.895230

H	3.756822	3.240154	-4.559821
H	-0.276741	2.230102	-1.919248
H	1.580020	4.418894	-2.651194
H	1.828770	2.937821	-0.310250
H	-2.579648	1.727781	2.640044
H	-4.698201	2.929236	3.061723
H	-6.307303	3.283814	1.215206
H	-5.797653	2.413185	-1.044057
H	-3.703186	1.179549	-1.450371
H	-2.580056	-1.727359	-2.640121
H	-4.698870	-2.928398	-3.061679
H	-6.308019	-3.282497	-1.215111
H	-5.798150	-2.411808	1.044079
H	-3.703416	-1.178584	1.450271
H	-0.277280	-2.229699	1.919487
H	3.891401	1.168227	-0.106962
H	1.258139	-2.123731	-2.494565
H	1.776635	-4.446641	-3.063124
H	0.579924	-6.304104	-1.940240
H	-1.156845	-5.788762	-0.249434
H	-1.689798	-3.462378	0.308084
H	1.258093	2.123015	2.495138
H	1.777225	4.445689	3.064100
H	0.581560	6.303686	1.940989
H	-1.154822	5.789132	0.249545
H	-1.688411	3.462991	-0.308387

Atomic coordinates of **1** at M06-2X/6-31G*

Energy: –2030.894008

C	0.738391	2.906575	2.004776

C	-0.266808	2.607400	1.073821
C	-0.937479	3.678643	0.459298
C	-0.610152	4.994101	0.764703
C	0.389292	5.274853	1.695250
C	1.058969	4.225744	2.317332
C	-0.521975	1.210087	0.649139
Si	0.747608	-0.000301	-0.000191
C	1.761286	0.887190	-1.380118
B	3.389876	1.373832	-1.144386
B	2.954315	0.137157	-2.334918
B	1.380315	0.560305	-3.019345
B	1.533809	2.101595	-3.868846
B	3.223397	2.636298	-3.713419
B	2.857816	0.915616	-3.923569
B	4.103167	1.417451	-2.758030
B	3.545216	2.917900	-1.995967
B	1.966617	3.336625	-2.678250
B	0.827088	2.048854	-2.254434
C	2.107453	2.485170	-1.225875
C	-1.733463	0.678226	0.332374
C	-3.017551	1.379458	0.589226
C	-3.953049	1.569144	-0.434287
C	-5.132682	2.263065	-0.186969
C	-5.398973	2.758537	1.087690
C	-4.477893	2.563084	2.114343
C	-3.291375	1.881841	1.865703
C	-1.733984	-0.676962	-0.332621
C	-3.018606	-1.377256	-0.589375
C	-3.954288	-1.566072	0.434134

C	-5.134455	-2.259104	0.186875
C	-5.401091	-2.754549	-1.087722
C	-4.479819	-2.559976	-2.114368
C	-3.292777	-1.879624	-1.865784
C	-0.522913	-1.209792	-0.649372
C	-0.268907	-2.607425	-1.073704
C	0.735926	-2.907736	-2.004674
C	1.055341	-4.227270	-2.316876
C	0.384840	-5.275623	-1.694409
C	-0.614253	-4.993741	-0.763826
C	-0.940416	-3.677911	-0.458778
C	1.760701	-0.888415	1.379771
B	1.380219	-0.560644	3.018941
B	2.954589	-0.139306	2.334245
B	2.857443	-0.917194	3.923137
B	3.221256	-2.638306	3.713457
B	4.102177	-1.420634	2.757626
B	3.388781	-1.376758	1.144025
B	3.542644	-2.920737	1.996058
C	2.105260	-2.486778	1.225968
B	1.963677	-3.337659	2.678605
B	1.532223	-2.101840	3.868875
B	0.825420	-2.048861	2.254507
H	0.560699	0.263957	3.241957
H	0.840099	-2.351339	4.798391
H	4.214361	3.749196	-1.485227
H	1.821816	2.947876	-0.287468
H	5.256582	-1.188631	2.891864
H	0.559902	-0.263339	-3.242552

H	0.841857	2.352068	-4.798227
H	1.570235	4.450396	-2.623344
H	3.892007	1.176803	-0.096369
H	3.757969	3.289709	-4.545948
H	3.222913	-0.992060	-2.095781
H	3.122191	0.306836	-4.906459
H	5.257324	1.184317	-2.892445
H	4.210901	-3.752860	1.485503
H	3.224309	0.989570	2.094774
H	3.122528	-0.308392	4.905823
H	3.755241	-3.292017	4.546127
H	-0.287279	-2.242041	1.909231
H	1.566163	-4.451043	2.624062
H	1.819092	-2.949471	0.287721
H	-2.565542	-1.732527	-2.659687
H	-4.684272	-2.940538	-3.110138
H	-6.326334	-3.288363	-1.280878
H	-5.849444	-2.407274	0.989950
H	-3.752542	-1.170308	1.425826
H	-2.564278	1.734064	2.659607
H	-4.682085	2.943661	3.110162
H	-6.323799	3.293056	1.280891
H	-5.847528	2.411911	-0.990047
H	-3.751576	1.173351	-1.426024
H	-0.285391	2.243064	-1.909034
H	3.890991	-1.180541	0.095885
H	1.277776	2.100807	2.489289
H	1.840152	4.428228	3.043283
H	0.646180	6.303272	1.928195

H	-1.132910	5.804026	0.265724
H	-1.707532	3.473648	-0.277523
H	1.275962	-2.102556	-2.489432
H	1.836272	-4.430638	-3.042852
H	0.640821	-6.304330	-1.927083
H	-1.137650	-5.803067	-0.264545
H	-1.710208	-3.472035	0.278069

Atomic coordinates of the first excited state (S1) of **1** at M06-2X/6-31G*

Energy: –2030.874538

C	0.839324	3.017910	1.566252
C	-0.308994	2.625258	0.837593
C	-1.150672	3.657834	0.351768
C	-0.849522	4.992126	0.581346
C	0.279535	5.350745	1.317537
C	1.122110	4.353303	1.811049
C	-0.534824	1.227559	0.522855
Si	0.751707	-0.000007	-0.000017
C	1.823988	0.757327	-1.443834
B	3.450732	1.280344	-1.272622
B	3.023661	-0.077667	-2.319390
B	1.443475	0.251428	-3.037545
B	1.580768	1.691124	-4.054649
B	3.266479	2.253369	-3.965804
B	2.914011	0.517640	-3.983580
B	4.159782	1.155557	-2.885578
B	3.592146	2.723564	-2.290839
B	2.007513	3.051506	-3.008516
B	0.880209	1.809710	-2.439776
C	2.159466	2.364054	-1.472768

C	-1.830358	0.648328	0.289459
C	-3.068718	1.360281	0.657558
C	-4.121264	1.528340	-0.255744
C	-5.258960	2.238751	0.105720
C	-5.373106	2.780425	1.386126
C	-4.336323	2.616451	2.301547
C	-3.187777	1.922411	1.937811
C	-1.830369	-0.648279	-0.289534
C	-3.068743	-1.360208	-0.657636
C	-4.121296	-1.528243	0.255663
C	-5.259006	-2.238629	-0.105804
C	-5.373163	-2.780299	-1.386211
C	-4.336376	-2.616342	-2.301632
C	-3.187815	-1.922328	-1.937893
C	-0.534847	-1.227545	-0.522907
C	-0.309047	-2.625260	-0.837606
C	0.839246	-3.017961	-1.566276
C	1.122014	-4.353369	-1.811008
C	0.279448	-5.350778	-1.317416
C	-0.849584	-4.992110	-0.581211
C	-1.150718	-3.657803	-0.351699
C	1.823917	-0.757359	1.443842
B	1.443373	-0.251444	3.037541
B	3.023588	0.077600	2.319429
B	2.913875	-0.517701	3.983617
B	3.266290	-2.253441	3.965854
B	4.159657	-1.155658	2.885649
B	3.450647	-1.280428	1.272675
B	3.591988	-2.723649	2.290899

C	2.159341	-2.364097	1.472788
B	2.007325	-3.051541	3.008534
B	1.580595	-1.691143	4.054651
B	0.880076	-1.809710	2.439759
H	0.633936	0.605677	3.161425
H	0.884422	-1.833741	5.003727
H	4.254489	3.612965	-1.877582
H	1.872805	2.926431	-0.592103
H	5.315259	-0.917999	2.999976
H	0.634014	-0.605669	-3.161451
H	0.884627	1.833746	-5.003744
H	1.603397	4.161995	-3.075040
H	3.960471	1.199250	-0.212466
H	3.793331	2.815977	-4.867001
H	3.316147	-1.170049	-1.966933
H	3.180914	-0.194105	-4.894240
H	5.315379	0.917862	-2.999873
H	4.254315	-3.613072	1.877662
H	3.316113	1.169975	1.966981
H	3.180776	0.194038	4.894282
H	3.793101	-2.816062	4.867066
H	-0.231602	-2.039455	2.108752
H	1.603173	-4.162017	3.075049
H	1.872688	-2.926467	0.592116
H	-2.372504	-1.801852	-2.646095
H	-4.418149	-3.034889	-3.299754
H	-6.265855	-3.330499	-1.666623
H	-6.060086	-2.371216	0.614611
H	-4.034653	-1.109012	1.253633

H	-2.372467	1.801921	2.646013
H	-4.418087	3.035004	3.299667
H	-6.265786	3.330646	1.666535
H	-6.060036	2.371354	-0.614697
H	-4.034628	1.109107	-1.253714
H	-0.231470	2.039489	-2.108797
H	3.960416	-1.199353	0.212531
H	1.500753	2.254888	1.961408
H	2.003694	4.616713	2.387007
H	0.504964	6.396623	1.499494
H	-1.499029	5.760812	0.174558
H	-2.015559	3.404411	-0.250398
H	1.500668	-2.254967	-1.961494
H	2.003579	-4.616818	-2.386979
H	0.504865	-6.396669	-1.499319
H	-1.499085	-5.760769	-0.174361
H	-2.015585	-3.404342	0.250481

Atomic coordinates of **2** at M06-2X/def2-TZVP

Energy: –2031.551997

C	-0.932379	3.677807	0.258261
C	-0.304838	2.652878	0.974566
C	0.579414	3.013184	1.992144
C	0.823813	4.346521	2.289434
C	0.201155	5.350740	1.563879
C	-0.678602	5.008666	0.544741
C	-0.519626	1.238387	0.590355
Si	0.753243	-0.000017	-0.000023
C	1.779829	0.826779	-1.391335
B	0.910936	1.972508	-2.340003

B	2.198943	2.475393	-1.257175
B	3.426726	1.203415	-1.242329
B	4.107025	1.065008	-2.858322
B	3.297841	2.259960	-3.883214
C	3.567422	2.548071	-2.237186
B	2.119434	3.133224	-2.888409
B	1.586574	1.845172	-3.972494
B	2.815765	0.561748	-3.953771
B	2.892918	-0.090657	-2.305715
B	1.342958	0.399927	-2.999289
C	-1.722042	0.693112	0.296348
C	-1.722073	-0.693023	-0.296428
C	-3.008603	-1.403570	-0.499171
C	-3.919755	-1.544317	0.546538
C	-5.104727	-2.238254	0.356737
C	-5.402196	-2.783154	-0.885520
C	-4.505128	-2.637238	-1.935056
C	-3.312946	-1.956555	-1.741178
C	-3.008540	1.403714	0.499098
C	-3.312863	1.956692	1.741114
C	-4.505015	2.637424	1.935000
C	-5.402075	2.783396	0.885464
C	-5.104627	2.238502	-0.356801
C	-3.919685	1.544517	-0.546610
C	-0.519681	-1.238364	-0.590406
C	-0.304955	-2.652882	-0.974556
C	0.579378	-3.013286	-1.992030
C	0.823715	-4.346651	-2.289249
C	0.200912	-5.350801	-1.563724

C	-0.678926	-5.008631	-0.544689
C	-0.932639	-3.677744	-0.258281
C	1.779746	-0.826867	1.391318
B	1.342913	-0.399923	2.999256
B	2.892907	0.090522	2.305659
B	2.815715	-0.561817	3.953738
B	3.297669	-2.260065	3.883237
B	4.106935	-1.065208	2.858302
B	3.426617	-1.203625	1.242318
C	3.567222	-2.548255	2.237219
B	2.198741	-2.475513	1.257209
B	2.119194	-3.133280	2.888469
B	1.586432	-1.845151	3.972512
B	0.910777	-1.972497	2.340027
H	0.479367	0.382382	3.170336
H	0.921144	-2.072895	4.920643
H	0.921295	2.072993	-4.920613
H	0.479349	-0.382303	-3.170396
H	5.262287	-0.857349	2.962285
H	4.052956	1.093913	-0.256746
H	5.262361	0.857070	-2.962322
H	3.036010	-0.124378	-4.889116
H	-0.209286	2.219420	-2.073219
H	3.928110	2.852752	-4.684101
H	2.086205	3.116570	-0.279285
H	4.317858	3.271143	-1.956532
H	1.957178	4.293509	-3.014471
H	4.317603	-3.271392	1.956587
H	3.109595	1.210758	2.001220

H	3.036010	0.124327	4.889057
H	3.927893	-2.852877	4.684145
H	-0.209464	-2.219342	2.073262
H	1.956857	-4.293549	3.014579
H	2.085958	-3.116721	0.279345
H	-2.604462	-1.850262	-2.553859
H	-4.732055	-3.057363	-2.906256
H	-6.331377	-3.317369	-1.035264
H	-5.800029	-2.348142	1.178702
H	-3.695706	-1.109971	1.513493
H	-2.604386	1.850355	2.553795
H	-4.731927	3.057543	2.906205
H	-6.331232	3.317649	1.035214
H	-5.799921	2.348434	-1.178766
H	-3.695652	1.110176	-1.513571
H	3.109529	-1.210920	-2.001324
H	4.052847	-1.094199	0.256728
H	1.088433	-2.248148	-2.558308
H	1.511290	-4.597446	-3.086771
H	0.399340	-6.390847	-1.787562
H	-1.168742	-5.781711	0.033204
H	-1.615589	-3.424603	0.541777
H	1.088351	2.247987	2.558451
H	1.511322	4.597241	3.087037
H	0.399632	6.390764	1.787773
H	-1.168305	5.781800	-0.033175
H	-1.615265	3.424741	-0.541875

Atomic coordinates of **2** at M06-2X/6-31G*

Energy: -2030.941562

C	-0.938556	3.686108	0.245798
C	-0.305649	2.659619	0.965056
C	0.593458	3.022326	1.975498
C	0.849733	4.360657	2.261446
C	0.223308	5.366149	1.532017
C	-0.672988	5.021587	0.521878
C	-0.526502	1.240875	0.588966
C	-1.736119	0.694271	0.296301
C	-3.022831	1.406823	0.512037
C	-3.956858	1.537509	-0.521858
C	-5.142953	2.233460	-0.314412
C	-5.417725	2.792108	0.931931
C	-4.497660	2.657776	1.969156
C	-3.305280	1.973585	1.759253
C	-1.736086	-0.694329	-0.296294
C	-3.022764	-1.406937	-0.512044
C	-3.305167	-1.973726	-1.759258
C	-4.497514	-2.657969	-1.969175
C	-5.417592	-2.792328	-0.931964
C	-5.142865	-2.233654	0.314377
C	-3.956804	-1.537650	0.521836
C	-0.526444	-1.240878	-0.588954
Si	0.752781	0.000021	0.000015
C	1.782393	0.824873	-1.399966
B	0.912103	1.968837	-2.355688
B	1.588480	1.834185	-3.990640
B	2.122136	3.129090	-2.909806
B	2.202257	2.477288	-1.273362
C	3.574332	2.546530	-2.254307

B	3.432092	1.202368	-1.253472
B	4.113975	1.057126	-2.871520
B	3.302715	2.249971	-3.902874
B	2.820466	0.548637	-3.966989
B	2.898323	-0.097724	-2.313648
B	1.345375	0.390577	-3.009566
C	-0.305521	-2.659603	-0.965063
C	-0.938399	-3.686136	-0.245842
C	-0.672770	-5.021596	-0.521952
C	0.223560	-5.366095	-1.532082
C	0.849959	-4.360559	-2.261472
C	0.593622	-3.022246	-1.975495
C	1.782407	-0.824808	1.399997
B	1.345329	-0.390581	3.009597
B	2.898264	0.097830	2.313725
B	2.820409	-0.548579	3.967049
B	1.588497	-1.834200	3.990641
B	2.122251	-3.129046	2.909785
C	3.574427	-2.546384	2.254331
B	3.302758	-2.249883	3.902899
B	4.113970	-1.056965	2.871594
B	3.432131	-1.202205	1.253528
B	2.202368	-2.477195	1.273359
B	0.912163	-1.968848	2.355671
H	0.478912	-0.397203	-3.176312
H	0.920850	2.058882	-4.943667
H	0.920859	-2.058958	4.943647
H	0.478814	0.397140	3.176346
H	5.274098	0.849750	-2.974966

H	4.059778	-1.095931	0.262464
H	5.274079	-0.849524	2.975071
H	3.041267	0.143798	4.903477
H	-0.213093	-2.215997	2.087792
H	3.936045	-2.842420	4.708290
H	2.091414	-3.123990	0.293059
H	4.328011	-3.273473	1.975626
H	1.961395	-4.293508	3.041556
H	4.327868	3.273670	-1.975604
H	3.114397	-1.221398	-2.002272
H	3.041381	-0.143751	-4.903395
H	3.935984	2.842525	-4.708267
H	-0.213173	2.215924	-2.087839
H	1.961215	4.293540	-3.041611
H	2.091252	3.124104	-0.293082
H	-2.578808	1.874629	2.561262
H	-4.707045	3.088705	2.943256
H	-6.347438	3.328484	1.094669
H	-5.856412	2.334324	-1.126288
H	-3.749617	1.091723	-1.490966
H	-2.578684	-1.874748	-2.561255
H	-4.706864	-3.088918	-2.943273
H	-6.347279	-3.328745	-1.094712
H	-5.856335	-2.334538	1.126242
H	-3.749599	-1.091844	1.490942
H	3.114279	1.221524	2.002382
H	4.059723	1.096154	-0.262393
H	1.101943	2.253474	2.544399
H	1.548515	4.613688	3.052969

H	0.431111	6.409828	1.746645
H	-1.166425	5.796059	-0.057212
H	-1.635397	3.428088	-0.545533
H	1.102090	-2.253357	-2.544364
H	1.548768	-4.613541	-3.052988
H	0.431410	-6.409760	-1.746733
H	-1.166186	-5.796104	0.057110
H	-1.635269	-3.428162	0.545478

Atomic coordinates of the first excited state (S1) of **2** at M06-2X/6-31G*

Energy: –2030.921121

C	-1.159919	3.651710	0.120666
C	-0.351655	2.664289	0.736326
C	0.694942	3.114978	1.570148
C	0.907845	4.466164	1.798326
C	0.104447	5.419503	1.173346
C	-0.921534	5.002095	0.325497
C	-0.543071	1.248922	0.464936
C	-1.839533	0.663545	0.251166
C	-3.080708	1.398949	0.561952
C	-4.123913	1.505029	-0.371231
C	-5.268572	2.231709	-0.069486
C	-5.399733	2.854450	1.171712
C	-4.372499	2.753864	2.106662
C	-3.217641	2.042254	1.801486
C	-1.839714	-0.663244	-0.250857
C	-3.081087	-1.398353	-0.561540
C	-3.218236	-2.041703	-1.801029
C	-4.373294	-2.753024	-2.106117
C	-5.400520	-2.853280	-1.171122

C	-5.269144	-2.230501	0.070034
C	-4.124281	-1.504108	0.371692
C	-0.543411	-1.248943	-0.464729
Si	0.752474	-0.000146	-0.000005
C	1.846306	0.713708	-1.446086
B	0.973389	1.760317	-2.507237
B	1.655407	1.483141	-4.123172
B	2.176853	2.873297	-3.163259
B	2.256439	2.370600	-1.474815
C	3.632170	2.365725	-2.454425
B	3.495916	1.115900	-1.333959
B	4.184223	0.832863	-2.932216
B	3.366594	1.918563	-4.070201
B	2.896946	0.215590	-3.979082
B	2.975966	-0.273645	-2.273347
B	1.420886	0.133467	-3.011968
C	-0.352424	-2.664357	-0.736120
C	-1.161090	-3.651525	-0.120580
C	-0.923226	-5.001982	-0.325537
C	0.102618	-5.419704	-1.173398
C	0.906416	-4.466618	-1.798251
C	0.694030	-3.115373	-1.569947
C	1.846527	-0.714102	1.445849
B	1.421346	-0.133903	3.011809
B	2.976309	0.273239	2.272957
B	2.897554	-0.216034	3.978695
B	1.656046	-1.483598	4.122946
B	2.177358	-2.873729	3.162920
C	3.632560	-2.366128	2.453875

B	3.367230	-1.919005	4.069702
B	4.184675	-0.833273	2.931618
B	3.496125	-1.116279	1.333460
B	2.256678	-2.370988	1.474476
B	0.973782	-1.760745	2.507109
H	0.565516	-0.680893	-3.105504
H	0.990030	1.615860	-5.095113
H	0.990819	-1.616344	5.094986
H	0.565978	0.680441	3.105493
H	5.346220	0.625450	-3.015274
H	4.126322	-1.101989	0.338064
H	5.346682	-0.625852	3.014501
H	3.126373	0.558206	4.846923
H	-0.152627	-2.030872	2.260218
H	3.998904	-2.440754	4.924114
H	2.138599	-3.110620	0.564196
H	4.378769	-3.122596	2.241276
H	2.008418	-4.020473	3.400010
H	4.378405	3.122205	-2.241959
H	3.211862	-1.361739	-1.866463
H	3.125640	-0.558669	-4.847327
H	3.998134	2.440297	-4.924722
H	-0.152987	2.030440	-2.260188
H	2.007867	4.020034	-3.400350
H	2.138494	3.110251	-0.564532
H	-2.410365	1.972005	2.525287
H	-4.466445	3.236237	3.074571
H	-6.297387	3.418038	1.406207
H	-6.061809	2.313534	-0.806099

H	-4.024844	1.021237	-1.338417
H	-2.410973	-1.971706	-2.524869
H	-4.467406	-3.235429	-3.073995
H	-6.298333	-3.416643	-1.405549
H	-6.062374	-2.312072	0.806682
H	-4.025049	-1.020282	1.338844
H	3.212133	1.361340	1.866049
H	4.126258	1.101638	-0.338653
H	1.330850	2.388025	2.059899
H	1.709293	4.776820	2.461493
H	0.278565	6.477709	1.341502
H	-1.541210	5.735345	-0.181219
H	-1.948784	3.346270	-0.557796
H	1.330237	-2.388619	-2.059607
H	1.707764	-4.777522	-2.461421
H	0.276322	-6.477961	-1.341662
H	-1.543205	-5.735042	0.181082
H	-1.949858	-3.345845	0.557887

Atomic coordinates of **4** at M06-2X/def2-TZVP

Energy: –2386.722669

C	3.280520	2.380646	-0.425930
C	2.873447	1.374375	0.443074
C	3.711703	0.995810	1.489538
C	4.921338	1.641995	1.684585
C	5.315126	2.660439	0.824223
C	4.497252	3.023010	-0.235632
C	1.554448	0.713496	0.254426
C	0.365244	1.314346	0.495747
Si	0.251508	3.110878	1.107130

C	-1.429966	3.532584	1.830962
C	1.554314	-0.713684	-0.254541
C	0.364983	-1.314334	-0.495770
Si	-0.865460	0.000102	0.000040
C	-1.897446	0.583633	-1.503120
B	-0.900785	1.365771	-2.666657
C	-1.579160	-0.162839	-2.923064
B	-3.088471	-0.428010	-2.195426
B	-4.169386	0.657128	-3.067213
B	-3.225305	1.477635	-4.331109
B	-2.909924	-0.219850	-3.946160
B	-1.561828	0.888503	-4.235928
B	-1.972791	2.459006	-3.535949
B	-3.591244	2.317425	-2.818713
B	-3.504196	1.140751	-1.500644
B	-2.158777	2.239549	-1.792285
C	2.873210	-1.374752	-0.443244
C	3.280220	-2.381020	0.425792
C	4.496861	-3.023540	0.235456
C	5.314721	-2.661119	-0.824462
C	4.921012	-1.642660	-1.684841
C	3.711465	-0.996321	-1.489758
Si	0.250772	-3.110836	-1.107158
C	1.480342	-3.399946	-2.496200
C	-1.430985	-3.532146	-1.830564
C	0.581096	-4.310001	0.306022
C	-1.897419	-0.583360	1.503242
B	-2.158909	-2.239265	1.792331
B	-3.504230	-1.140312	1.500819

B	-3.591330	-2.317048	2.818829
B	-3.225231	-1.477377	4.331253
B	-4.169290	-0.656704	3.067445
B	-3.088307	0.428370	2.195661
B	-2.909696	0.220097	3.946375
C	-1.578984	0.163003	2.923212
B	-1.561702	-0.888407	4.236023
B	-1.972857	-2.458831	3.535981
B	-0.900780	-1.365663	2.666691
C	1.481514	3.399717	2.495838
C	0.581769	4.309941	-0.306151
H	-1.824020	-3.004156	0.963089
H	-1.541463	-3.464682	3.980251
H	-1.541316	3.464793	-3.980287
H	0.412662	3.859394	-1.284883
H	1.207323	-4.322555	-3.014447
H	-1.823768	3.004456	-0.963110
H	-0.082845	-5.172787	0.222433
H	1.610964	4.669650	-0.267314
H	-0.081960	5.172877	-0.222424
H	0.412322	-3.859441	1.284804
H	-1.653948	-3.023418	-2.769729
H	-5.309310	-0.373793	3.183119
H	-3.301679	-1.466507	-1.691615
H	-5.309439	0.374326	-3.182814
H	-4.329477	3.236703	-2.750073
H	0.255204	1.431826	-2.439998
H	-3.698545	1.783995	-5.368796
H	-0.879064	-0.981065	-2.865793

H	-3.044845	-1.177771	-4.620778
H	-0.793059	0.671688	-5.103162
H	-3.044489	1.177996	4.621052
H	-4.086137	-1.159891	0.477802
H	-4.329661	-3.236247	2.750180
H	-3.698451	-1.783746	5.368946
H	0.255192	-1.431826	2.439974
H	-0.792873	-0.671714	5.103235
H	-0.878803	0.981159	2.865956
H	2.639619	2.659182	-1.253670
H	4.803926	3.806941	-0.916053
H	6.260544	3.164015	0.977415
H	5.558908	1.352479	2.509627
H	3.403475	0.202357	2.160151
H	2.639337	-2.659436	1.253587
H	4.803480	-3.807474	0.915899
H	6.260072	-3.164812	-0.977682
H	5.558570	-1.353252	-2.509930
H	3.403297	-0.202856	-2.160385
H	1.610200	-4.669942	0.266934
H	-1.398953	-4.602305	-2.054653
H	-2.263206	-3.370178	-1.147642
H	1.460513	-2.590171	-3.229557
H	2.505184	-3.501806	-2.138475
H	1.208869	4.322405	3.014143
H	1.461682	2.589961	3.229215
H	2.506286	3.501302	2.137828
H	-1.397640	4.602744	2.055005
H	-2.262405	3.370775	1.148271

H	-1.652791	3.023941	2.770205
H	-4.086046	1.160445	-0.477596
H	-3.301435	1.466912	1.691918

Atomic coordinates of **4** at M06-2X/6-31G*

Energy: –2386.117074

C	-3.730590	1.001497	-1.485134
C	-2.883876	1.379689	-0.438764
C	-3.284667	2.392733	0.434138
C	-4.500857	3.045258	0.244014
C	-5.325750	2.684217	-0.817298
C	-4.940523	1.657222	-1.678417
C	-1.564018	0.713559	-0.253881
C	-0.369310	1.315882	-0.498216
Si	-0.263630	3.116241	-1.116978
C	-0.590496	4.333247	0.294756
C	-1.563286	-0.714578	0.254079
C	-0.367909	-1.315617	0.498406
Si	0.870587	0.000531	-0.000450
C	1.907473	0.589218	1.505291
B	0.913128	1.379300	2.669899
B	1.989408	2.473935	3.537333
B	1.574518	0.902843	4.242545
C	1.587808	-0.154976	2.928240
B	2.922823	-0.211847	3.954031
B	3.100838	-0.423965	2.200783
B	4.186247	0.661778	3.070557
B	3.242422	1.488768	4.335534
B	3.610296	2.326193	2.817997
B	3.519668	1.144594	1.500814

B	2.174477	2.249476	1.790935
C	-2.882562	-1.381652	0.439749
C	-3.283431	-2.394649	-0.433174
C	-4.499134	-3.047885	-0.242371
C	-5.323480	-2.687581	0.819617
C	-4.938233	-1.660579	1.680715
C	-3.728794	-1.004143	1.486756
Si	-0.259752	-3.115300	1.118618
C	-0.582627	-4.334153	-0.292416
C	1.426184	-3.532337	1.859789
C	-1.505137	-3.399585	2.509024
C	1.906415	-0.588201	-1.506923
B	2.172868	-2.248525	-1.792702
B	3.518488	-1.143927	-1.503492
B	3.607994	-2.325587	-2.820699
B	1.986605	-2.473001	-3.538966
B	1.571584	-0.901851	-4.243954
B	2.920315	0.212563	-3.956358
B	3.239299	-1.488131	-4.338021
B	4.184136	-0.661307	-3.073690
B	3.099534	0.424694	-2.203237
C	1.585957	0.156010	-2.929684
B	0.911129	-1.378114	-2.670855
C	-1.508175	3.399311	-2.508376
C	1.422267	3.537105	-1.856055
H	1.840761	3.013815	0.952954
H	1.559381	3.485586	3.982117
H	1.556073	-3.484573	-3.983438
H	-0.396202	-3.896053	-1.277808

H	-1.229238	4.311438	-3.048450
H	1.839494	-3.012758	-0.954479
H	0.064109	5.205653	0.194886
H	-1.618828	-4.685199	-0.265025
H	0.073547	-5.205195	-0.190965
H	-0.404401	3.894607	1.279968
H	1.638818	3.023217	-2.798508
H	5.330264	0.376083	3.187358
H	3.307761	1.469501	-1.698271
H	5.328136	-0.375863	-3.191256
H	4.351287	-3.246538	-2.749934
H	-0.249389	-1.444409	-2.442441
H	3.715127	-1.797122	-5.379031
H	0.882589	0.977911	-2.872788
H	3.052216	1.173527	-4.635401
H	0.799099	-0.686624	-5.114409
H	3.054961	-1.172858	4.632958
H	4.101456	1.160880	0.471904
H	4.353741	3.246986	2.746769
H	3.719004	1.797626	5.376238
H	-0.247526	1.445845	2.442257
H	0.802559	0.687752	5.113501
H	0.884217	-0.976727	2.871801
H	-2.637078	-2.668560	-1.262855
H	-4.800390	-3.837089	-0.924313
H	-6.268390	-3.199212	0.972825
H	-5.582162	-1.372061	2.505460
H	-3.425456	-0.204321	2.157244
H	-2.637833	2.667276	1.263237

H	-4.802052	3.834496	0.925943
H	-6.271041	3.195297	-0.969987
H	-5.584853	1.368165	-2.502659
H	-3.427257	0.201707	-2.155663
H	-1.627339	4.682338	0.266478
H	1.402240	4.610036	-2.083817
H	2.260722	3.368884	-1.176116
H	-1.509226	2.574690	-3.230269
H	-2.530760	3.521152	-2.140295
H	-1.224935	-4.310689	3.050171
H	-1.508517	-2.574339	3.230192
H	-2.527118	-3.523769	2.140059
H	1.407893	-4.605102	2.088485
H	2.265036	-3.363188	1.180564
H	1.640825	-3.017228	2.802016
H	4.100940	-1.160310	-0.474953
H	3.308488	-1.468824	1.695627

Atomic coordinates of the first excited state (S1) of **4** at M06-2X/6-31G*

Energy: –2386.095561

C	-3.919649	0.807953	-1.471783
C	-2.974443	1.347114	-0.589110
C	-3.234992	2.581241	0.017668
C	-4.406262	3.275080	-0.269531
C	-5.326573	2.745990	-1.172299
C	-5.081886	1.509564	-1.768565
C	-1.707822	0.636592	-0.309131
C	-0.417114	1.182383	-0.675314
Si	-0.293916	2.792669	-1.676793
C	-0.539307	4.310735	-0.557941

C	-1.707931	-0.637257	0.307799
C	-0.417391	-1.183171	0.674319
Si	0.845162	-0.000149	0.000189
C	1.951279	0.881990	1.337178
B	0.980837	1.934953	2.303890
B	2.080131	3.167834	2.911230
B	1.635580	1.798899	3.941883
C	1.620092	0.481361	2.884410
B	2.956906	0.618401	3.903599
B	3.127022	0.027399	2.238613
B	4.236722	1.253079	2.854758
B	3.314317	2.354530	3.906982
B	3.693181	2.832664	2.242836
B	3.575510	1.394956	1.217075
B	2.251578	2.559539	1.255056
C	-2.974580	-1.347791	0.587583
C	-3.234800	-2.582004	-0.019201
C	-4.406068	-3.275959	0.267677
C	-5.326703	-2.746921	1.170161
C	-5.082333	-1.510443	1.766436
C	-3.920097	-0.808696	1.469944
Si	-0.294252	-2.793868	1.675128
C	-0.538538	-4.311708	0.555825
C	1.366692	-3.026125	2.541075
C	-1.585913	-2.809843	3.058002
C	1.953128	-0.881640	-1.335625
B	2.254172	-2.559096	-1.253848
B	3.577443	-1.393814	-1.213577
B	3.697257	-2.831002	-2.239809

B	2.085282	-3.166765	-2.910494
B	1.641364	-1.797662	-3.941152
B	2.961975	-0.616442	-3.900608
B	3.320353	-2.352370	-3.904264
B	4.240752	-1.250865	-2.850335
B	3.129563	-0.026049	-2.235159
C	1.623737	-0.480592	-2.883133
B	0.984499	-1.934740	-2.304078
C	-1.584956	2.808035	-3.060211
C	1.367510	3.025810	-2.541581
H	1.927329	3.132480	0.271745
H	1.671960	4.261282	3.123112
H	1.677985	-4.260347	-3.123353
H	-0.358493	-4.095339	-0.501250
H	-1.287305	3.560242	-3.799996
H	1.928854	-3.132779	-0.271339
H	0.172833	5.086459	-0.859822
H	-1.550557	-4.712923	0.662834
H	0.173579	-5.087363	0.857928
H	-0.359902	4.094727	0.499315
H	1.550443	2.328452	-3.365050
H	5.374934	0.976788	3.035628
H	3.317289	1.107815	-1.972578
H	5.379052	-0.973878	-3.029582
H	4.459694	-3.698185	-1.969763
H	-0.176404	-1.982500	-2.072389
H	3.809343	-2.869671	-4.852634
H	0.898540	0.317550	-3.002614
H	3.076373	0.175788	-4.773456

H	0.868834	-1.792943	-4.838177
H	3.070574	-0.173383	4.776943
H	4.158561	1.184043	0.211723
H	4.455505	3.700150	1.973440
H	3.801765	2.872499	4.855783
H	-0.179777	1.981981	2.070581
H	0.861876	1.794161	4.837894
H	0.895193	-0.317154	3.003273
H	-2.518068	-2.986499	-0.728462
H	-4.599576	-4.229375	-0.214338
H	-6.235738	-3.292552	1.402625
H	-5.799485	-1.092404	2.465753
H	-3.727389	0.154312	1.934113
H	-2.518541	2.985777	0.727183
H	-4.600014	4.228448	0.212482
H	-6.235606	3.291526	-1.404994
H	-5.798788	1.091469	-2.468105
H	-3.726684	-0.154994	-1.935969
H	-1.551360	4.711658	-0.665725
H	1.336043	4.029280	-2.984582
H	2.228822	2.998639	-1.871200
H	-1.642535	1.842420	-3.574447
H	-2.587065	3.058591	-2.701390
H	-1.288183	-3.561917	3.797894
H	-1.644039	-1.844245	3.572211
H	-2.587807	-3.060788	2.698855
H	1.335960	-4.029906	2.983416
H	2.228669	-2.997465	1.871631
H	1.547886	-2.329047	3.365182

H	4.159000	-1.183003	-0.207329
H	3.315801	-1.106441	1.976739

Atomic coordinates of **5** at M06-2X/def2-TZVP

Energy: –2515.481199

C	2.278403	2.391747	-1.384183
C	2.413512	1.535694	-0.295345
C	3.652799	1.431172	0.335579
C	4.726280	2.191658	-0.098227
C	4.581275	3.051549	-1.180696
C	3.357634	3.145621	-1.826585
C	1.235377	0.777080	0.196577
C	0.124540	1.367670	0.687815
Si	-0.071801	3.198795	1.088421
C	-1.556759	3.358163	2.219870
C	1.281914	-0.742720	0.174454
C	0.205429	-1.417766	0.632377
Si	-1.011873	-0.064647	1.003942
C	-2.476994	-0.036442	-0.213575
B	-2.063788	0.272213	-1.844713
B	-3.467230	1.107524	-2.533416
B	-3.250883	-0.627533	-2.792890
B	-2.588807	-1.315433	-1.318456
C	-4.241902	-1.351939	-1.621503
B	-3.774348	-1.101044	-0.019896
B	-5.176992	-0.282573	-0.697570
B	-4.853026	0.018032	-2.411573
B	-4.662927	1.320721	-1.235780
B	-3.989169	0.619833	0.246031
B	-2.941866	1.466686	-0.888885

C	2.501110	-1.403686	-0.363119
C	2.721639	-1.429478	-1.738226
C	3.826123	-2.086239	-2.257889
C	4.730560	-2.710923	-1.407969
C	4.530050	-2.666294	-0.035826
C	3.420514	-2.012875	0.483873
Si	0.091443	-3.284471	0.864583
C	0.671745	-4.204386	-0.659957
C	1.115667	-3.790230	2.356312
C	-1.671020	-3.786231	1.262879
Cl	-1.798650	-0.195811	2.903160
C	-0.287792	4.296070	-0.424529
C	1.446899	3.783446	2.018995
H	-2.394556	2.447221	-0.538031
H	-3.341883	1.879685	-3.417778
H	0.976744	-3.091947	3.184121
H	1.272642	4.792676	2.400290
H	-0.883558	5.171607	-0.156020
H	2.178667	-3.847412	2.125639
H	0.794702	-4.777779	2.695392
H	-0.788849	3.794269	-1.251996
H	-1.386113	2.828559	3.158961
H	-6.247885	-0.609348	-0.330194
H	-4.671593	-2.313696	-1.856890
H	-4.146427	1.010247	1.347026
H	-5.393457	2.246761	-1.189976
H	-5.726035	-0.109614	-3.193667
H	-0.928335	0.429137	-2.132061
H	-3.044238	-1.183626	-3.811293

H	-1.908543	-2.274092	-1.291283
H	3.265395	-1.966043	1.554630
H	5.237787	-3.139653	0.632589
H	5.591958	-3.224143	-1.814757
H	3.981305	-2.112486	-3.328611
H	2.013673	-0.943181	-2.399349
H	1.319502	2.461867	-1.883142
H	3.239557	3.806858	-2.675406
H	5.421913	3.642254	-1.520517
H	5.678899	2.115128	0.409580
H	3.767247	0.766614	1.182719
H	0.684064	4.646404	-0.775578
H	-1.733146	4.410336	2.454158
H	-2.470067	2.958574	1.774322
H	1.665979	3.135560	2.869684
H	2.327770	3.813202	1.375357
H	0.458803	-5.270110	-0.546783
H	0.157550	-3.851545	-1.556119
H	1.744982	-4.086095	-0.819019
H	-1.660107	-4.822695	1.608788
H	-2.092687	-3.176414	2.064911
H	-2.338829	-3.732670	0.404431
H	-3.831375	-1.927201	0.814017

Atomic coordinates of **5** at M06-2X/6-31G*

Energy: –2514.935863

C	2.264367	2.405737	-1.396939
C	2.414253	1.549343	-0.303354
C	3.661287	1.458405	0.325137
C	4.726910	2.235225	-0.114205

C	4.566066	3.097549	-1.198250
C	3.335345	3.176834	-1.843078
C	1.241435	0.780614	0.193523
C	0.121100	1.368912	0.683392
Si	-0.076386	3.205696	1.086580
C	-1.560001	3.362837	2.236347
C	1.294731	-0.740035	0.171688
C	0.213161	-1.422865	0.623927
Si	-1.013836	-0.071721	1.000731
C	-2.495791	-0.043839	-0.205516
B	-2.094362	0.278458	-1.841321
B	-3.509178	1.110605	-2.516922
B	-3.282961	-0.624802	-2.788573
B	-2.606870	-1.318139	-1.319583
C	-4.264555	-1.365396	-1.613367
B	-3.788522	-1.118777	-0.010198
B	-5.202293	-0.303584	-0.675175
B	-4.889619	0.009873	-2.392481
B	-4.700377	1.308945	-1.208120
B	-4.012579	0.602098	0.267906
B	-2.975076	1.463468	-0.870579
C	2.522648	-1.399593	-0.355429
C	2.756508	-1.429026	-1.733255
C	3.869073	-2.090415	-2.241755
C	4.767757	-2.715190	-1.378364
C	4.555281	-2.665522	-0.003376
C	3.438284	-2.007094	0.505952
Si	0.109599	-3.298542	0.837131
C	0.689432	-4.197620	-0.712964

C	1.150432	-3.827484	2.321735
C	-1.657756	-3.812888	1.243145
Cl	-1.781986	-0.209871	2.921603
C	-0.304740	4.310881	-0.430449
C	1.456156	3.794185	2.012747
H	-2.428859	2.448907	-0.513900
H	-3.394170	1.891864	-3.400891
H	1.026913	-3.134225	3.160489
H	1.276180	4.795109	2.421538
H	-0.881783	5.199971	-0.152337
H	2.213848	-3.888930	2.077346
H	0.827364	-4.818659	2.658944
H	-0.833087	3.815530	-1.249741
H	-1.374892	2.846067	3.183800
H	-6.273087	-0.641912	-0.303434
H	-4.691383	-2.332379	-1.852871
H	-4.163378	0.986562	1.377094
H	-5.439270	2.233758	-1.152866
H	-5.769883	-0.120465	-3.172838
H	-0.956484	0.444726	-2.134156
H	-3.079890	-1.176633	-3.815246
H	-1.918654	-2.277547	-1.300510
H	3.272004	-1.955639	1.578517
H	5.259165	-3.137941	0.675061
H	5.635025	-3.232109	-1.776942
H	4.035131	-2.119924	-3.314127
H	2.051812	-0.942960	-2.403301
H	1.300004	2.461391	-1.894985
H	3.205623	3.838885	-2.693806

H	5.400455	3.701506	-1.540950
H	5.685502	2.169399	0.390926
H	3.786141	0.792632	1.174254
H	0.668326	4.644471	-0.804188
H	-1.750406	4.417917	2.462540
H	-2.476964	2.946065	1.805686
H	1.697530	3.129223	2.848442
H	2.330772	3.848537	1.356929
H	0.450450	-5.264137	-0.633269
H	0.196878	-3.808481	-1.610316
H	1.770498	-4.099891	-0.854124
H	-1.648466	-4.855741	1.580463
H	-2.078505	-3.210137	2.055806
H	-2.333814	-3.750681	0.386928
H	-3.833297	-1.954446	0.822075

Atomic coordinates of the first excited state (S1) of **5** at M06-2X/6-31G*

Energy: –2514.891929

C	3.572106	-1.718264	0.279642
C	2.520186	-1.199955	-0.484047
C	2.536168	-1.365907	-1.871944
C	3.579282	-2.055636	-2.482757
C	4.618718	-2.578108	-1.715980
C	4.617407	-2.402824	-0.333185
C	1.384378	-0.515336	0.183463
C	0.362139	-1.272156	0.824471
Si	0.409462	-3.087259	1.333083
C	-1.330081	-3.644859	1.786510
C	1.184462	0.888579	0.170323
C	0.058282	1.318179	0.951196

Si	-1.073456	-0.119357	0.975943
Cl	-2.269109	-0.328989	2.661972
C	1.965339	1.839294	-0.640747
C	1.286317	2.858694	-1.326175
C	1.983352	3.785482	-2.091633
C	3.372972	3.722187	-2.170658
C	4.058010	2.718807	-1.488201
C	3.363918	1.778455	-0.735295
Si	-0.015363	2.735849	2.184545
C	1.238792	4.070388	1.746296
C	0.401573	2.066463	3.896312
C	-1.757360	3.444759	2.166450
C	-2.308603	-0.149187	-0.488433
B	-2.619883	1.326397	-1.307018
B	-3.866047	0.546291	-0.327265
B	-4.262998	1.173105	-1.939203
B	-4.270114	-0.193018	-3.062614
B	-4.892293	-0.397936	-1.414596
B	-3.639390	-1.193164	-0.463641
C	-3.828487	-1.528353	-2.108529
B	-2.248241	-1.488291	-1.523844
B	-2.635186	-0.873762	-3.128733
B	-2.863994	0.878539	-3.000806
B	-1.614514	0.069133	-2.036649
C	1.008896	-4.130630	-0.123705
C	1.519331	-3.376077	2.828827
H	-2.110368	2.319215	-0.906386
H	-2.574577	1.601414	-3.894556
H	1.263070	-2.691422	3.643495

H	1.271529	4.800222	2.563828
H	-1.859950	4.224080	2.930035
H	2.577947	-3.245078	2.588512
H	1.384714	-4.400216	3.195070
H	-2.508283	2.674497	2.372051
H	1.398370	1.613823	3.898856
H	-6.020657	-0.697917	-1.223777
H	-4.230575	-2.501031	-2.366581
H	-4.201074	1.001533	0.712369
H	-4.978791	2.108347	-2.071924
H	-5.000879	-0.357400	-3.979131
H	-0.441025	0.211624	-2.134517
H	-2.267655	-1.488013	-4.070977
H	-1.603639	-2.456574	-1.309913
H	3.568255	-1.567325	1.355917
H	5.430501	-2.798616	0.267666
H	5.430548	-3.116402	-2.195002
H	3.579888	-2.186765	-3.560392
H	1.721498	-0.959844	-2.465207
H	0.201354	2.901979	-1.261206
H	1.440704	4.559275	-2.625895
H	3.919843	4.451182	-2.760441
H	5.141205	2.668024	-1.539938
H	3.907967	1.006860	-0.200731
H	-1.988061	3.890649	1.193716
H	0.392582	2.876263	4.634968
H	-0.317782	1.305789	4.212679
H	2.244735	3.657906	1.618045
H	0.977696	4.600097	0.826340

H	0.833679	-5.188772	0.104154
H	0.464582	-3.887895	-1.041663
H	2.076370	-3.991583	-0.318121
H	-1.328777	-4.729358	1.945496
H	-1.690581	-3.173230	2.705039
H	-2.052421	-3.430391	0.991577
H	-3.845366	-1.981838	0.391679

Atomic coordinates of **6** at M06-2X/def2-TZVP

Energy: –2286.842392

C	3.091750	0.932381	2.373308
C	1.797142	0.461875	2.150658
C	0.975875	0.239917	3.263562
C	1.433684	0.469542	4.551711
C	2.728845	0.929516	4.752747
C	3.554597	1.162045	3.663014
Si	0.980541	0.177831	0.494477
C	2.212130	-0.026643	-0.971070
B	1.476604	-0.630961	-2.401188
B	2.144106	0.997019	-2.331804
B	3.553082	0.970085	-1.271618
B	4.777189	-0.080850	-1.998092
B	4.110846	-0.704948	-3.523495
B	3.768703	0.957613	-3.026533
B	2.475804	-0.037983	-3.725665
B	2.693647	-1.688138	-3.133970
B	4.108271	-1.715469	-2.073597
B	3.770279	-0.678012	-0.680210
C	2.549878	-1.546359	-1.463272
C	-0.314735	1.457122	0.084768

Si	-0.155764	3.333225	0.030898
C	-0.865809	4.060253	1.612034
C	-1.452103	0.747214	-0.076067
C	-1.381597	-0.752372	0.128995
C	-0.187824	-1.281047	0.477753
Si	0.113244	-3.059030	1.002005
C	-1.228564	-3.651187	2.166108
C	-2.756729	1.351742	-0.457494
C	-3.236937	1.199785	-1.756248
C	-4.426235	1.800627	-2.138458
C	-5.156687	2.547635	-1.222532
C	-4.696161	2.682868	0.079242
C	-3.501970	2.085029	0.459655
C	-1.018168	4.054763	-1.465483
C	1.655958	3.810708	0.014637
C	-2.614486	-1.562791	-0.040662
C	-3.712974	-1.385321	0.799423
C	-4.828223	-2.197962	0.676214
C	-4.869425	-3.184218	-0.302310
C	-3.791371	-3.349822	-1.159278
C	-2.668884	-2.542918	-1.027536
C	0.207350	-4.239533	-0.466828
C	1.752512	-3.097012	1.916998
H	1.703168	-2.505157	2.833239
H	2.313814	-2.685706	-3.636304
H	0.538002	-3.766128	-1.393813
H	-0.796234	5.122076	-1.538061
H	2.100690	-2.336579	-0.883799
H	-0.506648	5.085055	1.731435

H	-0.777231	-4.670005	-0.657598
H	0.893140	-5.060680	-0.246546
H	-0.539100	3.488980	2.483963
H	2.152360	3.581097	-0.927115
H	3.762748	1.864564	-0.533493
H	5.928799	0.105470	-1.814562
H	4.674326	-2.729256	-1.868101
H	0.327469	-0.890332	-2.364596
H	4.799000	-0.973938	-4.445201
H	1.381100	1.895672	-2.308172
H	4.207445	1.902487	-3.584011
H	1.981970	0.178968	-4.776271
H	-1.824499	-2.666231	-1.694948
H	-3.822228	-4.107099	-1.932137
H	-5.742771	-3.815749	-0.399395
H	-5.668404	-2.062641	1.344790
H	-3.682342	-0.618716	1.563832
H	-3.142112	2.180931	1.476695
H	-5.265886	3.254567	0.800598
H	-6.084117	3.017690	-1.522290
H	-4.783066	1.688549	-3.154008
H	-2.663010	0.620949	-2.470647
H	-1.954836	4.087901	1.603222
H	1.733709	4.888174	0.179133
H	2.204903	3.317998	0.821696
H	-0.673705	3.576442	-2.384416
H	-2.101323	3.933999	-1.406072
H	-0.947601	-4.620592	2.584726
H	-1.364346	-2.953999	2.995465

H	-2.186808	-3.764710	1.656430
H	1.999406	-4.124942	2.191920
H	2.587128	-2.705982	1.329069
H	4.051282	-0.976889	0.423940
H	3.753965	1.125411	1.539867
H	4.563586	1.523346	3.814364
H	3.092111	1.106797	5.756858
H	0.783359	0.288019	5.397801
H	-0.037194	-0.124997	3.118518

Atomic coordinates of **6** at M06-2X/6-31G*

Energy: –2286.251247

C	3.058180	1.026981	2.374776
C	1.790845	0.471143	2.157892
C	1.002545	0.162858	3.279777
C	1.466187	0.393202	4.571074
C	2.733499	0.938858	4.765424
C	3.526606	1.256678	3.666908
Si	0.979483	0.190849	0.493243
C	2.229552	-0.013500	-0.966365
B	1.515428	-0.654790	-2.394961
B	2.155383	0.987921	-2.347756
B	3.559542	1.002766	-1.276131
B	4.808685	-0.041074	-1.975602
B	4.163064	-0.704152	-3.497245
B	3.788482	0.963993	-3.031544
B	2.515458	-0.067073	-3.723810
B	2.757440	-1.705799	-3.100633
B	4.166999	-1.690479	-2.027238
B	3.800596	-0.632344	-0.652679

C	2.597297	-1.537254	-1.429255
C	-0.328657	1.463428	0.074183
Si	-0.191571	3.346218	0.013523
C	-0.901162	4.075194	1.605689
C	-1.468232	0.744347	-0.080964
C	-1.388725	-0.755524	0.123596
C	-0.185124	-1.278959	0.470546
Si	0.119351	-3.064625	0.987541
C	-1.235060	-3.673770	2.145394
C	-2.780262	1.346778	-0.451773
C	-3.269501	1.202751	-1.753285
C	-4.463949	1.810032	-2.125605
C	-5.190142	2.554670	-1.197479
C	-4.722136	2.680185	0.107704
C	-3.523390	2.075538	0.479121
C	-1.077546	4.056916	-1.487646
C	1.624307	3.844027	-0.018509
C	-2.616322	-1.578072	-0.051218
C	-3.718543	-1.422128	0.796174
C	-4.826175	-2.252177	0.667724
C	-4.855396	-3.234439	-0.321574
C	-3.774571	-3.377661	-1.186971
C	-2.660649	-2.552194	-1.052054
C	0.229566	-4.244407	-0.492497
C	1.759091	-3.107589	1.923452
H	1.688709	-2.551532	2.864237
H	2.397018	-2.722960	-3.588900
H	0.608783	-3.775636	-1.407667
H	-0.838920	5.121578	-1.589158

H	2.156744	-2.328804	-0.835848
H	-0.546191	5.104539	1.728565
H	-0.763179	-4.644375	-0.722231
H	0.883687	-5.090544	-0.254774
H	-0.570944	3.502656	2.479572
H	2.117536	3.608143	-0.964720
H	3.748985	1.917460	-0.549369
H	5.960127	0.169354	-1.787154
H	4.749416	-2.695390	-1.799079
H	0.366169	-0.935286	-2.360715
H	4.865456	-0.978138	-4.412705
H	1.374718	1.878778	-2.343388
H	4.217212	1.909905	-3.604589
H	2.025072	0.123652	-4.786272
H	-1.814309	-2.655152	-1.726298
H	-3.796974	-4.130728	-1.968849
H	-5.722233	-3.880192	-0.421180
H	-5.669306	-2.133731	1.341166
H	-3.694967	-0.659658	1.569761
H	-3.156146	2.163067	1.498118
H	-5.289380	3.248690	0.838456
H	-6.121152	3.029916	-1.490153
H	-4.828119	1.704624	-3.142824
H	-2.697444	0.626305	-2.475925
H	-1.994008	4.097364	1.599434
H	1.701479	4.926224	0.137111
H	2.184787	3.359248	0.790161
H	-0.762008	3.553179	-2.407358
H	-2.164260	3.956314	-1.402879

H -0.938029 -4.630114 2.590494

H -1.406528 -2.963670 2.961584

H -2.183959 -3.820993 1.620379

H 2.025345 -4.142694 2.165121

H 2.598463 -2.676558 1.364169

H 4.075055 -0.907375 0.464706

H 3.693362 1.284369 1.532637

H 4.513970 1.683502 3.813377

H 3.100995 1.116190 5.771575

H 0.842475 0.144415 5.424254

H 0.012610 -0.270427 3.139430

Atomic coordinates of the first excited state (S1) of **6** at M06-2X/6-31G*

Energy: –2286.204880

C -2.343917 -2.746400 0.704270

C -2.542933 -1.365969 0.856856

C -3.827109 -0.906133 1.186759

C -4.872751 -1.804977 1.365391

C -4.662900 -3.172989 1.203505

C -3.394377 -3.640787 0.866403

C -1.403109 -0.445331 0.694868

C -0.111943 -0.711752 1.275061

Si 0.133519 -1.076260 3.101067

C 1.958254 -1.358045 3.472217

C -1.443026 0.742930 -0.079516

C -2.679002 1.296225 -0.688077

C -3.273935 0.681711 -1.793960

C -4.400703 1.244705 -2.385804

C -4.949893 2.418702 -1.874275

C -4.373045 3.027149 -0.760868

C	-3.242554	2.468685	-0.172256
C	-0.186742	1.359239	-0.293110
Si	0.190101	3.040653	-1.067613
C	2.028456	3.136591	-1.470380
Si	1.134548	0.143473	0.214464
C	1.662306	-1.017933	-1.244455
C	1.355980	-2.611865	-1.066663
B	2.963780	-2.096318	-0.974110
B	2.513134	-3.591444	-1.809379
B	0.901757	-3.361300	-2.508317
B	0.997997	-2.068578	-3.715095
B	2.683896	-1.501348	-3.761570
B	2.340543	-3.224534	-3.531900
B	3.623056	-2.440309	-2.575362
B	3.065083	-0.804079	-2.180034
B	1.459083	-0.577247	-2.876490
B	0.357729	-1.722716	-2.108066
C	2.579786	0.961241	1.075490
C	3.946051	0.681304	0.943196
C	4.896261	1.384251	1.680713
C	4.501342	2.384327	2.565150
C	3.148571	2.685385	2.709077
C	2.203199	1.979745	1.971658
C	-0.210072	4.491988	0.070457
C	-0.802479	3.202780	-2.666221
C	-0.540830	0.348672	4.137204
C	-0.824207	-2.630496	3.587741
H	2.528617	-0.425045	3.502156
H	0.101017	-4.231856	-2.450855

H	-0.528309	-3.503100	2.997398
H	-0.427972	4.064094	-3.231806
H	0.905917	-2.874587	-0.113981
H	0.143120	5.419329	-0.395422
H	-1.904064	-2.497182	3.475167
H	-0.611981	-2.847050	4.641860
H	0.296498	4.394930	1.036249
H	2.390054	2.245735	-1.995895
H	3.745488	0.138902	-1.950235
H	4.776609	-2.685035	-2.699973
H	2.793493	-4.615306	-1.285522
H	-0.744756	-1.481834	-1.754742
H	2.582120	-4.045922	-4.352667
H	1.053104	0.513319	-3.104164
H	3.176469	-1.065914	-4.748739
H	0.275159	-2.046059	-4.654769
H	-1.352163	-3.109638	0.447578
H	-3.222213	-4.704308	0.732897
H	-5.483220	-3.870594	1.339865
H	-5.856934	-1.433851	1.634060
H	-3.998319	0.157405	1.319552
H	-2.791781	2.929982	0.702559
H	-4.803581	3.935846	-0.351334
H	-5.827851	2.856049	-2.339294
H	-4.849635	0.766651	-3.250874
H	-2.841016	-0.231216	-2.193341
H	-1.283275	4.598133	0.249772
H	2.204468	3.996763	-2.126817
H	2.641131	3.271019	-0.573441

H	-0.695592	2.312993	-3.294067
H	-1.868279	3.354081	-2.470538
H	-0.545600	0.087077	5.201240
H	0.062921	1.253024	4.014386
H	-1.569652	0.581107	3.841070
H	2.058929	-1.846310	4.448164
H	2.424205	-2.012371	2.726406
H	3.474644	-2.097169	0.093740
H	4.285575	-0.087459	0.259609
H	5.949351	1.149089	1.561740
H	5.245214	2.929256	3.138405
H	2.831136	3.466620	3.393098
H	1.148243	2.221941	2.089698

Atomic coordinates of **7** at M06-2X/def2-TZVP

Energy: –2286.865820

C	3.142982	0.558499	2.417379
C	1.820463	0.194980	2.163789
C	0.976720	-0.022534	3.260749
C	1.438725	0.107882	4.561005
C	2.761962	0.462440	4.791870
C	3.611017	0.689491	3.719323
Si	0.994664	0.043951	0.492357
C	2.207899	-0.101064	-0.988576
B	1.463111	-0.490686	-2.484819
B	2.130144	1.100209	-2.184947
B	3.549570	0.914908	-1.148120
B	4.760598	-0.003076	-2.036289
B	4.083521	-0.393344	-3.624295
C	3.687692	1.046296	-2.822438

B	2.456410	0.296646	-3.713456
B	2.669761	-1.425756	-3.385783
B	4.099274	-1.611729	-2.347717
B	3.763550	-0.794294	-0.812181
B	2.481923	-1.660137	-1.648503
C	-0.246963	1.411143	0.190843
Si	-0.032398	3.273776	0.329213
C	-0.740267	3.862183	1.967768
C	-1.410744	0.765577	-0.038688
C	-1.410485	-0.746798	0.064698
C	-0.246418	-1.348745	0.391723
Si	-0.034788	-3.154880	0.876890
C	-1.373530	-3.623524	2.105446
C	-2.678646	1.456403	-0.399878
C	-3.113825	1.447901	-1.723009
C	-4.262971	2.132194	-2.088064
C	-4.998208	2.821678	-1.131875
C	-4.584020	2.813601	0.192358
C	-3.430777	2.131082	0.555957
C	-0.842440	4.186664	-1.091962
C	1.792761	3.708797	0.390241
C	-2.678898	-1.484369	-0.167483
C	-3.787493	-1.292971	0.657065
C	-4.942957	-2.032167	0.463423
C	-5.013909	-2.959575	-0.569522
C	-3.923617	-3.141570	-1.407045
C	-2.761783	-2.407773	-1.205544
C	-0.126693	-4.358651	-0.566718
C	1.624065	-3.312669	1.739897

H	1.631553	-2.751917	2.676891
H	2.342027	-2.245966	-4.169504
H	0.251323	-3.938419	-1.498649
H	-0.573827	5.244993	-1.050422
H	1.993857	-2.597342	-1.132232
H	-0.361387	4.862881	2.188158
H	-1.158968	-4.673150	-0.728145
H	0.464035	-5.248733	-0.337761
H	-0.433373	3.202014	2.782162
H	2.292498	3.608969	-0.572210
H	3.806124	1.800868	-0.418870
H	5.893348	0.299892	-1.916039
H	4.795647	-2.564196	-2.386229
H	0.289850	-0.616684	-2.530340
H	4.781081	-0.350922	-4.574041
H	1.503894	2.090816	-2.091103
H	4.087570	1.973415	-3.204007
H	2.061634	0.799532	-4.703696
H	-1.906325	-2.546104	-1.855479
H	-3.974392	-3.855807	-2.218895
H	-5.918331	-3.534038	-0.721663
H	-5.790956	-1.885913	1.119811
H	-3.733897	-0.574722	1.465702
H	-3.109429	2.113739	1.590115
H	-5.158751	3.338627	0.944613
H	-5.894042	3.357124	-1.417778
H	-4.584814	2.129816	-3.121371
H	-2.536293	0.912402	-2.467678
H	-1.828157	3.913852	1.954101

H	1.889726	4.749912	0.707454
H	2.324628	3.094044	1.121224
H	-0.511669	3.792163	-2.054831
H	-1.930412	4.108725	-1.053008
H	-1.170516	-4.619864	2.505931
H	-1.404618	-2.925275	2.944545
H	-2.360080	-3.640683	1.638900
H	1.821296	-4.361176	1.974717
H	2.452833	-2.947759	1.129431
H	4.136340	-1.139484	0.252191
H	3.826236	0.741970	1.599032
H	4.642478	0.967573	3.893733
H	3.128674	0.561321	5.805475
H	0.770124	-0.069819	5.393596
H	-0.058411	-0.306620	3.091895

Atomic coordinates of **7** at M06-2X/6-31G*

Energy: –2286.274458

C	-2.753521	-2.421479	-1.210172
C	-2.684077	-1.492272	-0.169058
C	-3.803011	-1.306729	0.651003
C	-4.954411	-2.059495	0.451535
C	-5.010441	-2.994436	-0.581505
C	-3.910516	-3.169699	-1.415856
C	-1.417929	-0.746814	0.067965
C	-0.247078	-1.347574	0.399396
Si	-0.041578	-3.156331	0.899281
C	1.619371	-3.315756	1.779543
C	-1.420085	0.765716	-0.043385
C	-2.691976	1.458260	-0.399341

C	-3.133985	1.453692	-1.725353
C	-4.284960	2.146768	-2.085126
C	-5.014998	2.840354	-1.121277
C	-4.595361	2.827392	0.206205
C	-3.440673	2.135890	0.565284
C	-0.250498	1.415857	0.178487
Si	-0.045738	3.285644	0.304523
C	1.786661	3.732480	0.350675
Si	0.998584	0.049890	0.493381
C	2.218019	-0.105583	-0.990870
B	2.505983	-1.674630	-1.629063
B	3.782639	-0.785171	-0.802695
B	4.126739	-1.624445	-2.328212
B	2.694126	-1.464403	-3.372621
B	2.467077	0.254676	-3.726174
C	3.694303	1.029539	-2.843850
B	4.102377	-0.422952	-3.624848
B	4.776558	-0.003584	-2.039454
B	3.555367	0.919249	-1.165460
B	2.132356	1.078868	-2.206774
B	1.477374	-0.523875	-2.485646
C	1.827499	0.209133	2.167833
C	3.142236	0.623780	2.411956
C	3.618473	0.767979	3.714038
C	2.785574	0.502873	4.796564
C	1.470852	0.096510	4.575892
C	1.000199	-0.047500	3.274921
C	-0.748595	3.881219	1.953957
C	-0.875398	4.190448	-1.124611

C	-1.395210	-3.612909	2.131960
C	-0.129555	-4.381032	-0.539072
H	1.615851	-2.767947	2.727875
H	2.373044	-2.301948	-4.147728
H	0.286092	-3.981069	-1.468276
H	-0.583661	5.246757	-1.115970
H	2.022694	-2.611581	-1.095751
H	-0.369962	4.885217	2.176110
H	-1.168237	-4.670245	-0.726865
H	0.432169	-5.287156	-0.285469
H	-0.438940	3.219522	2.770503
H	2.282744	3.621486	-0.616832
H	3.804380	1.822968	-0.447370
H	5.910842	0.312900	-1.923597
H	4.833096	-2.575826	-2.353222
H	0.300234	-0.660293	-2.529897
H	4.803283	-0.387338	-4.578093
H	1.498100	2.071705	-2.125443
H	4.088968	1.957886	-3.239965
H	2.069905	0.742595	-4.728317
H	-1.890504	-2.551784	-1.857748
H	-3.950257	-3.888871	-2.228417
H	-5.911303	-3.579915	-0.736931
H	-5.810558	-1.917764	1.103845
H	-3.759040	-0.583532	1.460391
H	-3.113174	2.114257	1.601253
H	-5.166678	3.354823	0.964139
H	-5.911927	3.382691	-1.403672
H	-4.612411	2.147872	-3.120185

H	-2.558970	0.915451	-2.474687
H	-1.840321	3.931010	1.944213
H	1.888685	4.780062	0.656518
H	2.327171	3.124620	1.086333
H	-0.578458	3.766790	-2.089923
H	-1.966591	4.136721	-1.056793
H	-1.187926	-4.600003	2.560974
H	-1.443205	-2.893990	2.957207
H	-2.380370	-3.649564	1.656345
H	1.826738	-4.368541	2.002155
H	2.454631	-2.933533	1.182343
H	4.155716	-1.110701	0.273353
H	3.811670	0.836031	1.584146
H	4.643221	1.085641	3.880481
H	3.158526	0.612349	5.810365
H	0.815808	-0.111733	5.416408
H	-0.027286	-0.373192	3.113899

Atomic coordinates of the first excited state (S1) of **7** at M06-2X/6-31G*

Energy: –2286.230931

C	-2.437206	-2.696750	0.503856
C	-2.595484	-1.336912	0.807164
C	-3.855413	-0.876516	1.214404
C	-4.924100	-1.759577	1.325927
C	-4.757140	-3.108559	1.019652
C	-3.511359	-3.573265	0.602567
C	-1.432764	-0.432931	0.704207
C	-0.158118	-0.752416	1.277705
Si	0.150650	-1.411703	3.007352
C	1.975310	-1.829858	3.209278

C	-1.440861	0.785200	-0.017824
C	-2.662197	1.375961	-0.620225
C	-3.248308	0.808592	-1.755837
C	-4.369054	1.396021	-2.335220
C	-4.922463	2.548754	-1.782061
C	-4.355363	3.110523	-0.639580
C	-3.230785	2.527420	-0.063572
C	-0.164811	1.390812	-0.175949
Si	0.245956	3.126107	-0.778065
C	2.084237	3.233612	-1.186283
Si	1.115887	0.104312	0.250611
C	1.613098	-1.004207	-1.255946
B	1.317689	-2.695591	-1.151050
B	2.960113	-2.059502	-1.169344
B	2.541587	-3.456499	-2.176952
B	0.890188	-3.206887	-2.789454
B	0.901631	-1.764891	-3.817230
C	2.498293	-1.191694	-3.718212
B	2.285009	-2.873654	-3.826149
B	3.567713	-2.166539	-2.826760
B	2.968450	-0.631472	-2.197756
B	1.326891	-0.384197	-2.808156
B	0.306914	-1.655954	-2.156867
C	2.584158	0.837146	1.157480
C	3.947845	0.607116	0.933543
C	4.920345	1.257117	1.690607
C	4.550390	2.153212	2.689749
C	3.200476	2.401110	2.929737
C	2.233344	1.749936	2.170427

C	-0.112410	4.466131	0.502412
C	-0.732718	3.485530	-2.355400
C	-0.373912	-0.129789	4.289033
C	-0.863394	-2.973654	3.318986
H	2.601754	-0.937458	3.299177
H	0.142503	-4.103501	-2.996041
H	-0.634724	-3.762547	2.596245
H	-0.340281	4.397864	-2.820031
H	0.885131	-3.137047	-0.138352
H	0.224270	5.435737	0.117315
H	-1.939051	-2.779246	3.279527
H	-0.615765	-3.346410	4.320499
H	0.429374	4.273621	1.434273
H	2.413520	2.415169	-1.835988
H	3.633808	0.334666	-2.049087
H	4.703454	-2.239075	-3.151472
H	2.982506	-4.531519	-1.942394
H	-0.807361	-1.401613	-1.845755
H	2.576335	-3.427215	-4.831164
H	0.992975	0.729804	-3.022089
H	2.897880	-0.666640	-4.577850
H	0.270337	-1.570406	-4.799062
H	-1.461831	-3.052627	0.181384
H	-3.374803	-4.621742	0.355928
H	-5.594485	-3.794206	1.104818
H	-5.891342	-1.391808	1.654543
H	-3.989450	0.173609	1.455649
H	-2.789433	2.951918	0.834290
H	-4.789569	4.001731	-0.196677

H	-5.796638	3.004849	-2.236217
H	-4.811672	0.952282	-3.221683
H	-2.814532	-0.090382	-2.184737
H	-1.177622	4.551632	0.733458
H	2.277017	4.172726	-1.718093
H	2.710124	3.223130	-0.288581
H	-0.637355	2.668879	-3.077837
H	-1.797644	3.631319	-2.152758
H	-0.327111	-0.552235	5.299135
H	0.273682	0.751930	4.261921
H	-1.403485	0.195500	4.105119
H	2.115353	-2.432100	4.114140
H	2.342636	-2.417623	2.359878
H	3.625016	-2.094867	-0.190161
H	4.268394	-0.084324	0.163509
H	5.970772	1.061626	1.497886
H	5.310787	2.657704	3.278195
H	2.901923	3.100389	3.705008
H	1.180968	1.954020	2.360387

References

[35] S. Yamaguchi, R.-Z. Jin, K. Tamao, M. Shiro, *Organometallics*, **1997**, *16*, 2230–2232.

[36] W.-C. Joo, J.-H. Hong, S.-B. Choi, H.-E. Son, C. H. Kim, *J. Organomet. Chem.*, **1990**, *391*, 27–36.

[53] M. J. Frisch, G. W. Trucks, H. B. Schlegel, G. E. Scuseria, M. A. Robb, J. R. Cheeseman, G. Scalmani, V. Barone, G. A. Petersson, H. Nakatsuji, X. Li, M. Caricato, A. V. Marenich, J. Bloino, B. G. Janesko, R. Gomperts, B. Mennucci, H. P. Hratchian, J. V. Ortiz, A. F. Izmaylov, J. L. Sonnenberg, D. Williams-Young, F. Ding, F. Lipparini, F. Egidi, J. Goings, B. Peng, A. Petrone, T. Henderson, D. Ranasinghe, V. G. Zakrzewski, J. Gao, N. Rega, G. Zheng, W. Liang, M. Hada, M. Ehara, K. Toyota, R. Fukuda, J. Hasegawa, M. Ishida, T. Nakajima, Y. Honda, O. Kitao, H. Nakai, T. Vreven, K. Throssell, J. A. Montgomery Jr., J. E. Peralta, F. Ogliaro, M. J. Bearpark, J. J. Heyd, E. N. Brothers, K. N. Kudin, V. N. Staroverov, T. A. Keith, R. Kobayashi, J. Normand, K. Raghavachari, A. P. Rendell, J. C. Burant, S. S. Iyengar, J. Tomasi, M. Cossi, J. M. Millam, M. Klene, C. Adamo, R. Cammi, J. W. Ochterski, R. L. Martin, K. Morokuma, O. Farkas, J. B. Foresman, D. J. Fox, Gaussian 16 (Revision C.01), Gaussian Inc., Wallingford, CT, **2016**.

[54] A. T. B. Gilbert, IQmol (2.15.3.), Q-Chem Inc., Pleasanton, CA, **2021**.

[55] N. M. O'Boyle, A. L. Tenderholt, K. M. Langner, *J. Comp. Chem.*, **2008**, *29*, 839–845.

[56] P. R. Spackman, M. J. Turner, J. J. McKinnon, S. K. Wolff, D. J. Grimwood, D. Jayatilaka, M. A. Spackman, *J. Appl. Cryst.*, **2021**, *3*, 1006–1011.

[57] G. M. Sheldrick, *Acta Cryst.*, **2015**, *C71*, 3–8.

[58] O. V. Dolomanov, L. J. Bourhis, R. J. Gildea, J. A. K. Howard, H. Puschmann, *J. Appl. Cryst.*, **2020**, *53*, 226–235.

[59] G. M. Sheldrick, *Acta Cryst.*, **2015**, *A71*, 3–8.

[60] A. L. Spek, *Acta Cryst.*, **2020**, *E76*, 1–11.

[61] C. F. Macrae, I. Sovago, S. J. Cottrell, P. T. A. Galek, P. McCabe, E. Pidcock, M. Platings, G. P. Shields, J. S. Stevens, M. Towler, P. A. Wood, *J. Appl. Cryst.*, **2020**, *53*, 226–235.
